# Effect of carbohydrate restriction on body weight in overweight and obese adults: a systematic review and dose–response meta-analysis of 110 randomized controlled trials

**DOI:** 10.3389/fnut.2023.1287987

**Published:** 2023-12-06

**Authors:** Sepideh Soltani, Ahmad Jayedi, Shima Abdollahi, Azam Ahmadi Vasmehjani, Fatemeh Meshkini, Sakineh Shab-Bidar

**Affiliations:** ^1^Yazd Cardiovascular Research Center, Non-communicable Diseases Research Institute, Shahid Sadoughi University of Medical Sciences, Yazd, Iran; ^2^Social Determinants of Health Research Center, Semnan University of Medical Sciences, Semnan, Iran; ^3^Department of Nutrition, School of Health, North Khorasan University of Medical Sciences, Bojnurd, Iran; ^4^Department of Nutrition, School of Public Health, Shahid Sadoughi University of Medical Sciences, Yazd, Iran; ^5^Department of Biochemistry, School of Medicine, Shahid Sadoughi University of Medical Sciences, Yazd, Iran; ^6^Department of Community Nutrition, School of Nutritional Sciences and Dietetics, Tehran University of Medical Sciences, Tehran, Iran

**Keywords:** low-carbohydrate diet, carbohydrate restriction, obesity, weight loss, ketogenic diet, body weight

## Abstract

**Introduction:**

Carbohydrate-restricted diets are one of the most effective dietary interventions for weight loss. However, the optimum carbohydrate intake for implementing the most effective weight-loss interventions is still being discussed. We aimed to determine the optimum carbohydrate intake for short- and long-term weight loss in adults with overweight and obesity.

**Methods:**

We searched PubMed, Scopus, Web of Science, and CENTRAL from inception to May 2021 for randomized controlled trials examining the effect of a carbohydrate-restricted diet (≤45% of energy intake) as compared to a control diet (carbohydrate intake >45% of energy intake) on body weight in adults with overweight/obesity. A random-effects dose–response meta-analysis was conducted to calculate the mean difference for each 10% decrease in carbohydrate intake at the 6-month follow-up (1 to 6 months), 12-month follow-up (6 to 12 months), and follow-up longer than 12 months. The shape of the dose-dependent effects was also evaluated. The certainty of the evidence was rated using the GRADE approach. The minimal clinically important difference (MCID) threshold was defined as 5% weight loss (equal to 4.39 kg).

**Results:**

A total of 110 trials were selected for the present meta-analysis. In the linear dose–response meta-analysis, each 10% decrease in carbohydrate intake reduced body weight by 0.64 kg (95% CI: −0.79 to −0.49; *n* = 101 trials with 4,135 participants, high-certainty evidence) at the 6-month follow-up and by 1.15 kg (95% CI: −1.61 to −0.69; 42 trials with 2,657 participants, moderate-certainty evidence) at the 12-month follow-up. Non-linear dose–response meta-analyses indicated a monotonic reduction in body weight with the decrease in carbohydrate intake, with the greatest reduction at 5% at the 6-month follow-up (mean difference 5%: −3.96 kg, 95% CI: −4.92 to −3.00) and 10% at the 12-month follow-up (mean difference 10%: −6.26 kg, 95% CI: −10.42 to −2.10). At follow-up longer than 12 months, dose–response analyses suggested a non-linear effect, wherein carbohydrate intakes higher than 40% and lower than 30% were not effective for weight loss.

**Discussion:**

Carbohydrate restriction is an effective dietary strategy for important weight loss in adults with overweight and obesity. At 6-month and 12-month follow-ups, body weight decreased proportionally, more than the MCID threshold, along with the decrease in carbohydrate intake. At follow-up longer than 12 months, there was a non-linear effect, with the greatest reduction at 30% carbohydrate intake.

**Systematic review registration:**

https://www.crd.york.ac.uk/prospero/, identifier CRD42022315042.

## Introduction

The prevalence of obesity has reached a pandemic status over the past decades (1). Due to the well-established role of obesity in increasing the incidence of comorbid conditions and mortality (2–5), adiposity has been placed as the top health priority over the past three decades (1). A higher energy intake-to-expenditure ratio has a major influence on increasing body weight and the subsequent risk of adiposity (6). Therefore, lifestyle modifications, including calorie-restricted diets, are suggested as the first-choice treatment for obesity management (7, 8). Some pieces of evidence, however, suggest that variations in dietary macronutrient composition, independent of energy intake, may favorably affect body weight (9–11).

Restricted-carbohydrate diets have been a popular dietary approach to reduce energy intake and body weight. An extensive body of evidence presented by a large number of systematic reviews and pairwise meta-analyses of intervention studies supports the fact that carbohydrate-restricted diets are effective interventions for short-term weight loss in adults (12–22). Carbohydrate-restricted diets can reduce body weight by reducing appetite via the production of ketone bodies, increasing energy expenditure and insulin sensitivity, and stimulating lipolysis (23). Typically, a carbohydrate-restricted diet is defined as a diet with a carbohydrate intake of less than 45% of total energy intake (24). Three well-described categories of carbohydrate-restricted diets include very low-carbohydrate or ketogenic diets (≤10% of total calorie or 20–50 g/day), low-carbohydrate diets (10–26% of total calorie or 50–130 g/day), and moderate-carbohydrate diets (26–45% of total calorie or 130–230 g/day) (25).

A recent network meta-analysis of randomized trials suggested that carbohydrate-restricted diets are superior to other structured dietary programs, such as low-fat diets, in reducing body weight in adults with overweight and obesity (26). However, despite the large body of evidence, the optimum degree of carbohydrate restriction for implementing the most effective weight-loss interventions in adults with overweight and obesity is still unclear.

A recent dose–response meta-analysis of 50 randomized trials suggested a linear reduction in body weight in parallel to the decrease in carbohydrate intake from 65 to 10% in patients with type 2 diabetes (27); however, the dose-dependent effect of carbohydrate restriction on body weight in adults with overweight and obesity is still being discussed. It is not well determined how well body weight changes with the decrease in carbohydrate intake at the short- and long-term follow-ups. In addition, previous meta-analyses have mainly failed to control for potential effect modification by calorie restriction, exercise programs, and protein intake. Thus, this study aimed to evaluate the potential dose-dependent effects of carbohydrate restriction on body weight by performing a dose–response meta-analysis of randomized controlled trials (RCTs) of carbohydrate-restricted diets in adults with overweight and obesity.

## Methods

### Protocol

This systematic review followed the guidelines from the Cochrane Handbook for Systematic Reviews of Interventions (28) and adhered to the Preferred Reporting Items for Systematic Reviews and Meta-Analyses (PRISMA) statement (29). The review protocol was registered in the International Prospective Register of Systematic Reviews (PROSPERO, registration number CRD42022315042) (30).

### Data sources and searches

We searched PubMed, Scopus, Web of Science, and CENTRAL to May 2021 with a predefined combination of text-word and medical subject heading (MeSH) terms provided in Supplementary Table S1. We did not place restrictions on keywords in terms of outcomes to include all potential eligible trials with weight loss as either a primary or secondary outcome. The reference lists of the previous reviews and eligible primary trials were also searched to identify further relevant trials. Title, abstract, and full-text articles were screened according to the pre-specified inclusion and exclusion criteria by the two independent investigators (SS and AJ). Discrepancies were resolved by consensus.

### Study selection

Original RCTs (either parallel or cross-over designs) were considered eligible for inclusion in the present meta-analysis if they (1) were conducted in adults with overweight or obesity (body mass index [BMI] ≥25 kg/m^2^) aged ≥18 years, regardless of their health status; (2) the intervention duration was continued for at least 1 month; (3) compared the effect of a carbohydrate-restricted diet (carbohydrate intake ≤45% of total energy intake or ≤ 230 g/day) (25, 31), with or without calorie restriction or exercise program, with a control group (wait list controls or any active controls including competing dietary programs higher in carbohydrate [>45%]); (4) reported amount of carbohydrate intake (% calorie or g/day) in both intervention and control groups; and (5) reported mean and standard deviation (SD) of change in body weight across study arms, or reported adequate information to estimate those values. We excluded trials if they (1) were conducted in children or adolescents, pregnant/lactating women, professional athletes, or patients undergoing bariatric surgery; (2) were editorial, commentary, review, or case-report studies; (3) did not consider control groups (repeated design); (4) prescribed any co-intervention other than calorie restriction or exercise program in the intervention or control group (e.g., weight-loss medications); (5) did not report sufficient data to calculate the amount of carbohydrate intake (% calorie) in study arms; (6) enrolled mix population in terms of body weight (normal, overweight, and obese); and (7) performed meal replacement rather than implementing a structured dietary program.

### Data extraction

Two authors (AA and FM) independently extracted the following information by reviewing the full texts of the eligible trials: first author’s name, the study’s year of publication, study location, age and sex of participants, study design (parallel/cross-over), intervention duration (month), number of participants in the intervention and control groups, the dose of dietary carbohydrate in the form of grams per day or % calorie in each study arm, health status of the study participants, macronutrient composition of the diet (% calorie) in intervention and control groups, any other interventions (i.e., calorie restriction, physical activity) that were prescribed in either intervention or control groups, side effects, degree of adherence to the study protocol, and mean and SD of body weight before and after the intervention or mean difference and SD of change in body weight during the follow-up period in each study arm.

### Quality assessment

The Cochrane tool for risk of bias assessment was used to assess the methodological quality of the trials (32). Each study was given a quality score of low, high, or unclear based on the five following domains: (1) random sequence generation, (2) allocation concealment (both of them assessed selection bias), (3) blinding of participants and personnel (assessed performance bias), (4) blinding of outcome assessors (assessed detection bias), (5) incomplete outcome data (assessed attrition bias), and (6) selective reporting (assessed reporting bias) (Supplementary Text S1). Accordingly, trials were rated as having a low risk of bias (if all six domains were rated as low risk), some concerns (if one of the first four domains and one from another domain were rated unclear), or a high risk of bias (if at least one domain was rated high risk, two of the first four domains or more than three domains were rated unclear).

### Data synthesis and statistical analysis

The mean differences in body weight between the intervention (carbohydrate-restricted diet) and control groups (high-carbohydrate diet) and their 95% confidence intervals (CIs) were used as the effect size in the present meta-analysis. First, we calculated the change in body weight from baseline values in each study arm in each trial. For trials that did not report the mean values and SDs of changes in text or graphs, we calculated these values using data from measures before and after the intervention, according to the instructions outlined in the Cochrane Handbook (33). Regarding the studies in which the SDs of the change were not reported in the trials, we derived them using the following formula: SD changes = square root [(SD baseline 2 + SD final 2) − (2 × R × SD baseline × SD final)] with a correlation coefficient of 0.89 (34). The correlation coefficient was estimated based on studies that reported the SD values of baseline weights, final weights, and changes from baseline. For trials wherein change in body weight was reported in forms other than mean and SD (e.g., median, ranges, or interquartile range), we converted them to mean and SD by the formula suggested by Wan et al. (35).

Using a one-stage approach, a non-linear dose–response (36) association was detected between a change in body weight and a 10% decrease in dietary carbohydrate intake in the intervention group versus the control group. This method required the dose of dietary carbohydrate (% calorie), the number of participants, and the mean and SD of the change in body weight in each study arm. Assuming the highest dose (65% carbohydrate at 6-month follow-up and 55% at 12-month follow-up) as a reference, we computed point-specific (65, 60, 55, 50, 45, 40, 35, 30, 25, 20, 15, 10, and 5%) estimates and 95% CIs. Due to the lack of assumptions regarding the shape of the dose–response relationship, restricted cubic splines with three knots were used at Harrell’s recommended percentiles (10, 50, and 90%) (36). Maximum likelihood estimation was used to estimate the measures. The Wald test was used to assess deviations from linearity. The significance of the Wald test determined that a non-linear model was the best fit.

The dose-dependent effects of carbohydrate restriction on body weight were evaluated separately across three time periods, including 1 to ≤6 months (6-month follow-up), 6 to ≤12 months (12-month follow-up), and follow-up >12 months. Trial-specific results were pooled using the DerSimonian and Laird random-effects model (37). If trials reported the amount of carbohydrates as g/day, we converted g/day to %calorie using the average calorie intake reported in that trial. For trials that reported carbohydrate intake as a range, the midpoint of the lower and upper bounds of dietary carbohydrate intake was used. When the content of dietary carbohydrates was not reported directly (in the form of %calorie or g/day), we subtracted the sum of the calories from fat and protein from total energy intake to calculate the % calorie obtained from carbohydrates. The amount of dietary carbohydrates that was extracted for the analyses was based on actual (self-reported) dietary intake in each trial unless trials reported only the prescribed dietary carbohydrate. The Q-test was used to investigate the level of heterogeneity, and the I^2^ index was used to measure its magnitude. Statistical heterogeneity was assessed using I^2^ statistics: 0% suggested no heterogeneity, 0–25% low heterogeneity, 25–50% low heterogeneity, 50–75% moderate heterogeneity, and a value of 75% suggested high heterogeneity (38, 39). We performed predefined subgroup analyses based on the sex (male and female) study design (parallel and cross-over), study quality (low risk, some concerns, and high risk), health stats (healthy versus unhealthy), the proportion of calories obtained from protein intake in the intervention arm (≤20%, 20–24.9, and ≥ 25%) (40), type of comparator diet (usual diet and low-fat diet), method of adherence to dietary intervention (self-reported versus prescribed), and presence or absence of effect modifiers (calorie restriction and exercise program) in the intervention program to find the source of possible heterogeneity. We assessed the subgroup differences as credible based on eight criteria introduced by the Instrument to Assess the Credibility of Effect Modification Analyses (ICEMAN) (41) (Supplementary Table S2). We ran sensitivity analyses by excluding studies one by one to evaluate the stability of the pooled results. For the analyses of adverse events and medication reduction, we calculated the risk difference and relative risk and their 95% CI using the number of participants and events in the intervention and control groups. Publication bias was examined by the visual inspection of the funnel plots and Egger’s test when at least ten primary studies were available (42). Statistical analyses were conducted using STATA version 16.1. A two-tailed *p*-value of less than 0.05 was considered significant.

### Grading the evidence

The certainty of the evidence was assessed through the Grading of Recommendations Assessment, Development, and Evaluation (GRADE) approach (43, 44). Briefly, evidence obtained from randomized trials that were initially considered as having high certainty could be downgraded by a serious risk of bias, inconsistency, indirectness, imprecision, and evidence of publication bias (Supplementary Text S2). The evidence could also be upgraded due to the large effect size and the presence of a dose–response gradient. To rate for imprecision, the minimal clinically important difference (MCID) threshold was considered to be 5% of baseline body weight (equal to 4.39 kg) (45). Overall quality assessment was conducted independently by two authors (SS and AJ). Discrepancies were resolved through discussion to reach a consensus.

## Results

### Literature search and study selection process

We initially identified 20,803 studies. After initial screening and duplicate removal, 526 records remained, of which 416 were excluded for the reasons presented in Supplementary Table S3. Finally, 110 studies were selected for the present meta-analysis. The flowchart of the study selection procedure that followed the literature search is summarized in Supplementary Figure S1.

### Risk of bias

As shown in Supplementary Table S4, 11 studies (10%) were rated with a low risk of bias, 28 (25%) were rated with a high risk of bias, and the rest of them (*n* = 72, 65%) were rated as having some concerns. In all, 37 trials did not report the random sequence generation methods, while 70 trials did not describe details of the allocation concealment. Due to the nature of the trial, a large part of the trials (approximately 70%) was rated as having unclear or high risks of bias regarding the blinding of the participants and the outcome assessors. However, as body weight was measured objectively, it is highly unlikely that this affected the findings. In total, 81 trials were rated as having a low risk of attrition bias (<20% attrition and conducted an intent-to-treat analysis), and 84 trials were rated as having a low risk of reporting bias (an approved protocol was assigned).

### Study characteristics

Of the 110 trials that met our inclusion criteria, 107 were parallel RCTs and 3 were cross-over RCTs. Trials were published between 1981 and 2021. All trials were conducted exclusively with participants who were overweight or obese, with a BMI ranging from 25 to 44.6 kg/m^2^. A total of 9 trials included only men, 24 included only women, and the remainder included both men and women. In total, 61 trials (55%) investigated the effects of a carbohydrate-restricted diet in healthy patients who were overweight or obese, and the other 49 trials (45%) were conducted in overweight or obese patients with a comorbid condition. The mean age of the participants varied between 20 and 67.8 years. Trials were conducted primarily in the US (*n* = 45), Australia/New Zealand (*n* = 20), Asia (*n* = 9), and Europe (*n* = 36). The length of the trial ranged from 4 weeks to 104 weeks. A majority of the trials (*n* = 108) had a two-arm design (intervention and control), and two trials had four study arms. Approximately 30% of the trials (*n* = 31) had isocaloric intervention and control arms, 60 trials (55%) administered calorie restriction in both intervention and control arms, 6 trials implemented calorie restriction only in the intervention (carbohydrate-restricted) arm, and 13 trials implemented calorie restriction only in the control (high carbohydrate) diet. One-third of the trials (*n* = 33) implemented a combination of an exercise program and a carbohydrate-restricted diet for their participants. In total, 18 trials (16%) implemented a very low-carbohydrate (ketogenic) diet (≤10% of total calories or 20–50 g/day), 19 trials (17%) implemented a low-carbohydrate diet (<26% of total calories or 50–130 g/day), and 73 trials (67%) followed a moderate-carbohydrate diet (26–45% of the total calories or 130–230 g/day). Total protein intake ranged from 15 to 55% of calorie intake (median = 27%) in the intervention groups and from 10.4 to 45% of calorie intake (median = 18.65%) in the control groups. Furthermore, 85% (*n* = 94) of the trials in the carbohydrate-restricted group and 21% of the trials (*n* = 23) in the high-carbohydrate group prescribed a diet rich in protein (>20%). An overview of the study characteristics is presented in Supplementary Table S5, and the macronutrient composition of the diets across study arms in each trial is presented in Supplementary Table S6.

### Main analysis

**Figure 1:**
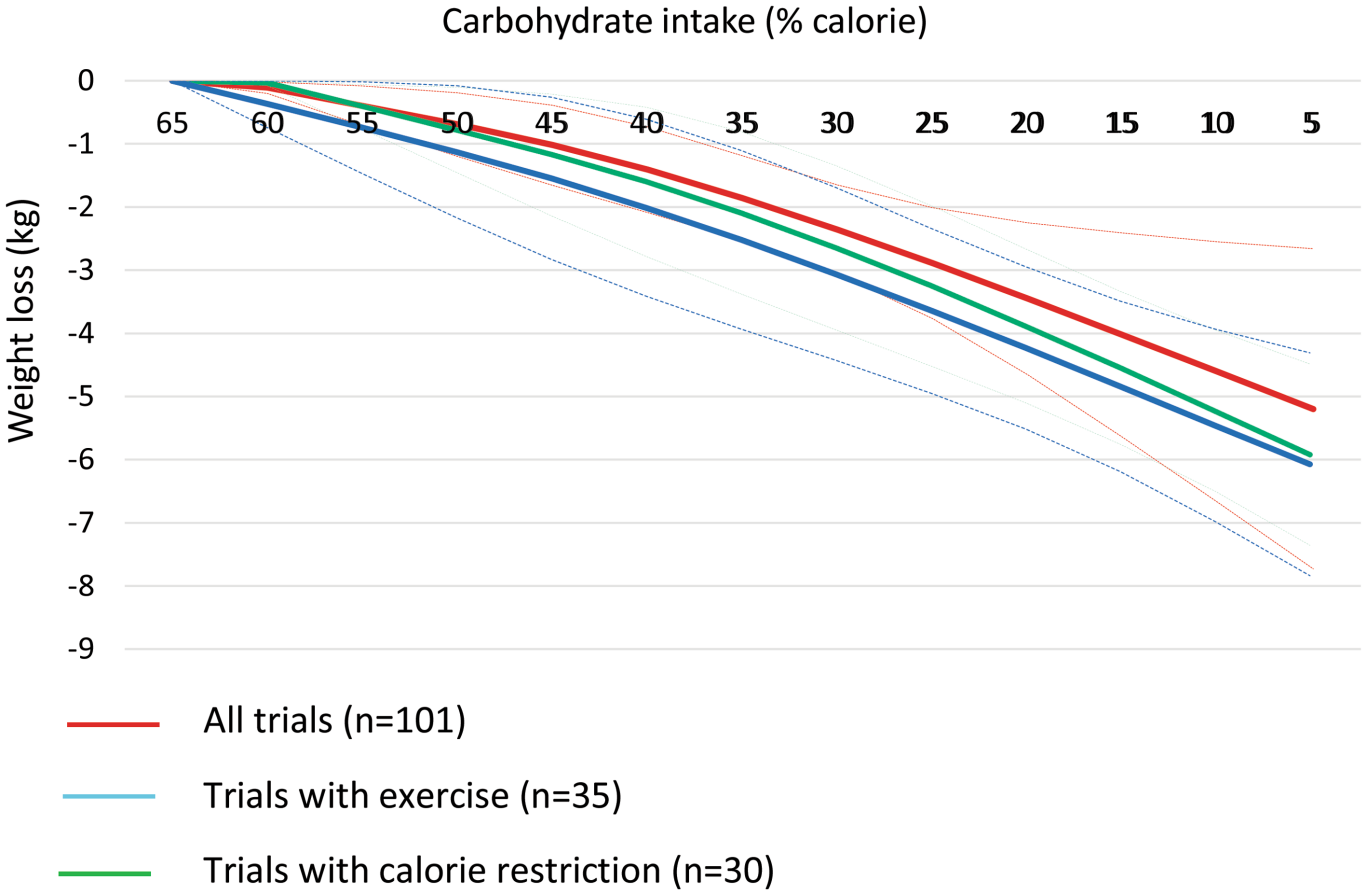
Dose-dependent effect of change in body weight according to carbohydrate restriction in overweight and obese participants at 6-month follow-up (red line), trials with calorie restriction (green line), and trials with an exercise program (blue line). Changes in the body weight (y-axis) are presented as means (kg). Carbohydrate restriction dose (x-axis) is presented as a reduction in carbohydrate intake (% of calorie). The central straight line (black line) represents the fitted dose–response estimate with outer dashed lines representing the 95% confidence intervals (CIs) (gray line), which was modeled using one-stage random effects with the generic inverse variance and restricted maximum likelihood methods, assuming a linear function.

At the 6-month follow-up, linear dose–response meta-analysis indicated that each 10% decrease in carbohydrate intake reduced body weight by 0.64 kg (95% CI, −0.79 to −0.49, I^2^ = 80.5%; *n* = 101 trials with 4,135 participants, GRADE = high certainty) (the figure was not shown due to a large number of trials). In the non-linear dose–response meta-analysis, there was a monotonic reduction in body weight with a decrease in carbohydrate intake from 65 to 5%, with the greatest reduction at 5% (mean difference 5%: −3.96 kg, 95% CI: −4.92 to −3.00) (Figure 1 and Table 1). The effect of carbohydrate-restricted diets on weight loss did not surpass the threshold set as MCID in the main analysis; however, it did exceed that threshold (4.39 kg) when coupled with calorie restriction or an exercise program (Table 1).

**Table 1:** Effects of carbohydrate restriction on body weight at 6-month follow-up from the non-linear dose–response meta-analysis (mean difference and 95% confidence interval).

Carbohydrate intake (% calorie)	Overall (*n* = 101)	Trials with calorie restriction (*n* = 35)	Trials with exercise program (*n* = 30)
65.5% (Ref)	0	0	0
60%	−0.02 (−0.05, −0.001)	−0.28 (−0.57, −0.00)	−0.11 (−0.20, −0.02)
55%	−0.27 (−0.51, −0.04)	−0.57 (−1.13, −0.01)	−0.40 (−0.71, −0.08)
50%	−0.52 (−0.97, −0.07)	−0.87 (−1.67, −0.06)	−0.69 (−1.20, −0.19)
45%	−0.78 (−1.43, −0.14)	−1.19 (−2.18, −0.20)	−1.02 (−1.66, −0.39)
40%	−1.07 (−1.86, −0.28)	−1.55 (−2.63, −0.47)	−1.41 (−2.09, −0.73)
35%	−1.40 (−2.26, −0.54)	−1.94 (−3.03, −0.85)	−1.86 (−2.53, −1.19)
30%	−1.77 (−2.64, −0.90)	−2.36 (−3.41, −1.30)	−2.36 (−3.06, −1.65)
25%	−2.17 (−3.02, −1.32)	−2.80 (−3.81, −1.80)	−2.89 (−3.77, −2.01)
20%	−2.60 (−3.41, −1.78)	−3.26 (−4.25, −2.27)	−3.45 (−4.66, −2.25)
15%	−3.04 (−3.85, −2.23)	−3.73 (−4.77, −2.69)	−4.03 (−5.65, −2.41)
10%	−3.50 (−4.35, −2.64)	−4.21 (−5.38, −3.03)	−4.61 (−6.68, −2.55)
5%	−3.96 (−4.92, −3.00)	−4.68 (−6.04, −3.32)	−5.20 (−7.73, −2.66)

**Figure 2:**
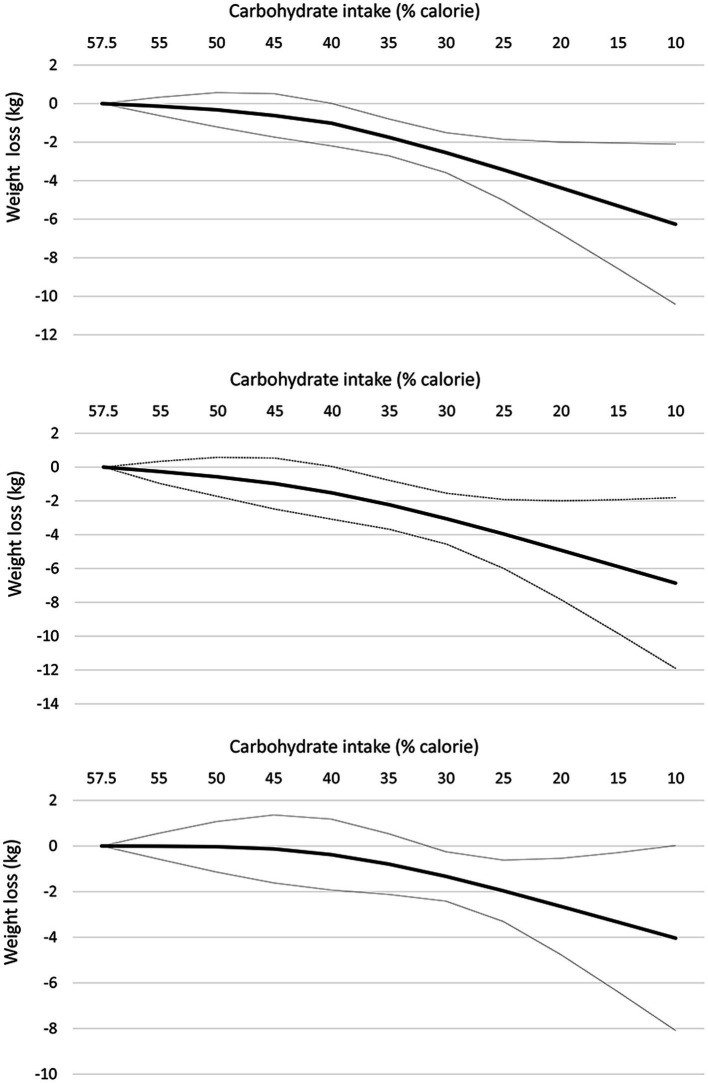
Dose-dependent effect of change in body weight according to carbohydrate restriction in overweight and obese participants at 12-month follow-up (upper panel), trials with calorie restriction (middle panel), and trials with an exercise program (lower panel). Changes in the body weight (y-axis) are presented as means (kg). Carbohydrate restriction dose (x-axis) is presented as a reduction in carbohydrate intake (% of calories). The central straight line (black line) represents the fitted dose–response estimate with outer dashed lines representing the 95% confidence intervals (CIs) (gray line), which was modeled using one-stage random effects with the generic inverse variance and restricted maximum likelihood methods, assuming a linear function.

At the 12-month follow-up, linear dose–response meta-analysis indicated that each 10% decrease in carbohydrate intake reduced body weight by 1.15 kg (95% CI: −1.61 to −0.69, I^2^ = 93%; *n* = 42 trials with 2,657 participants; GRADE = moderate certainty, Supplementary Figure S2). There was a non-linear reduction in body weight with the decrease in carbohydrate intake from 57.5 to 10%, with the greatest reduction at 10% (mean difference 10%: −6.26 kg, 95% CI: −10.42 to −2.10) (Table 2 and Figure 2, upper panel). The results in the subgroup of trials with calorie restriction (Figure 2, middle panel) and exercise program (Figure 2, lower panel) indicated relatively similar findings with the main analysis.

**Table 2:** Effects of carbohydrate restriction on body weight at 12-month follow-up from the non-linear dose–response meta-analysis (mean difference and 95% confidence interval).

Carbohydrate intake (% calorie)	Overall (*n* = 42)	Trials with calorie restriction (*n* = 29)	Trials with exercise programs (*n* = 17)
57.5% (Ref)	0	0	0
55%	−0.14 (−0.62, 0.33)	−0.27 (−0.98, 0.34)	−0.01 (−0.58, 0.56)
50%	−0.32 (−1.21, 0.57)	−0.58 (−1.74, 0.58)	−0.03 (−1.14, 1.07)
45%	−0.62 (−1.74, 0.51)	−0.98 (−2.49. 0.53)	−0.13 (−1.62, 1.36)
40%	−1.09 (−2.20, 0.01)	−1.53 (−3.09, 0.03)	−0.38 (−1.93, 1.18)
35%	−1.75 (−2.71, −0.80)	−2.23 (−3.68, −0.79)	−0.79 (−2.12, 0.54)
30%	−2.55 (−3.59, −1.51)	−3.06 (−4.56, −1.55)	−1.33 (−2.42, −0.25)
25%	−3.44 (−5.03, −1.85)	−3.96 (−6.00, −1.92)	−1.97 (−3.31, −0.62)
20%	−4.37 (−6.76, −1.99)	−4.92 (−7.84, −1.99)	−2.65 (−4.76, −0.54)
15%	−5.31 (−8.57, −2.05)	−5.89 (−9.84, −1.93)	−3.34 (−6.40, −0.29)
10%	−6.26 (−10.42, −2.10)	−6.86 (−11.90, −1.81)	−4.04 (−8.09, 0.02)

**Figure 3:**
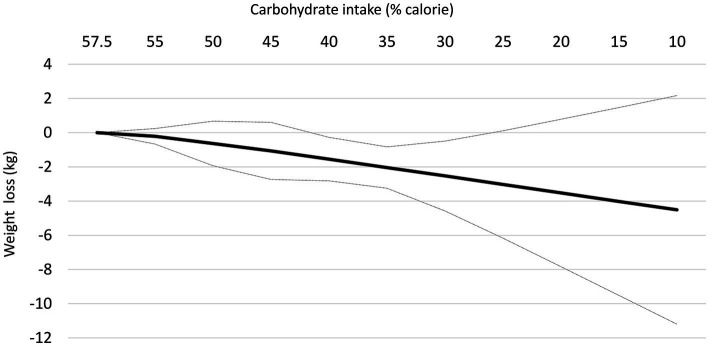
Dose-dependent effect of change in body weight according to carbohydrate restriction in overweight and obese participants at follow-up longer than 12-month follow-up. Changes in the body weight (y-axis) are presented as means (kg). Carbohydrate restriction dose (x-axis) is presented as a reduction in carbohydrate intake (% of calories). The central straight line (black line) represents the fitted dose–response estimate with outer dashed lines representing the 95% confidence intervals (CIs) (gray line), which was modeled using one-stage random effects with the generic inverse variance and restricted maximum likelihood methods, assuming a linear function.

At follow-up longer than 12 months, the reduction in body weight following each 10% decrease in carbohydrate intake was not significant in the linear dose–response meta-analysis (mean difference: −0.87 kg; 95% CI: −1.81, 0.08, I^2 =^92%; *n* = 9 trials with 1,222 participants, GRADE = low certainty) (Supplementary Figure S3). Despite a non-significant effect in the main analysis, the non-linear dose–response meta-analysis showed a non-linear effect, with significant weight loss starting at 40% (mean difference 40%: −1.55 kg, 95% CI: −2.82 to −0.27) and with the greatest reduction being observed at 30% (mean difference: −2.53 kg, 95% CI: −4.58 to −0.49) (Table 3 and Figure 3). Of note, carbohydrate intake greater than 40% and lower than 30% did not result in a significant weight loss at follow-up longer than 12 months.

**Table 3:** Effects of carbohydrate restriction on body weight for longer than 1-year follow-up from the non-linear dose–response meta-analysis (mean difference and 95% confidence interval; *n* = 9 trials).

Carbohydrate intake (% calorie)	Mean difference (95% CI)
57.5% (Ref)	0
55%	−0.21 (−0.66, 0.24)
50%	−0.63 (−1.93, 0.67)
45%	−1.07 (−2.74, 0.60)
40%	−1.55 (−2.82, −0.27)
35%	−2.04 (−3.25, −0.83)
30%	−2.53 (−4.58, −0.49)
25%	−3.03 (−6.17, 0.11)
20%	−3.52 (−7.83, 0.78)
15%	−4.02 (−9.51, 1.47)
10%	−4.51 (−11.20, 2.17)

### Subgroup analysis

At the 6-month follow-up, the results were the same across subgroups defined by risk of bias (Supplementary Table S7). Although point estimates were relatively larger in the subgroups of the trials that implemented calorie restriction (mean difference for each 10% decrease: -0.80, 95% CI: −1.08 to −0.51; *n* = 35) and physical activity (mean difference for each 10% decrease: -0.84, 95% CI: −1.12 to −0.56; *n* = 30) as compared to trials that did not (Supplementary Table S7), the differences between subgroups were not statistically significant and the credibility of subgroup differences was rated low (Supplementary Table S8). The effect of carbohydrate restriction on body weight was stronger in trials that implemented moderate protein (20–25% protein; mean difference for each 10% decrease: −0.57 kg, 95% CI: −0.79 to −0.35; *n* = 22) and high protein (≥25% protein; mean difference for each 10% decrease: −0.72 kg, 95% CI: −0.90 to −0.53; *n* = 64) diets as compared to low protein diets (<20% protein; mean difference for each 10% decrease: −0.23 kg, 95% CI: −0.74 to 0.29; *n* = 16), but, again, there was not a statistically significant difference across subgroups and the credibility of the subgroup difference was rated low. The effect was significantly stronger in participants who were unhealthy (mean difference: −1.00 kg, 95% CI: −1.31 to −0.68; *n* = 40) as compared to otherwise healthy participants (mean difference: −0.46 kg, 95% CI: −0.62 to −0.29; *n* = 61) (P for subgroup difference = 0.003, ICEMAN credibility = low) (Supplementary Table S7). There was no credible subgroup difference in the subgroup analyses based on the eight criteria introduced by ICEMAN (Supplementary Table S8).

At the 12-month follow-up, there was a significant subgroup difference, where trials that implemented exercise programs indicated a stronger effect (mean difference for each 10% decrease: −1.56 kg, 95% CI: −2.27 to −0.85; *n* = 25) than those that did not (mean difference for each 10% decrease: −0.63 kg, −1.09 to −0.17; *n* = 17) (P for subgroup difference = 0.03, ICEMAN credibility = low) (Supplementary Tables S9, S10). The results were the same in trials that implemented calorie restriction (mean difference for each 10% decrease: −1.25 kg, 95% CI: −1.79 to −0.71; *n* = 35) and in those that did not (mean difference for each 10% decrease: −0.86 kg, 95% CI: −1.93 to 0.21; *n* = 13) (P for subgroup difference = 0.52, ICEMAN credibility = low). The effect was stronger in the subgroup of trials that implemented moderate protein (20–25% protein; mean difference for each 10% decrease: -1.06, 95% CI: −2.08 to −0.04; *n* = 10) and high protein (≥25% protein; mean difference for each 10% decrease: -1.47, 95% CI: −2.16 to −0.79; *n* = 24) diets as compared to low protein diets (<20% protein; mean difference for each 10% decrease: -0.27, 95% CI: −1.10 to 0.57; *n* = 8), but the credibility of the subgroup difference was rated low (P for subgroup difference = 0.09). There was no significant or credible subgroup difference at follow-up longer than 12 months (Supplementary Tables S11, S12).

### Publication bias

The shape of funnel plots and Egger’s tests did not reveal any signs of publication bias for body weight reduction across time periods (Supplementary Figure S4).

### Grading the evidence

The results of the GRADE assessment are indicated in Supplementary Table S13. The certainty of the evidence was rated high at the 6-month follow-up due to a downgrade for inconsistency and an upgrade for dose–response gradient. The certainty of the evidence was rated moderate at the 12-month follow-up and very low at follow-up longer than 12 months. At the 6-month follow-up, the effects of carbohydrate restriction did not exceed the MCID threshold for body weight (4.39 kg) in the main analysis; however, they did surpass the MCID threshold when coupled with calorie restriction and an exercise program (Table 1). At the 12-month follow-up, carbohydrate restriction resulted in a clinically important reduction in body weight, larger than the MCID threshold, in the main analysis and in trials that implemented calorie restriction (Table 2). At the follow-up longer than 12 months, carbohydrate restriction significantly reduced body weight, but the magnitude of the effect was smaller than the MCID threshold (Table 3).

### Adverse events

The reported adverse effects related to a carbohydrate-restricted diet are shown in Table 4. At 6-month follow-up, carbohydrate restriction significantly increased hair loss by 16 per 100 patients (risk difference: 0.16, 95% CI 0.02 to 0.31; relative risk: 2.07, 95% CI: 1.31 to 3.28), and muscle cramp by 20 per 100 patients (risk difference: 0.20, 95% CI 0.10 to 0.29; relative risk: 4.88, 95% CI: 1.65 to 14.40). Carbohydrate restriction had no effect on other adverse events.

**Table 4:** Adverse effect of carbohydrate-restricted diet.

Side effect	Number of trials	Risk difference (95% CI)	Relative risk (95% CI)
Hair loss (at 6-month follow-up)	3	0.16 (0.02, 0.31)	2.07 (1.31, 3.28)
Constipation (at 6-month follow-up)	5	0.09 (−0.01, 0.19)	1.24 (0.79, 1.95)
Fatigue (at 6-month follow-up)	3	0.05 (−0.06, 0.17)	1.35 (0.55, 3.34)
Diarrhea (at 6-month follow-up)	3	0.04 (−0.05, 0.13)	1.51 (0.63, 3.62)
Nausea (at 6-month follow-up)	4	0.01 (−0.03, 0.06)	1.35 (0.31, 5.87)
Headache (at 6-month follow-up)	4	0.01 (−0.03, 0.06)	1.35 (0.33, 3.34)
Muscle cramps (at 6-month follow-up)	3	0.20 (0.10, 0.29)	4.88 (1.65, 14.40)
Constipation (at 12-month follow-up)	4	0.13 (−0.06, 0.34)	1.30 (0.89, 1.90)

## Discussion

The present dose–response meta-analysis suggests that a reduction in carbohydrate intake can be effective in producing clinically important weight loss at short- and moderate-term follow-ups. Non-linear dose–response meta-analyses indicated that body weight decreased proportionally along with the decrease in carbohydrate intake at 6-month and 12-month follow-ups, especially when coupled with an exercise program and calorie restriction. At the follow-up longer than 12 months, carbohydrate intake between 30 and 40% of calorie intake resulted in a significant reduction in body weight; however, carbohydrate intake <30 and > 40% did not significantly reduce body weight at follow-up longer than 12 months.

Dietary strategies to lose weight have predominantly focused on calorie restriction, mainly through the restriction of dietary carbohydrates or fats (46). In line with our findings, most of the previous studies agree that in the short term, low-carbohydrate diets are more effective in losing weight, while in the long term, there is not much difference between weight-loss diets. Moreover, a recent network meta-analysis of 121 randomized trials with 21,942 participants compared the weight-loss effects of 14 structured dietary programs and revealed that low-carbohydrate diets are the most effective plans for losing weight at 6 months (26). The study also found that the weight-loss effects of all dietary programs diminished markedly at follow-ups longer than 12 months. Another meta-analysis of five randomized trials with 447 patients indicated a significant weight loss following a carbohydrate-restricted diet at the 6-month follow-up, but not at 12-month follow-up (19). Furthermore, a meta-analysis of 13 trials found a 4.02 kg weight reduction in favor of carbohydrate-restricted dietary interventions at 6-month follow-up and 1.05 kg at 12-month follow-up (47).

The reduced effect of low-carbohydrate diets on losing weight in the long term is mainly associated with reduced adherence (48). Moreover, the craving for sweet tastes is removed in low-carbohydrate diets, the pleasure of eating is reduced, and dietary choices are more limited in carbohydrate-restricted diets, all of which make these diets more difficult to maintain in the long term (49). Focus on the short-term can lead to treatment failure and to weight being regained, which can result in demotivation and decreased self-esteem. Furthermore, the physiological responses to losing weight are another causative factor in weight plateau and weight regain in long-term weight management plans. Behavioral interventions for obesity treatment, for instance, calorie restriction, lead to an initial rapid weight reduction, followed by slowing down of weight loss and even weight regain to the baseline due to the decreased body metabolism and energy expenditure (50). However, low-carbohydrate diets are still popular for losing weight. This effect is mostly explained through the carbohydrate–insulin model, suggesting hyperinsulinemia after high-carbohydrate food consumption, which, in turn, increases fat storage and body weight (51). Moreover, the production of ketone bodies in response to carbohydrate deprivation can suppress appetite and decrease food intake (52). Other related mechanisms including increased energy expenditure and decreased ghrelin and leptin levels following carbohydrate restriction may partly mediate the weight-loss effects of carbohydrate-restricted diets (51).

Our dose–response meta-analyses indicated a proportional reduction in body weight with the decrease in carbohydrate intake, with the greatest reduction in body weight at 5–10% carbohydrate intake, suggesting that very-low-carbohydrate (ketogenic) diets are the most effective diets among different types of carbohydrate-restricted diets for weight loss. This effect may be enhanced when carbohydrate restriction is accompanied by calorie restriction or an exercise program. Moreover, we found significant weight loss at follow-ups longer than 12 months only with carbohydrate intake between 30 and 40%, and that carbohydrate intake below this range was no longer effective for weight loss in the long term.

The subgroup analyses suggested that the weight-reducing effects of carbohydrate-restricted diets increased when coupled with calorie restriction and exercise programs; however, the observed subgroup differences were not statistically significant, and the credibility of subgroup differences was rated as low, suggesting that carbohydrate restriction, regardless of calorie restriction and exercise program, can exert a significant reduction in body weight. In addition, a subgroup analysis by the proportion of protein intake in the intervention arms suggested that high (≥25% of calorie intake) and moderate (20–25% of calorie intake) protein diets were more effective for weight loss as compared to low protein diets (<20% of total calorie intake) at both short- and long-term follow-up; however, the observed differences between subgroups were not statistically significant.

Our results suggest to clinicians that if the goal is to achieve weight loss in the short term, for example, if the patient needs to lose weight to be ready for surgery or if obesity is associated with serious complications, a low-carbohydrate diet with 5–10% carbohydrate could produce a greater weight-loss effect, and it is recommended to keep protein at 20–25% of calorie intake. For long-term goals, there is no priority in different weight-loss diets based on the previous studies, but our study suggests optimum carbohydrate intake for a low-carbohydrate weight-loss diet could be between 30 and 40% of total calorie intake. Although there are a few concerns in relation to low-carbohydrate diets, they are related to the potential side effects. There is some evidence that carbohydrate-restricted diets, which are also high in protein or fat, can lead to nutritional deficiency and dysbiosis (53), gastrointestinal complications (54), hyperuricemia (55), renal impairment (56), osteoporosis (54), and increased blood cholesterol (12). However, there is no firm evidence indicating an increased risk of nutrient deficiency following a low-carbohydrate diet. Our study also suggested that the adverse effects of low-carbohydrate diets were generally mild to moderate, including hair loss and muscle cramps.

There are several meta-analyses of randomized trials addressing the weight-loss effects of carbohydrate-restricted diets (12–22); However, none of the previous reviews investigated how much carbohydrate restriction has greater advantages for weight loss in adults with overweight or obesity. However, the present study provided new insights into this topic. An important and unanswered question regarding the effect of carbohydrate-restricted diets on body weight was how much carbohydrate intake has the greatest effect on weight loss and whether this effect lasts for a long time. To answer this question, we used a novel statistical approach to determine how well body weight changes along with the decrease in carbohydrate intake. Our dose–response meta-analysis indicated that body weight decreased proportionally along with the decrease in carbohydrate intake. In contrast to previous meta-analyses, which demonstrated that carbohydrate-restricted diets were not effective for weight loss in the long term, we indicated that the weight-loss effects of carbohydrate-restricted diets in the long term depend on the degree of carbohydrate restriction, with carbohydrate intake higher than 40% and lower than 30% not being effective for weight loss.

However, some limitations should be addressed when interpreting the results. The main limitation of the present study is related to the large heterogeneity in the data, which remained unexplained in the subgroup analyses. It seems that the observed heterogeneity can be explained by potential differences in the type of carbohydrate intake (simple or complex), medications, degree of physical activity, and calorie intake across trials. We included a large number of trials in the analyses, and in such cases, even a small difference in effect estimates can lead to a large heterogeneity in the data (57). In addition, all trials included in the present meta-analysis were consistent in terms of the PICOS (population, intervention or exposure, comparator, outcome, and study design) framework (58). Therefore, the heterogeneity observed in the data was statistical heterogeneity rather than clinical or methodological heterogeneity. Second, we did not evaluate the effect of carbohydrate restriction on other anthropometric measures. Examining other outcomes, such as body composition measures, can also be helpful in interpreting the results. Although it is reported that intentional weight reduction is associated with improvement in body fat mass, investigating the effects of carbohydrate restriction on lean body mass can provide stronger evidence to assess the effectiveness of the intervention (14, 59). Moreover, due to inadequate information, we could not examine the effect of dietary intervention on the grade of obesity. The degree of calorie restriction varied from less than 800 to 1,500 kcal per day across trials. Although we tried to assess the effect of calorie restriction using subgroup and sensitivity analyses, the difference in the degree of calorie restriction may also affect the results. We also performed a subgroup analysis by control diet; however, the potential difference in the control diets across trials may have affected the results. Another important limitation is related to the source of macronutrients. We assessed the effect of the quantity of carbohydrate intake, and thus, due to the inadequate data, the type of carbohydrate was not considered in the analyses. Moreover, the quality of the diet in the intervention arm, dietary sources of protein or fats, and micronutrients may also affect body weight, which was not considered in most of the included studies. Finally, there is a need for trials with a longer duration of intervention to assess the effect of dietary composition on body weight and other health-related outcomes.

## Conclusion

The present study showed that at 6- and 12-month follow-ups, body weight decreased proportionally with the decrease in carbohydrate intake. The greatest reduction was seen at 5 and 10% carbohydrate intake at 6-month and 12-month follow-ups, respectively, suggesting that very-low-carbohydrate (ketogenic) diets may be the best approach among carbohydrate-restricted diets for weight loss. Carbohydrate-restricted diet exerted a significant and clinically important reduction in body weight at both 6-month and 12-month endpoints, especially when coupled with calorie restriction and physical activity. At follow-up longer than 12 months, the optimum carbohydrate intake for weight loss was between 30 and 40%, with the greatest reduction at 30%. Further studies are needed to explore the effect of other macronutrient intakes and eating behaviors on the efficacy of low-carbohydrate diets.

## Data availability statement

The original contributions presented in the study are included in the article/Supplementary material, further inquiries can be directed to the corresponding author.

## Author contributions

SS: Writing – original draft, Writing – review & editing, Formal analysis. AJ: Conceptualization, Writing – original draft, Writing – review & editing. SA: Writing – review & editing. AV: Writing – review & editing. FM: Writing – review & editing. SS-B: Conceptualization, Writing – review & editing.
