# Supplementary material for: Effect of carbohydrate restriction on body weight in overweight and obese adults: a systematic review and dose–response meta-analysis of 110 randomized controlled trials
Source: Front Nutr. 2023 Dec 6;10:1287987. doi: 10.3389/fnut.2023.1287987 (PMC10731359; doi:10.3389/fnut.2023.1287987)
Supplement: Supplementary file 1 [file Data_Sheet_1.docx]

**Effect of carbohydrate restriction on body weight in adults with overweight and obesity: a systematic review and dose-response meta-analysis of 110 randomized controlled trials**

Sepideh Soltani^1^, Ahmad Jayedi^2^, Shima Abdollahi^3^, Azam Ahmadi Vasmehjani^4^, [Fatemeh Meshkini](https://onlinelibrary.wiley.com/action/doSearch?ContribAuthorRaw=Meshkini%2C+Fatemeh)^5^, Sakineh Shab-Bidar^6*^

*^1^ Yazd Cardiovascular Research Center, Non-communicable Diseases Research Institute, Shahid Sadoughi University of Medical Sciences, Yazd, Iran*

*^2^ Social Determinants of Health Research Center, Semnan University of Medical Sciences, Semnan, Iran*

*^3^ Department of Nutrition, School of Health, North Khorasan University of Medical Sciences, Bojnurd, Iran*

*^4^ Department of Nutrition, School of Public Health, Shahid Sadoughi University of Medical Sciences, Yazd, Iran*

*^5^ Department of Biochemistry, School of medicine, Shahid Sadoughi University of Medical Sciences, Yazd, Iran*

*^6^ Department of Community Nutrition, School of Nutritional Sciences and Dietetics, Tehran University of Medical Sciences, Tehran, Iran*

**Running title:** Carbohydrate restriction and body weight

***Correspondence to:** Sakineh Shab-Bidar**, Associate Professor, Department of Community Nutrition, School of Nutritional Sciences and Dietetics, Tehran University of Medical Sciences, P. O. Box 14155/6117, Tehran, Iran Telefax: +98(21)88955979, Email:** [s_shabbidar@tums.ac.ir](mailto:s_shabbidar@tums.ac.ir), ORCID [0000-0002-0167-7174](https://orcid.org/0000-0002-0167-7174), Web of Science ResearcherID: [H-9525-2017](https://publons.com/researcher/H-9525-2017/)

| **Content** | **Caption** | **Page** |
| --- | --- | --- |
| **Table S1** | Literature search strategy | 3 |
| **Text S1** | Reasons for risk of bias assessment judgement in the present systematic review | 4 |
| **Text S2** | Instructions to rate the certainty of evidence using the GRADE approach. | 5 |
| **Table S2** | Description of the ICEMAN domains and how to judge each domain. | 6 |
| **Table S3** | Lists of studies excluded via full-text assessment and reasons for exclusions. | 7 |
| **Table S4** | Study quality and risk of bias assessment of included studies according to the Cochrane tool | 9 |
| **Table S5** | Characteristics of trials that investigated the effect of low carbohydrate diet on body weight(kg) in overweight and obese adults | 13 |
| **Table S6** | The amount of prescribed/ consumed dietary macronutrient (% per calorie) in low and high carbohydrate diet | 19 |
| Table S7 | Subgroup analysis of the effect of carbohydrate restriction (10%calorie) on body weight (kg) at 6-months follow-up | 26 |
| Table S8 | Assessment of credibility of subgroup difference for the effect of carbohydrate restriction (10%calorie) on body weight (kg) at 6-months follow-up based on ICEMAN. | 27 |
| Table S9 | Subgroup analysis of the effect of carbohydrate restriction (10%calorie) on body weight (kg) at 12-months follow-up | 28 |
| Table S10 | Assessment of credibility of subgroup difference for the effect of carbohydrate restriction (10%calorie) on body weight (kg) at 12-months follow-up based on ICEMAN. | 29 |
| Table S11 | Subgroup analysis of the effect of carbohydrate restriction (10%calorie) on body weight (kg) for longer than 12 months follow-up | 30 |
| Table S12 | Assessment of credibility of subgroup difference for the effect of carbohydrate restriction (10%calorie) on body weight (kg) for longer than 12 months follow-up based on ICEMAN. | 31 |
| Table S13 | GRADE evidence for the effect of carbohydrate restriction on body weight at 6-month, 12-month, and longer than 1 year follow-up | 32 |
| **Fig S1** | Flow diagram of the study selection process. | 33 |
| **Fig S2** | The effect of carbohydrate restriction (10%calorie) on body weight (kg) at 12-months follow-up | 34 |
| **Fig S3** | The effect of carbohydrate restriction (10%calorie) on body weight (kg) for longer than 12 months follow-up | 35 |
| **Fig S4** | Funnel plot of the effect of trials on body weight (kg) pressure at 6-month and 12- month follow-up | 36 |
| **References** | References | 37 |

| **Supplementary Table1**- Literature search strategy. |
| --- |
| #1 “Low-carbohydrate” [Title/Abstract] OR Ketogenic [Title/Abstract] OR “Low carbohydrate” [Title/Abstract] OR “high fat” [Title/Abstract] OR “high-fat” [Title/Abstract] OR “VLCKD” [Title/Abstract] OR "Diet, Carbohydrate-Restricted"[Mesh] OR "Carbohydrate-Restricted"[Title/Abstract] OR "Carbohydrate Restricted"[Title/Abstract] OR "Carbohydrates-Restricted"[Title/Abstract] OR "Carbohydrates Restricted"[Title/Abstract] OR “Carbohydrate Restrict*”[Title/Abstract] OR “Carbohydrates Restrict*”[Title/Abstract] OR "carbohydrate free"[Title/Abstract] OR "carbohydrates free"[Title/Abstract] OR “carbohydrate free*”[Title/Abstract] OR “carbohydrates free*”[Title/Abstract] OR "Low Carbohydrate"[Title/Abstract] OR "Low Carbohydrates"[Title/Abstract] OR “Low Carbohydrat*”[Title/Abstract] OR "South Beach Diet"[Title/Abstract] OR “Atkins Diet*”[Title/Abstract] OR “low carb*”[Title/Abstract] OR “Ornish” OR “calorie restriction” [Title/Abstract] OR “weight-loss diet*”[Title/Abstract] OR “Weight reduction diet”[Title/Abstract] OR “Weight-reduction diet” [Title/Abstract] OR “High-Protein Low Carbohydrate” [Title/Abstract] OR "Diet, Carbohydrate-Restricted"[Mesh] OR "Diet, [High-Protein](https://www.ncbi.nlm.nih.gov/mesh/2023142)” [Mesh] OR [High-Protein](https://www.ncbi.nlm.nih.gov/mesh/2023142)[Title/Abstract]#2 (Randomized [Title/Abstract] OR random[Title/Abstract] OR Intervention[Title/Abstract] OR "Clinical trial"[Title/Abstract] OR "Randomized controlled trial"[Title/Abstract] OR "Randomized controlled trials"[Title/Abstract] OR Placebo [Title/Abstract] OR "Double-blind"[Title/Abstract] OR Clinical trial [Mesh] OR "Random Allocation" [Mesh] OR "Randomized controlled trial" [TIAB] OR "Randomised controlled trial"[Title/Abstract] OR "Randomised controlled trials"[Title/Abstract] OR Randomised [Title/Abstract] OR "Randomised clinical trials"[Title/Abstract] OR "Randomised clinical trial"[Title/Abstract] OR trial [Title/Abstract])#3 weight*[All fields] OR obes*[All fields]#4 ("systematic review" [Title] OR "meta-analysis"[Title] OR review [Title/Abstract] OR review[Publication Type] OR Athletes [Title/Abstract] OR child [Title]OR adolescent* [Title] OR Mice [Title]#5 (#1 AND #2 AND #3) NOT (#4) |

| **Text S1-** Reasons for risk of bias assessment judgement in the present study |
| --- |
| 1. **Random sequence generation**   Low risk of bias: Available describe a random component in the sequence generation process  High risk of bias: Describe a non-random component in the sequence generation process  Unclear risk of bias: Not described in sufficient detail to permit judgment   1. **Allocation concealment**   Low risk of bias: Available information showed that participants and investigators enrolling participants could not foresee assignment  High risk of bias: Describe information showed that participants and investigators enrolling participants could foresee assignment  Unclear risk of bias: Not described in sufficient detail to permit judgment   1. **Blinding of participants and personnel**   Low risk of bias: Blinding of participants and key study personnel ensured, or the review authors judge that the outcome is not likely to be influenced by lack of blinding  High risk of bias: No blinding or incomplete blinding, and the outcome is likely to be influenced by lack of blinding  Unclear risk of bias: Not described in sufficient detail to permit judgment   1. **Blinding of outcome assessor**   Low risk of bias: Blinding of outcome assessment ensured, or the review authors judge that the outcome measurement is not likely to be influenced by lack of blinding  High risk of bias: No blinding or incomplete blinding, and the outcome measurement is likely to be influenced by lack of blinding  Unclear risk of bias: Not described in sufficient detail to permit judgment   1. **Incomplete outcome data**   Low risk of bias: No missing outcome data, or reasons for missing outcome data unlikely to be related to true outcome, or missing outcome data balanced in numbers across intervention and control groups, or conducted intention-to-treat analysis  High risk of bias: >20% attrition bias, and reason for missing outcome data likely to be related to outcome, either imbalance attrition in numbers or reasons for missing data across intervention and control groups Unclear risk of bias: Not described in sufficient detail to permit judgment   1. **Selective reporting**   Low risk of bias: No evidence for results selection and pre-specified trial protocol available.  High risk of bias: Presence of evidence for results selection.  Unclear risk of bias: Not described in sufficient detail to permit judgment |

| **Text S2-** Instructions to rate the certainty of evidence using the GRADE approach. |
| --- |
| The certainty of the evidence were rated using the GRADE approach (1). GRADE rates the certainty of evidence as high, moderate, low, or very low. Randomized controlled trials start as high certainty evidence that can then be downgraded based on pre-specified criteria. The criteria used to downgrade evidence include:  *Study limitations*-weight of studies showing risk of bias as assessed by the Cochrane Risk of Bias instrument (2). We rated down for imprecision if most studies were at high risk of bias. For outcomes that included trials with both high and low risk of biases, we performed subgroup analyses based on study quality. When P for subgroup difference was not significant, all trials were included in the main analysis. When P for subgroup difference was significant, only the result in the subgroup of trials with a low risk of bias was reported.  *Inconsistency*-substantial between-study heterogeneity, I^2^ ≥ 50% and P_heterogeneity_< 0.10 which remained unexplained in priori subgroup and sensitivity analyses (3).  *Indirectness-*presence of population, intervention or comparator factors that limit the generalizability of the results (4).  *Imprecision*-the 95% CI for the mean difference is wide or the point estimate and its corresponding 95%CI do not surpass the minimal clinically important difference (MCID) (5). For imprecision, we did not rate down for imprecision where the point estimate and its 95%CI for the linear and/or non-linear dose-response meta-analyses surpassed MCID threshold. Where the point estimates surpassed MCID, we rated down if the 95%CI overlapped MCID threshold. We did not rate down for imprecision if point estimate and its 95%CI surpassed MCID thresholds at any specific dose of intervention in the non-linear dose-response meta-analysis.  *Publication bias*-compelling evidence of publication bias (6).  We also upgraded evidence as a result of the presence of a dose response and large effect size. |

| **Table S2-** Description of the ICEMAN domains and how to judge each domain. | | | |
| --- | --- | --- | --- |
| **1: Is the analysis of effect modification based on comparison within rather than between trials?** | | | |
| Completely between | Mostly between or unclear | Mostly within | Completely within |
| Subgroup analysis or meta-regression comparing overall effects of each individual trial. This is typical for aggregate data meta-analysis. | Subgroup analysis or meta-regression with most information coming from overall effects, but some trials providing within-trial subgroup information | Most trials providing within-trial subgroup information; or individual participant data analysis that combines within and between trial information | All trials providing within-trial subgroup information or individual participant data; and the analysis separates within from between trial information, e.g. meta-analysis of interactions |
| **2: For within-trial comparisons, is the effect modification similar from trial to trial?** Not applicable: no or one within-RCT comparison | | | |
| Definitely not similar | Probably not similar or unclear | Mostly similar | Definitely similar |
| Effect modification reported for two or more trials and clearly different directions | Effect modification not reported for individual trials or too imprecise to tell | Effect modification reported for two or more trials, mostly similar in direction, but considerable differences in magnitude | Effect modification reported for two or more trials, similar in direction, only some differences in magnitude |
| **3: For between-trial comparisons, is the number of trials large?** [ ] Not applicable: no between RCT comparison | | | |
| Very small | Rather small or unclear | Rather large | Large |
| 1 or 2 or in smallest subgroup; 5 or less in continuous meta-regression | 3-4 in smallest subgroup; 6-10 in continuous meta-regression | 5-9 in smallest subgroup; 11 to 15in continuous meta-regression | 10 or more in smallest subgroup; more than 15 in continuous meta-regression |
| **4: Was the direction of effect modification correctly hypothesized a priori?** | | | |
| Definitely no | Probably no or unclear | Probably yes | Definitely yes |
| Clearly post-hoc or results inconsistent with hypothesized direction or biologically very implausible | Vague hypothesis or hypothesized direction unclear | No prior protocol available but unequivocal statement of a priori hypothesis with correct direction of effect modification | Prior protocol available and includes correct specification of direction of effect modification, e.g. based on a biologic rationale |
| **5: Does a test for interaction suggest that chance is an unlikely explanation of the apparent effect modification?** (consider irrespective of number of effect modifiers) | | | |
| Chance a very likely explanation | Chance a likely explanation or unclear | Chance may not explain | Chance an unlikely explanation |
| Interaction or meta-regression p-value >0.05 | Interaction or meta-regression p-value ≤0.05 and >0.01, or no test of interaction reported and not computable | Interaction or meta-regression p-value ≤0.01 and >0.005 | Interaction or meta-regression p-value ≤0.005 |
| **6: Did the authors test only a small number of effect modifiers or consider the number in their statistical analysis?** | | | |
| Definitely no | Probably no or unclear | Probably yes | Definitely yes |
| Explicitly exploratory analysis or large number of effect modifiers tested (e.g. greater than 10) and multiplicity not considered in analysis | No mention of number or 4-10 effect modifiers tested and number not considered in analysis | No protocol available but unequivocal statement of 3 or fewer effect modifiers tested | Protocol available and 3 or fewer effect modifiers tested or number considered in analysis |
| **7: Did the authors use a random effects model?** | | | |
| Definitely no | Probably no or unclear | Probably yes | Definitely yes |
| Fixed (or common) effect or fixed effects model explicitly stated | Probably fixed effect(s) model | Probably random (or mixed) effects | Random (or mixed) effects explicitly stated |
| **8: If the effect modifier is a continuous variable, were arbitrary cut points avoided?** [ ] not applicable: not continuous | | | |
| Definitely no | Probably no or unclear | Probably yes | Definitely yes |
| Analysis based on exploratory cut point(s), e.g. picking cut point associated with highest interaction p-value | Analysis based on cut point(s) of unclear origin | Analysis based on pre-specified cut point(s), e.g. suggested by prior RCT | Analysis based on the full continuum, e.g. assuming a linear or logarithmic relationship |
| **9 Optional: Are there any additional considerations that may increase or decrease credibility?** [ ] not applicable | | | |
| yes, probably decrease | | yes, probably increase | |
| **10. How would you rate the overall credibility of the proposed effect modification?**  Overall rating: The overall rating should be derived by the items that decrease credibility:  **Very low**: All responses definitely or probably decrease credibility or unclear  **Maximum usually low**: Two or more responses definitely decrease credibility even if all other responses satisfy credibility criteria  **Maximum usually moderate**: One response definitely decreases credibility even if all other responses satisfy credibility criteria  **Maximum usually moderate**: Two responses probably decrease credibility even if all other responses satisfy credibility criteria  **High very likely**: No response options definitely or probably decrease credibility | | | |
| **11. How would you interpreted the overall credibility of the proposed effect modification?**  **Very low**: Very likely no effect modification. Use overall effect for each subgroup.  **Maximum usually low**: Likely no effect modification. Use overall effect for each subgroup but note remaining uncertainty.  **Maximum usually moderate**: Likely effect modification. Use separate effect for each subgroup but note remaining uncertainty.  **High very likely**: Very likely effect modification. Use separate effect for each subgroup. | | | |
| **Abbreviations**: RCT, randomised controlled trials. | | | |

| **Table S3.** Lists of studies excluded via full-text assessment and reasons for exclusions. |
| --- |
| Multicomponent intervention (n=11) (7-17) |
| Did not report the amount of carbohydrate (n=16) (18-33) |
| Not relevant outcome (n=39) (34-72) |
| Both groups received low carbohydrate diet (n=14) (73-86) |
| Case-report/ review/ commentary (n=17) (87-103) |
| Conducted in children/ athlete/ pregnant (n=13) (104-116) |
| Deviation from prescribe diet (n=2) (117, 118) |
| Not relevant intervention (n=44) (19, 119-161) |
| Mixed population (normal weight and obese) (n=131) (162-291) |
| In patients without obesity (n=16) (292-307) |
| Observational study (n=9) (308-316) |
| Short duration (n=12) (317-328) |
| Not sufficient information (n=8) (329-336) |
| Under Bypass surgery (n=4) (337-340) |
| Without control group (n=29) (341-369) |
| Duplicates (n=51) (370-420) |

| Table S4- Study quality and risk of bias assessment of included studies according to the Cochrane tool. | | | | | | | |
| --- | --- | --- | --- | --- | --- | --- | --- |
| Author, Year | Random sequence generation | Allocation concealment | Blinding of participants | Blinding of outcome assessment | Incomplete outcome data | Selective reporting | Overall risk of bias |
| Abete, 2009 | U | U | U | U | L | L | High |
| Alessandro, 2015 | U | U | U | U | L | U | High |
| Aller, 2019 | L | U | U | U | L | L | High |
| Aronica, 2021 | L | L | U | U | L | L | Some concerns |
| Baba, 1999 | U | U | H | H | U | L | High |
| Ballesteros-Pomar, 2009 | L | L | L | L | L | L | Low |
| Barry, 2021 | H | U | U | U | U | U | High |
| Bazzano, 2014 | L | L | L | L | L | L | Low |
| Beavers, 2018 | L | U | U | U | L | L | High |
| Belobrajdic, 2010 | U | U | U | U | L | L | High |
| Bozzetto, 2012 | L | L | U | L | H | L | Some concerns |
| Bradley, 2009 | L | L | U | U | L | L | Some concerns |
| Brehm, 2003 | L | U | L | L | L | L | Some concerns |
| Brehm, 2005 | L | U | L | L | L | L | Some concerns |
| Brinkworth, 2009 | L | U | H | U | L | L | High |
| Brinkworth*, 2004 | L | U | H | L | H | L | High |
| Buga, 2021 | L | U | L | L | L | L | Some concerns |
| Clifton, 2004 | L | U | U | U | L | L | High |
| Cornier, 2005 | L | U | L | L | L | U | Some concerns |
| Cuevas‑Sierra, 2021 | L | U | U | U | L | L | High |
| Cunha, 2020 | L | U | U | L | L | U | Some concerns |
| Dale, 2009 | L | L | L | L | L | U | Some concerns |
| Daly, 2005 | L | L | H | L | U | H | High |
| Dansinger, 2005 | L | L | L | L | L | L | Low |
| Das*, 2007 | L | L | L | L | L | L | Low |
| Davis, 2009 | L | L | U | L | L | L | Some concerns |
| de Bont, 1981 | U | U | U | L | L | L | High |
| de Luis, 2015 | L | U | L | L | L | U | Some concerns |
| de Luis*, 2007 | L | U | U | U | U | U | High |
| de Luis*, 2012 | U | U | U | U | L | H | High |
| de Luis*, 2019 | U | U | U | U | U | U | High |
| Due, 2004 | L | L | L | L | L | L | Low |
| Ebbeling, 2007 | L | L | H | L | H | U | High |
| Elhayany, 2009 | U | U | L | L | L | L | High |
| Evangelista, 2021 | L | L | U | U | L | L | Some concerns |
| Evangelista, 2009 | L | U | U | U | L | L | High |
| Farnsworth*, 2003 | U | U | U | U | L | L | High |
| Flechtner-Mors*, 2010 | U | U | U | U | H | L | High |
| Foster, 2010 | L | L | L | L | U | L | Some concerns |
| Frisch, 2009 | L | L | L | L | L | L | Low |
| Gardner, 2007 | L | L | L | L | L | L | Low |
| Gardner, 2016 | L | U | H | H | L | H | High |
| Goday, 2016 | U | U | U | L | U | L | High |
| Goss, 2020 | L | U | H | H | L | L | High |
| Hernandez, 2010 | L | U | H | H | L | U | High |
| Hjorth, 2019 | U | U | U | U | L | L | High |
| Holmer, 2021 | L | U | H | H | L | L | High |
| Hsu, 2021 | L | L | U | L | L | L | Some concerns |
| Jabekk, 2010 | U | U | L | L | L | L | High |
| Jenkins*, 2013 | L | L | H | L | U | U | High |
| Jesudason, 2013 | L | L | H | U | L | L | High |
| Kakoschke, 2021 | L | L | H | L | L | L | Some concerns |
| Keogh, 2007 | U | U | U | U | H | L | High |
| Keogh, 2008 | U | U | U | U | L | L | High |
| Kirk, 2009 | U | U | U | U | L | L | High |
| Kleiner, 2006 | U | U | U | U | L | L | High |
| Klemsdal, 2010 | U | U | L | L | U | L | High |
| Krauss, 2005 | L | U | U | U | H | H | High |
| Lasker, 2008 | L | U | U | U | H | U | High |
| Layman, 2005 | L | U | U | U | L | L | High |
| Layman, 2009 | U | U | U | U | H | L | High |
| Layman, 2003 | U | U | U | U | U | U | High |
| Lean, 1996 | U | U | L | L | L | L | High |
| Lim, 2009 | U | U | L | U | U | U | High |
| Liu, 2013 | U | U | L | L | L | L | High |
| Luger, 2013 | U | U | H | L | L | L | High |
| Luscombe, 2002 | L | U | U | U | H | L | High |
| Marco-Benedí, 2019 | L | L | L | L | L | L | Low |
| McLaughlin, 2006 | L | U | U | U | U | L | High |
| McLaughlin, 2007 | U | U | U | U | L | U | High |
| Meckling, 2004 | U | U | L | L | L | L | High |
| Mellberg, 2014 | L | L | U | L | L | L | Some concerns |
| Mitra, 2019 | L | U | U | U | L | L | High |
| Miyashita, 2004 | U | U | U | L | L | L | High |
| Mohammadi Zadeh, 2018 | L | U | U | U | L | L | High |
| Moran, 2006 | L | L | U | U | L | L | Some concerns |
| Moran, 2003 | L | L | U | L | L | L | Some concerns |
| Moreno, 2014 | U | U | H | L | L | L | High |
| Nickols-Richardson, 2005 | L | U | U | U | L | L | High |
| Nielsen, 2004 | U | U | U | U | L | L | High |
| Perna, 2019 | U | U | U | U | U | U | High |
| Perticone, 2019 | H | H | U | U | L | U | High |
| Petersen, 2005 | L | L | U | U | L | L | Some concerns |
| Petrisko, 2019 | L | L | L | U | L | U | Some concerns |
| Sargrad, 2005 | U | U | H | U | L | L | High |
| Saslow, 2017 | L | H | H | H | U | U | High |
| Sato, 2016 | L | L | H | U | U | L | High |
| Shai, 2008 | L | U | L | L | L | L | Some concerns |
| Sharman, 2004 | L | L | L | U | L | L | Some concerns |
| Soenen, 2012 | L | U | L | L | L | L | Some concerns |
| Stamets, 2003 | L | L | U | L | L | L | Some concerns |
| Summer, 2011 | L | L | U | L | U | U | Some concerns |
| Sun, 2019 | U | U | L | L | L | L | High |
| Te Morenga, 2011 | L | L | L | H | L | L | Some concerns |
| Thomson, 2010 | L | L | L | L | L | L | Low |
| Torbay, 2002 | U | U | H | H | L | L | High |
| Tricò, 2021 | L | U | U | U | L | L | High |
| Valsdottir, 2020 | L | L | H | H | L | L | High |
| Varady, 2011 | L | L | L | L | L | L | Low |
| Veum, 2017 | L | L | H | H | L | L | High |
| Volek, 2004 | L | U | U | U | L | U | High |
| Volek, 2008 | U | U | L | L | L | L | High |
| Waliłko, 2021 | L | U | H | H | H | L | High |
| Watson, 2016 | U | U | H | L | L | L | High |
| Westman, 2008 | U | H | H | H | U | U | High |
| Wolever, 2008 | L | L | U | L | L | U | Some concerns |
| Wycherley, 2010 | L | L | H | L | U | L | Some concerns |
| Wycherley, 2014 | L | U | H | L | H | L | High |
| Wycherley, 2012 | U | U | L | L | L | L | High |
| Yancy, 2004 | L | U | U | U | L | L | High |
| Yancy, 2010 | L | L | L | L | L | L | Low |

| **Table S5-** Characteristics of trials that investigated the effect of low carbohydrate diet on body weight (kg) in overweight and obese adults. | | | | | | | |
| --- | --- | --- | --- | --- | --- | --- | --- |
| Author (Year)/ Country | Study design/ duration (week) | Sex/ (Mean age intervention/control) | Number of participants (intervention/control), Health status | Co-intervention | | Adherence report (yes/no) | Side effect |
|  |  |  |  | Calorie restriction (kcal/d)  (yes/no; if yes, degree of calorie restriction/which study arm) | Exercise (yes/no; if yes, which study arm) |  |  |
| Abete (2009)/ Spain | P / 8 | Male (36/36) | (9/9), Healthy | Yes (Deficit of 30% of TEE/ both) | No | No | No |
| Alessandro (2015)/ Italy | P / 8 | Female (51.4/ 44.7) | (16/16), Healthy | Yes (938-1400/ both) | Yes (both) | No | No |
| Aller (2019)/ Spain | P / 39 | Both (48.9/ 48.8) | (137/132), Healthy | Yes (1050 in the intervention and 1093 in the control arm) | Yes (both) | Yes | No |
| Aronica (2021)/ US | P / 52 | Both (40.2/39.3) | (220/216), Healthy | No | No | No | No |
| Baba (1999)/ Lebanon | P / 4 | Male (Nm) | (7/6), Hyper -insulinemia | Yes (Deficit of 80% of RER /both) | No | No | No |
| Ballesteros-Pomar (2009)/ Spain | P / 16 | Both (41.2/42.6) | (18/18), Mixed^#^ | Yes (Deficit of 1000 /both) | No | No | No |
| Barry (2021)/ US | P / 15 | Both (52.8/ 48.4) | (32/18), T2D | Yes (Deficit of 500/ both) | No | Yes | No |
| Bazzano (2014)/ US | P / 52 | Both (45.8/ 47.8) | (75/73), Healthy | No | No | Yes | Yes |
| Belobrajdic (2010)/ Australia | P / 12 | Male (51/51) | (34/42), Healthy | Yes (1672 / both) | No | No | No |
| Bozzetto (2012)/ Italy | P / 8 | Both (57/ 60.6) | (17/19), T2D | No | Yes (both) | Yes | No |
| Bradley (2009)/ UK | P / 8 | Both (37.1/ 40.5) | (12/12), Healthy | Yes (Deficit of 500 / both) | No | No | No |
| Brehm (2003)/ US | P/ 26 | Female (44.22/ 43.1) | (22/20), Healthy | Yes (1156 in the intervention and 1245 in the control arm) | No | No | No |
| Brehm (2005)/ US | P / 17 | Female (44.8/ 41.4) | (20/20), Healthy | Yes (1339/ both) | No | No | No |
| Brinkworth (2004)/ Australia | P / 64 | Both (60.9/62.7) | (19/19), T2D | No | No | No | No |
| Brinkworth (2009)/ Australia) | P / 52 | Both (51.5/ 51.4) | (33/36), Healthy | Yes (1600-1671/ both) | No | No | No |
| Buga (2021)/ US | P / 6 | Both (35/35) | (25/12), Healthy | Yes (Deficit of 75% EER/ both) | No | No | No |
| Clifton (2004/ Australia | P / 12 | Female (47.1/ 46.9) | (13/11), Healthy | No | No | No | No |
| Cornier (2005)/ US | P / 16 | Female (42.3/40.8) | (11/10), Mixed^#^ | Yes (Deficit of 400 / both) | No | No | No |
| Cuevas‑Sierra (2021)/ Spain | P / 17 | Both (Nm) | (82/97), Healthy | Yes (>1200 / both) | NR | No | No |
| Cunha (2020)/ US | P / 8 | Both (40.3/ 40.3) | (22/24), Healthy | Yes (600-800 in the intervention and 1400-1800 in the control arm) | Yes (both) | Yes | No |
| Dale (2009)/ New Zealand | P / 104 | Female (45/ 45) | (100/100), Healthy | No | Yes (both) | No | No |
| Daly (2005)/ UK | P / 13 | Both (58.2/ 59.1) | (51/51), T2D | No | Yes (both) | No | No |
| Dansinger (2005)/ US | P / 52 | Both (49/49) | (80/40), Healthy | No | Yes (both) | Yes | No |
| Das (2007)/ US | P / 52 | Both (35/34) | (17/17), Healthy | Yes (≥1900/ both) | No | Yes | No |
| Davis (2009)/ US | P / 52 | Both (54/53) | (55/50), T2D | No | Yes (both) | Yes | No |
| de Luis (2015)/ Spain | P / 39 | Both (50.5/49.9) | (168/163), Healthy | Yes (1101.8 in the intervention and 1193.8 in the control arm) | Yes (both) | Yes | No |
| de Luis (2007)/ Spain | P / 13 | Both (45.6/45.6) | (52/66), Healthy | No | Yes (both) | No | No |
| de Luis (2012)/ Spain | P / 13 | Both (43.2 /43.2) | (147/158), Healthy | No | Yes (both) | Yes | No |
| de Luis (2019)/ Spain | P / 39 | Both (46.7/46.7) | (137/133), Healthy | Yes (1050 in the intervention and 1093 in the control arm) | Yes (both) | No | No |
| de Bont (1981)/ UK | P / 26 | Female (54/ 56) | (35/34), T2D | Yes (1340 in the intervention and 1197 in the control arm) | No | No | No |
| Ebbeling (2007)/ US) | P / 78 | Both (28.2/26.9) | (36/37), Healthy | No | Yes (both) | Yes | No |
| Elhayany (2009)/ Israel | P / 52 | Both (55.5/ 56.7) | (63/116), T2D | No | Yes (both) | Yes | No |
| Evangelista (2021)/ US | P / 13 | Both (57.3/ 58) | (33/43), T2D | Yes (1200 or 1500/ both) | Yes (both) | Yes | No |
| Evangelista (2009)/ US | P / 12 | Both (56.4/ 60.4) | (5/9), T2D | Yes (1200 and 1500/ both) | No | Yes | No |
| Farnsworth (2003)/ Australia | P / 16 | Both (51.2/ 49.6) | (28/28), Hyper-insulinemia | No | No | No | No |
| Flechtner-Mors (2010)/ Germany | P / 52 | Both (49.3/ 50.2) | (31/49), MetS | Yes (Deficit of 500/ both) | No | No | No |
| Foster (2010)/ US | P / 104 | Both (46.2/ 44.9) | (89/105), Healthy | Yes (1200-1800/ both) | No | Yes | Yes |
| Frisch (2009)/ Germany | P / 52 | Both (47/ 47) | (100/100), Healthy | Yes (Deficit of 500/ both) | No | Yes | No |
| Gardner (2007)/ US | P / 52 | Female (41,40) | (156/79), Healthy | Yes (1478 -1658 both) | No | Yes | No |
| Gardner (2016)/ US | P / 26 | Both (42.5/42.5) | (30/31), Mixed^#^ | No | Yes (both) | Yes | No |
| Goday (2016)/ Spain | P / 17 | Both (54.89/ 54.17) | (45/40), T2D | Yes (Deficit 500-1000/ both) | No | Yes | Nm |
| Goss (2020)/ UK | P / 8 | Both (70.3/ 70.1) | (19/15), Healthy | No | No | Yes | No |
| Hernandez (2010)/ US | P / 6 | Both (42.8/ 43.4) | (16/16), Healthy | Yes (1200-1800/ both) | No | Yes | No |
| Hjorth (2019)/ Denmark | P / 104 | Both (45.5/ 45.5) | (86/105), Healthy | Yes (1200-1800/ both) | Yes (both) | No | No |
| Holmer (2021)/ Sweden | P / 12 | Both (56/ 56.5) | (25/49), NAFLD | Yes (1600-1900 / both) | No | Yes | Yes |
| Hsu (202`1)/Taiwan | P / 12 | Both (58.6/ 58) | (23/46), Healthy | No | Yes (both) | No | No |
| Jabekk (2010)/ Norway | P / 10 | Female (20-40) ^*^ | (8/8), Healthy | No | Yes (both) | No | Yes |
| Jenkins (2013)/ US | P / 26 | Both (57.6/ 55.3) | (20/19), Hyperlipidemia | Yes (1388 in the intervention and 1347 in the control arm) | No | Yes | No |
| Jesudason (2013)/ Australia | P / 52 | Both (59.4/ 62.4) | (21/24), T2D | No | No | No | No |
| Kakoschke (2021)/ Australia | P / 104 | Both (58.5/ 58.5) | (58/57), T2D | Yes (Deficit 500-1000/both) | Yes (both) | No | No |
| Keogh (2007)/ Australia | P / 52 | Both (50.1/ 46.9) | (7/6), Healthy | Yes (Deficit 30% of estimated usual intake/ both) | No | No | No |
| Keogh (2008)/ Australia | P / 8 | Both (50.5/ 49.4) | (52/47), Healthy | Yes (1433 – 1671/ both) | No | Yes | No |
| Kleiner (2006)/ US | P / 8 | Both (35.2/ 34.6) | (7/9), Hyper-insulinemia | Yes (Deficit 70-75% TEE/ both) | No | No | No |
| Klemsdal (2010)/ Norway | P / 52 | Both (50.1/ 49.9) | (100/102), Healthy | Yes (Deficit of 500/ both) | No | No | No |
| Krauss (2005)/ US | P / 9 | Male (49.6/ 50.3) | (129/49), Healthy | Yes (1000 only for 5 week / both) | No | Yes | No |
| Kirk (2009)/ US | P / 11 | Both (41.8/ 45.4 | (11/11), Insulin resistance | Yes (Deficit of 1000/ both) | No | No | No |
| Lasker (2008)/ US | P / 17 | Both (47/ 47) | (25/25), Healthy | Yes (1700 formulated/ both) | Yes | No | No |
| Layman (2005)/ US | P / 17 | Female (46.7/ 46.5) | (24/24), Healthy | Yes (1700/ both) | Yes (both) | No | No |
| Layman (2009)/ US | P / 52 | Both (45.2/ 46) | (64/66), Healthy | Yes (1700 – 1900/ both) | Yes (both) | No | No |
| Layman (2003)/ US | P / 10 | Female (50.1/ 50.1) | (12/12), Healthy | Yes (1700/ both) | No | No | No |
| Lean (1996)/ UK | P / 26 | Female (50.1/ 51.1) | (53/57), Healthy | Yes (1200/ both) | No | No | No |
| Lim (2009)/ Australia | P / 65 | Both (48.3/ 47.9) | (17/33), Healthy | No | No | Yes | No |
| Liu (2013)/ Chinese | P / 12 | Female (47.9/ 47.9) | (25/24), Healthy | Yes (1300/ both) | No | Yes | Yes |
| Luger (2013)/ Europe | P / 12 | Both (61/ 63.7) | (21/22), T2D | No | No | Yes | No |
| Luscombe (2002)/ Australia | P / 8 | Both (62.1/ 64.2) | (15/11), T2D | Yes (1600/ both) | No | No | No |
| Luscombe (2003)/ Australia | P / 12 | Both (55/ 53) | (17/19), Hyper-insulinemia | Yes (Deficit 30% total energy/ both) | No | No | No |
| Marco-Benedí (2019)/ Spain | P / 26 | Both (56.5/ 54.6) | (35/32), T2D | Yes (1200-2000/ both) | Yes (both) | Yes | No |
| McLaughlin (2006)/ US | P / 16 | Both (48/ 53) | (27/30), Insulin resistance | Yes (Deficit of 750/ both) | No | Yes | No |
| McLaughlin (2007)/ US | P / 16 | Both (57/ 56) | (14/15), T2D | Yes (Deficit of 750/ both) | No | No | No |
| Meckling (2004)/ US | P / 10 | Both (41.2/ 43.2) | (15/16), Healthy | Yes (1200–2200/ both) | No | No | No |
| Mellberg (2014)/ Sweden | P / 104 | Female (59.5/ 60.3) | (27/22), Healthy | No | No | Yes | No |
| Mitra (2019)/ Malaysia | P / 26 | Both (44.2/ 45.4) | (65/63), Healthy | Yes (Deficit of 300-500/ both) | No | Yes | No |
| Miyashita (2004)/ Japan | P / 4 | Both (52.4/ 52.4) | (11/11), T2D | Yes (Deficit of 1000/ both) | Yes (both) | No | No |
| Mohammadi Zadeh (2018)/ Iran | P / 24 | Male (45.8/ 47.2) | (22/20), T2D | No | Yes (both only for 12 weeks) | No | No |
| Moran (2006)/ Australia | P / 9-32 | Female (32.1/ 33.2) | (14/14), PCOS | No | No | Yes | No |
| Moran (2003)/ Australia | P / 12 | Female (32/ 33) | (14/14), PCOS | Yes (1433/ both) | Yes (both) | Yes | No |
| Moreno (2014)/ Spain | P / 52 | Both (46.3/ 44.4) | (27/26), Prediabetes | Yes (1500 – 2000/ both) | Yes (both) | Yes | No |
| Nicklos-richardson (2005)/ US | P / 6 | Female (38.8/ 40.1) | (13/15), Healthy | No | No | No | No |
| Nielsen (2004)/ Sweden | P / 26 | Both (57.1/ 58.6) | (16/15), T2D | Yes (1600-1800 in the intervention and 1400-1800 in the control arm) | Yes (both) | No | No |
| Perna (2019)/ Italy | P / 13 | Both (59.5/ 67.8) | (8/9), T2D | Yes (1600 / both) | No | No | No |
| Perticone (2019)/ Italy | P / 52 | Both (42.6/ 50.9) | (28/28), Healthy | Yes (Deficit of 500 for control and deficit of 600 for intervention arm) | No | Yes | No |
| Petersen (2005)/ Denmark | P / 10 | Both (20-50) ^*^ | (312/336), Healthy | Yes (Deficit of 600/ both) | No | No | No |
| Petrisko (2019)/ US) | C / 4 (4 weeks wash out) | Both (43.2/ 43.2) | (34/17), Healthy | No | No | Yes | No |
| Sargrad (2005)/ US) | P / 8 | Both (48/ 47) | (6/6), T2D | No | No | No | No |
| Saslow (2017/ US) | P / 52 | Both (64.8/ 55.1) | (16/18), Prediabetes | Yes (1563/ both) | Yes (both) | Yes | No |
| Sato (2016)/ Japan | P / 26 | Both (60.5/ 58.4) | (30/32), T2D | Yes (2000/ both) | No | Yes | No |
| Shai (2008)/ Israel | P / 104 | Both (52/ 52) | (109/213), T2D | Yes (1500 – 1800/ both) | No | Yes | No |
| Sharman (2004)/ US | C / 6 (without wash out) | Male (33.2/ 33.2) | (15/15), Healthy | Yes (Deficit of 500/ both) | No | No | No |
| Soenen (2012)/ Netherlands | P / 52 | Both (50/ 50) | (33/33), Healthy | Yes (Deficit of 33% -67% of energy requirements / both) | No | No | No |
| Stamets (2003)/ US | P / 4 | Female (29/,26) | (13/13), PCOS | Yes (Deficit of 1000 / both) | No | No | No |
| Summer (2011)/ US | P / 17-26 | Female (44.5/ 41.9) | (42/39), Healthy | Yes (Nm/ both) | No | No | No |
| Sun (2019)/ China | P / 4 | Female (21.1 /21.6) | (28/15), Healthy | No | Yes (Intervention) | No | Yes |
| Te Morenga (2011)/ New Zealand | P / 8 | Female (40.5/ 43.4) | (42/41), MetS | Yes (Deficit of 478- 955 TEE / both) | No | Yes | No |
| Thomson (2010)/ US | P / 24 | Female (56.2/ 56.2) | (21/19), Healthy | Yes (Deficit of 500/ both) | No | No | No |
| Torbay (2002)/ Lebanon | P / 4 | Male (20-40) ^*^ | (14/13), Hyper-insulinemia | Yes (Deficit of 80% of REE/ both) | No | No | No |
| Tricò (2021)/ Italy | P / 4 | Both (41.4/ 46.9) | (17/15), Healthy | Yes (Deficit of 50% of REE/ both) | No | Yes | No |
| Valsdottir (2020)/ Norway | P / 10 | Female (40.4/ 39.5) | (28/29), Healthy | Yes (Deficit of 700/ both) | Yes (both) | No | Yes |
| Varady (2011)/ US | P / 6 | Both (35/ 36) | (9/8), Healthy | Yes (Deficit of 25% of energy needs/ both) | No | Yes | No |
| Veum (2017)/ Norway | P / 12 | Male (40.3/ 40.2) | (20/18), Healthy | No | No | No | No |
| Volek (2004)/ US | P / 4 | Female (34/ 34) | (13/13), Healthy | Yes (Deficit of 500/ both) | No | No | No |
| Volek (2008)/ US | P / 12 | Both (32.6/ 36.9) | (20/20), MetS | Yes (Deficit of 500/ both) | No | No | No |
| Waliłko (2021)/ Europe | C / 4 (without wash out) | Both (40.7/ 40.7) | (17/18), Healthy | Yes (Deficit of 600/ both) | No | No | No |
| Watson (2016)/ Australia | P / 24 | Both (54/ 55) | (32/29), T2D | No | Yes (both) | No | No |
| Westman (2008)/ US | P / 24 | Both (51.8/ 51.8) | (21/29), T2D | Yes (Deficit of 500/ both) | Yes (both) | No | No |
| Wolever (2008)/ US | P / 52 | Both (58.6/ 60.4) | (54/110), T2D | No | No | No | No |
| Wycherley (2010)/ Australia | P / 16 | Both (55/ 55) | (26/33), T2D | Yes (1433- 1671/ both) | Yes (both) | No | No |
| Wycherley (2014)/ Australia | P / 52 | Both (49.2/ 50.8) | (23/20), Healthy | Yes (1433- 1671/ both) | No | No | No |
| Wycherley (2012)/ Australia | P / 52 | Male (51.3/ 50.2) | (33/35), Healthy | Yes (Deficit of 500-1000/ both) | No | No | No |
| Yancy (2004)/ US | P / 24 | Both (44.2/ 45.6) | (59/60), Hyperlipidemia | Yes (Deficit of 500-1000/ both) | Yes (both) | Yes | Yes |
| Yancy (2010)/ US | P / 48 | Both (52.9/ 52) | (59/60), MetS | Yes (Deficit of 500-1000/ both) | Yes (both) | Yes | Yes |
| C: Cross-over; EER: estimated energy requirement; MetS: Metabolic syndrome; NAFLD: Non-alcoholic fatty liver disease; P: parallel, REE: Resting energy expenditure; TEE: Total Energy Expenditure; T2D: Type 2 diabetes. | | | | | | | |

| **Table S6**- The amount of dietary macronutrients (% per calorie) in low- and high-carbohydrate diets. | | | | | | | | | | | | |
| --- | --- | --- | --- | --- | --- | --- | --- | --- | --- | --- | --- | --- |
| Author (Year)/ Country | Prescribe diet (% per calorie) | | | | | | Self-reported diet (% per calorie) | | | | | |
|  | Low carbohydrate diet | | | High carbohydrate diet | | | Low carbohydrate diet | | | High carbohydrate diet | | |
|  | CHO | Protein | Fat | CHO | Protein | Fat | CHO | Protein | Fat | CHO | Proteins | Fat |
| Abete (2009)/ Spain | 40 | 30 | 30 | 55 | 15 | 30 | 43.2 | 30.7 | 28.5 | 55.9 | 17.5 | 30.1 |
| Alessandro (2015)/ Italy | 34 | 27 | 39 | 60 | 15 | 25 | NR | NR | NR | NR | NR | NR |
| Aller (2019)/ Spain | 33 | 34 | 33 | 53 | 20 | 27 | 41 | 27.2 | 39.8 | 43 | 20 | 39 |
| Aronica (2021)/ US | 10-26 | NR | NR | NR | NR | NR | 29.5 | 23 | 44.5 | 48.5 | 21 | 29 |
| Baba (1999)/ Lebanon | 25 | 45 | 30 | 58 | 12 | 30 | NR | NR | NR | NR | NR | NR |
| Ballesteros-Pomar (2009)/ Spain | 40 | 30 | 30 | 55 | 15 | 30 | 41 | 24 | 35 | 57 | 16 | 25 |
| Barry (2021)/ US | 5 | 30 | 65 | 63 | 13-23 | 10–25 | NR | NR | NR | NR | NR | NR |
| Bazzano (2014)/ US | <10 | NR | NR | 55 | NR | <30 | 28.9 | 25.6 | 42.7 | 52.9 | 19 | 27.5 |
| Belobrajdic (2010)/ Australia | NR | 34 | NR | NR | NR | NR | 36.9 | 32.6 | 27.5 | 50.7 | 20.5 | 25.7 |
| Bozzetto (2012)/ Italy | 40 | 18 | 42 | 52 | 18 | 30 | 40 | 18 | 42 | 53 | 18.5 | 28.5 |
| Bradley (2009)/ UK | 20 | 20 | 60 | 60 | 20 | 20 | 40 | 15 | 40 | 41 | 16 | 39 |
| Brehm (2003)/ US | <10 | NR | NR | 55 | 15 | 30 | 30 | 23 | 46 | 53 | 18 | 29 |
| Brehm (2005)/ US | <10 | NR | NR | 55 | 15 | 30 | 24 | 24 | 52 | 48 | 20 | 32 |
| Brinkworth (2004)/ Australia | 40 | 30 | 30 | 55 | 15 | 30 | NR | NR | NR | NR | NR | NR |
| Brinkworth (2009)/ Australia) | <10 | 35 | 61 | 35 | 24 | 30 | 9 | 32 | 55 | 46 | 22 | 26 |
| Buga (2021)/ US | 8.7 | 22.1 | 68.5 | 54.2 | 21 | 24.2 | NR | NR | NR | NR | NR | NR |
| Clifton (2004/ Australia | 44 | 21 | 35 | 67 | 21 | 12 | 43.7 | 21.3 | 35.3 | 65.4 | 21.7 | 11.6 |
| Cornier (2005)/ US | 40 | 20 | 40 | 60 | 20 | 20 | NR | NR | NR | NR | NR | NR |
| Cuevas‑Sierra (2021)/ Spain | 40 | 30 | 30 | 60 | 18 | 22 | NR | NR | NR | NR | NR | NR |
| Cunha (2020)/ US | <10 | NR | NR | 45-55 | 15-25 | 25-35 | NR | NR | NR | NR | NR | NR |
| Dale (2009)/ New Zealand | NR | NR | NR | NR | NR | NR | 43 | 22 | 31 | 47 | 22 | 27 |
| Daly (2005)/ UK | <26 | NR | NR | NR | NR | NR | 34 | 26 | 40 | 45 | 21 | 33 |
| Dansinger (2005)/ US | <25 | 30 | 30 | NR | NR | NR | 39.8 | 19.4 | 37.5 | 45.4 | 18 | 31.4 |
| Das (2007)/ US | 40 | 30 | 30 | 60 | 20 | 20 | NR | NR | NR | NR | NR | NR |
| Davis (2009)/ US | <26 | NR | NR | NR | NR | 25 | 33 | 23 | 44 | 50 | 19 | 31 |
| de Luis (2015)/ Spain | 33 | 34 | 33 | 53 | 20 | 27 | 34.1 | 35.9 | 33 | 52.3 | 18.8 | 28.9 |
| de Luis (2007)/ Spain | 38 | 26 | 36 | 52 | 20 | 27 | 31 | NR | NR | NR | NR | 25 |
| de Luis (2012)/ Spain | 38 | 26 | 36 | 53 | 20 | 27 | 31 | NR | NR | NR | NR | 25 |
| de Luis (2019)/ Spain | 33 | 34 | 33 | 53 | 20 | 27 | NR | NR | NR | NR | NR | NR |
| de Bont (1981)/ UK | 40 | NR | NR | 46 | NR | 30 | 38 | 19.9 | 41 | 45.7 | 22.7 | 31 |
| Ebbeling (2007)/ US) | 40 | 25 | 35 | 55 | 25 | 20 | 41.3 | 21.4 | 36.09 | 53.7 | 21.6 | 25.5 |
| Elhayany (2009)/ Israel | 35 | 15-20 | 45 | 50-55 | 18.7 | 30 | NR | NR | NR | NR | NR | NR |
| Evangelista (2021)/ US | 40 | 30 | 30 | 55 | 15 | 30 | NR | NR | NR | NR | NR | NR |
| Evangelista (2009)/ US | 40 | 30 | 30 | 55 | 15 | 30 | NR | NR | NR | NR | NR | NR |
| Farnsworth (2003)/ Australia | 40 | 30 | 30 | 55 | 15 | 30 | 44.6 | 27.3 | 26.9 | 56.9 | 15.4 | 27.5 |
| Flechtner-Mors (2010)/ Germany | 40 | 30 | 30 | 55 | 15 | 30 | 36.9 | 30 | 29.9 | 47.6 | 21.4 | 29.6 |
| Foster (2010)/ US | 10-26 | NR | NR | 55 | 15 | 30 | NR | NR | NR | NR | NR | NR |
| Frisch (2009)/ Germany | 40 | 25 | 35 | 55 | 15 | 30 | 43.5 | 18.9 | 34.2 | 50.1 | 16.7 | 30.2 |
| Gardner (2007)/ US | <25 | 30 | 30 | 55-60 | NR | NR | 39.9 | 20.3 | 39.4 | 49.8 | 18.6 | 31.3 |
| Gardner (2016)/ US | <10 | NR | NR | NR | NR | NR | 21 | 26 | 53 | 58 | 21 | 22 |
| Goday (2016)/ Spain | <10 | NR | NR | 45-60 | 10-20 | <30 | NR | NR | NR | NR | NR | NR |
| Goss (2020)/ UK | ≤10 | 25 | ≥65 | 55 | 25 | 20 | NR | NR | NR | NR | NR | NR |
| Hernandez (2010)/ US | <10 | NR | NR | 55 | 15 | 30 | NR | NR | NR | NR | NR | NR |
| Hjorth (2019)/ Denmark | 10-26 | NR | NR | 55 | 15 | 30 | NR | NR | NR | NR | NR | NR |
| Holmer (2021)/ Sweden | 5-10 | 15-40 | 50-80 | 45-60 | 10-20 | 25 | 7.5 | 19.3 | 65.6 | 40.8 | 10.4 | 11.5 |
| Hsu (202`1)/Taiwan | NR | 25.6 | NR | NR | NR | NR | 41.7 | 26 | 31.9 | 50.8 | 16.9 | 31.7 |
| Jabekk (2010)/ Norway | <10 | NR | NR | NR | NR | NR | 6 | 22 | 66 | 41 | 17 | 34 |
| Jenkins (2013)/ US | 26 | 31 | 43 | 58 | 16 | 25 | 40 | 23 | 36 | 54 | 19 | 28 |
| Jesudason (2013)/ Australia | 40 | 30 | 30 | 50 | 20 | 30 | NR | NR | NR | NR | NR | NR |
| Kakoschke (2021)/ Australia | 14 | 28 | 58 | 53 | 17 | <30 | NR | NR | NR | NR | NR | NR |
| Keogh (2007)/ Australia | 33 | 40 | 27 | 60 | 20 | 20 | NR | NR | NR | NR | NR | NR |
| Keogh (2008)/ Australia | 4 | 35 | 61 | 46 | 24 | 30 | 5 | 35 | 59 | 47 | 24 | 28 |
| Kleiner (2006)/ US | 41 | 32 | 27 | 59 | 14 | 27 | NR | NR | NR | NR | NR | NR |
| Klemsdal (2010)/ Norway | 30-35 | 25-30 | 35-40 | 55-60 | 15 | <30 | NR | NR | NR | NR | NR | NR |
| Krauss (2005)/ US | 26 | 29 | 45.5 | 54 | 16 | 30 | NR | NR | NR | NR | NR | NR |
| Kirk (2009)/ US | 10 | 15 | 75 | 65 | 15 | 20 | NR | NR | NR | NR | NR | NR |
| Lasker (2008)/ US | 40 | 30 | 30 | 55 | 15 | 30 | 39 | 31 | 33 | 61 | 19 | 25 |
| Layman (2005)/ US | 40 | 30 | 30 | 55 | 15 | 30 | 38.6 | 30.6 | 31.7 | 60.6 | 17.3 | 25.4 |
| Layman (2009)/ US | 40 | 30 | 30 | 55 | 15 | 30 | 39.27 | 27.11 | 35.07 | 57.06 | 17.29 | 28.15 |
| Layman (2003)/ US | 40 | 30 | <30 | 55 | 15 | <30 | 41 | 30 | 29 | 58 | 16 | 26 |
| Lean (1996)/ UK | 35 | NR | NR | 55 | NR | NR | 34.5 | 29.9 | 35 | 58 | 20.7 | 20.5 |
| Lim (2009)/ Australia | 4 | 35 | 60 | 60 | 20 | 20 | 36.5 | 23.9 | 37.1 | 46.8 | 22 | 29.3 |
| Liu (2013)/ Chinese | <10 | NR | NR | 50-55 | 17-19 | 26-33 | 36.1 | 26 | 37.9 | 51.1 | 17.7 | 31.2 |
| Luger (2013)/ Europe | 40 | 30 | 30 | 55 | 15 | 30 | 37.5 | 27 | 35 | 50 | 17 | 29 |
| Luscombe (2002)/ Australia | 40 | 30 | 30 | 55 | 15 | 30 | 42 | 28 | 28 | 55 | 16 | 26 |
| Luscombe (2003)/ Australia | 40 | 30 | 30 | 55 | 15 | 30 | 45 | 27 | 27 | 57 | 16 | 27 |
| Marco-Benedí (2019)/ Spain | 35 | 35 | 30 | 52 | 18 | 30 | NR | NR | NR | NR | NR | NR |
| McLaughlin (2006)/ US | 40 | 15 | 45 | 60 | 15 | 25 | 41 | 18 | 41 | 57 | 18 | 25 |
| McLaughlin (2007)/ US | 40 | 15 | 45 | 60 | 15 | 25 | NR | NR | NR | NR | NR | NR |
| Meckling (2004)/ US | <26 | NR | NR | NR | NR | NR | 15.4 | 26.2 | 55.5 | 61.9 | 19.5 | 17.8 |
| Mellberg (2014)/ Sweden | 30 | 30 | 40 | 55-60 | 15 | 25-30 | NR | NR | NR | NR | NR | NR |
| Mitra (2019)/ Malaysia | 40 | 30 | 30 | 55-70 | 10-15 | 20-30 | 43 | 27 | 32 | 50 | 15.3 | 37.5 |
| Miyashita (2004)/ Japan | 39 | 25 | 35 | 62 | 26 | 10 | NR | NR | NR | NR | NR | NR |
| Mohammadi Zadeh (2018)/ Iran | 25 | 22.5 | 52.5 | 50 | 17.5 | 32.5 | NR | NR | NR | NR | NR | NR |
| Moran (2003)/ Australia | 40 | 30 | 30 | 55 | 15 | 30 | NR | NR | NR | NR | NR | NR |
| Moran (2006)/ Australia | <26 | NR | NR | NR | NR | NR | 40 | 21 | 35 | 43 | 21 | 31 |
| Moreno (2014)/ Spain | <26 | Nm | Nm | 45–55 | 15–25 | 25–35 | NR | NR | NR | NR | NR | NR |
| Nicklos-richardson (2005)/ US | <10 | Nm | Nm | 60 | 15 | 25 | 12 | 26 | 61 | 60 | 18 | 22 |
| Nielsen (2004)/ Sweden | 20 | 30 | 50 | 60 | 15 | 25 | NR | NR | NR | NR | NR | NR |
| Perna (2019)/ Italy | 30.2 | 22.5 | 47.2 | 57.7 | 18.8 | 24.3 | NR | NR | NR | NR | NR | NR |
| Perticone (2019)/ Italy | 20 | 50-60 | 20-30 | 55-60 | 10-15 | 25-30 | NR | NR | NR | NR | NR | NR |
| Petersen (2005)/ Denmark | 40-45 | 15 | 40-45 | 60-65 | 15 | 20-25 | 43 | 17 | 40 | 57 | 18 | 25 |
| Petrisko (2019)/ US) | 10 | 35 | 55 | 61 | 18 | 21 | NR | NR | NR | NR | NR | NR |
| Sargrad (2005)/ US) | 40 | 30 | 30 | 55 | 15 | 30 | 43 | 27 | 30 | 51 | 19 | 30 |
| Saslow (2017/ US) | <10 | Nm | Nm | 45-50 | NR | NR | 19.2 | 25.43 | 61.8 | 35.64 | 16.37 | 40.3 |
| Sato (2016)/ Japan | 26-44 | NR | NR | 50-60 | NR | NR | 42.98 | 18.38 | 34.35 | 49.26 | 16.03 | 12.87 |
| Shai (2008)/ Israel | <26 | NR | NR | NR | NR | 32.5 | 40.4 | 21.8 | 39.1 | 50.4 | 18.9 | 31.5 |
| Sharman (2004)/ US | 10 | 30 | 60 | 55 | 20 | 25 | 8 | 28 | 63 | 56 | 20 | 23 |
| Soenen (2012)/ Netherlands | 5 | 45 | 50 | 35 | 45 | 20 | NR | NR | NR | NR | NR | NR |
| Stamets (2003)/ US | 40 | 30 | 30 | 55 | 15 | 30 | NR | NR | NR | NR | NR | NR |
| Summer (2011)/ US | NR | NR | NR | NR | NR | NR | 27 | 24 | 49 | 50 | 19 | 31 |
| Sun (2019)/ China | 10 | 25 | 65 | NR | NR | NR | 10 | 23.3 | 66.6 | 46 | 15 | 37 |
| Te Morenga (2011)/ New Zealand | 40 | 30 | <30 | 50 | 20 | <30 | 40 | 28 | 29 | 51 | 22 | 23 |
| Thomson (2010)/ US | 35 | 25–30 | 35–40 | 55–60 | 15–20 | 25 | NR | NR | NR | NR | NR | NR |
| Torbay (2002)/ Lebanon | 25 | 45 | 30 | 58 | 12 | 30 | NR | NR | NR | NR | NR | NR |
| Tricò (2021)/ Italy | 30 | 30 | 40 | 55 | 15 | 30 | NR | NR | NR | NR | NR | NR |
| Valsdottir (2020)/ Norway | 14 | 25 | 61 | 52 | 15 | 33 | 31 | 24 | 61.5 | 49 | 19.5 | 31 |
| Varady (2011)/ US | 5 | 35 | 60 | 55 | 20 | 35 | NR | NR | NR | NR | NR | NR |
| Veum (2017)/ Norway | 10 | 17 | 73 | 53 | 17 | 30 | 11 | 16.8 | 71 | 51 | 17 | 29 |
| Volek (2004)/ US | <10 | <30 | 60 | 55 | 20 | 25 | 9 | 28 | 63 | 59 | 19 | 21 |
| Volek (2008)/ US | 12 | 28 | 59 | 56 | 20 | 24 | 12 | 28 | 59 | 56 | 20 | 24 |
| Waliłko (2021)/ Europe | 40 | 30 | 30 | 50 | 20 | 30 | 39 | 30 | 31 | 45 | 24 | 31 |
| Watson (2016)/ Australia | 33 | 32 | 30 | 51 | 22 | 22 | 34 | 29 | 32 | 47 | 20 | 25.1 |
| Westman (2008)/ US | <10 | Nm | Nm | 55 | Nm | Nm | Nm | Nm | Nm | Nm | Nm | Nm |
| Wolever (2008)/ US | Nm | Nm | Nm | Nm | Nm | Nm | 39.3 | 19.1 | 40.1 | 49.2 | 20.5 | 28.6 |
| Wycherley (2010)/ Australia | 43 | 33 | 22 | 53 | 19 | 26 | 46.4 | 31.9 | 18.6 | 53.6 | 18.6 | 22.4 |
| Wycherley (2014)/ Australia | 4 | 35 | 61 | 46 | 24 | 30 | 8 | 34 | 57 | 48 | 24 | 26 |
| Wycherley (2012)/ Australia | 40 | 35 | 25 | 58 | 17 | 25 | 35.9 | 30.7 | 29.8 | 47.3 | 20.4 | 52.2 |
| Yancy (2004)/ US | <10 | Nm | Nm | Nm | Nm | <30 | 8 | 26 | 68 | 52 | 19 | 29 |
| Yancy (2010)/ US | <10 | Nm | Nm | Nm | Nm | <30 | 14.6 | 26.43 | 56.55 | 47.61 | 19.92 | 32.7 |
| CHO, carbohydrate; NR: not reported. | | | | | | | | | | | | |

| Table S7- Subgroup analysis of the effect of carbohydrate restriction (10% decrease) on body weight (kg) at 6-month follow-up. | | | | | | | |
| --- | --- | --- | --- | --- | --- | --- | --- |
| Study group | No. of studies | Meta-analysis | | Heterogeneity | | | |
|  |  | Mean difference  (95% CI) | P value | Q statistic | P within group | I^2^ (%) | P between group |
| **Overall** | 101 | -0.64 (-0.79, -0.49) | <0.001 | 573.81 | <0.001 | 80.5 |  |
| **Study design** |  |  |  |  |  |  | <0.001 |
| Parallel | 98 | -0.65 (-0.81, -0.50) | <0.001 | 503.61 | <0.001 | 78.4 |  |
| Cross-over | 3 | -0.15 (-0.26, -0.05) | 0.005 | 1.69 | 0.43 | 0 |  |
| **Sex** |  |  |  |  |  |  | 0.03 |
| Male | 12 | -0.34 (-0.67, -0.02) | 0.04 | 29.96 | 0.003 | 59.9 |  |
| Female | 27 | -0.45 (-0.72, -0.18) | 0.001 | 165.03 | <0.001 | 82.4 |  |
| **Health status** |  |  |  |  |  |  | 0.003 |
| Healthy | 61 | -0.46 (-0.62, -0.29) | <0.001 | 348.66 | <0.001 | 81.1 |  |
| Unhealthy | 40 | -1.00 (-1.31, -0.68) | <0.001 | 183.10 | <0.001 | 75.4 |  |
| **Exercise** |  |  |  |  |  |  | 0.20 |
| Yes | 30 | -0.84 (-1.12, -0.56) | <0.001 | 152.30 | <0.001 | 78.3 |  |
| No | 71 | -0.54 (-0.72, -0.37) | <0.001 | 403.47 | <0.001 | 80.9 |  |
| **Calorie restriction** |  |  |  |  |  |  | 0.56 |
| Yes | 35 | -0.80 (-1.08, -0.51) | <0.001 | 241.12 | <0.001 | 85.5 |  |
| No | 66 | -0.57 (-0.74, -0.39) | <0.001 | 312.45 | <0.001 | 75.5 |  |
| **Dietary report** |  |  |  |  |  |  | 0.95 |
| Self-reported | 59 | -0.65 (-0.82, -0.47) | <0.001 | 205.69 | <0.001 | 68.4 |  |
| Prescribed | 42 | -0.63 (-0.88, -0.40) | <0.001 | 311.29 | <0.001 | 85.2 |  |
| **Control diet** |  |  |  |  |  |  | 0.32 |
| Low fat | 40 | -0.75 (-1.02, -0.47) | <0.001 | 303.39 | <0.001 | 85.8 |  |
| Usual diet | 61 | -0.58 (-0.75, -0.41) | <0.001 | 260.29 | <0.001 | 73.9 |  |
| **Protein (%)** |  |  |  |  |  |  | 0.17 |
| <20 | 16 | -0.23 (-0.74, 0.29) | 0.38 | 143.51 | <0.001 | 86.8 |  |
| 20-24.9 | 22 | -0.57 (-0.79, -0.35) | <0.001 | 31.38 | 0.09 | 29.9 |  |
| ≥ 25 | 64 | -0.72 (-0.90, -0.53) | <0.001 | 391.52 | <0.001 | 82.4 |  |
| **Risk of bias** |  |  |  |  |  |  | 0.54 |
| High | 67 | -0.62 (-0.79, -0.44) | <0.001 | 268.28 | <0.001 | 72.8 |  |
| Some concerns | 25 | -0.71 (-1.03, -0.38) | <0.001 | 286.63 | <0.001 | 90.2 |  |
| Low | 9 | -0.83 (-1.18, -0.48) | <0.001 | 9.05 | 0.43 | 0.60 |  |

| **Table S8**. Assessment of credibility of subgroup difference for the effect of carbohydrate restriction (10% decrease) on body weight (kg) at 6-month follow-up based on ICEMAN. | | | | | | | | | |
| --- | --- | --- | --- | --- | --- | --- | --- | --- | --- |
| Variable | Q1 | Q2 | Q3 | Q4 | Q5 | Q6 | Q7 | Q8 | Overall credibility |
| Study design | Completely between | Definitely not similar | Rather small | Probably no | Chance an unlikely explanation | Definitely yes | Definitely yes | Probably yes | Low |
| Sex | Completely between | Definitely not similar | Definitely yes (very large) | Probably no | Chance a likely explanation or unclear | Definitely yes | Definitely yes | Probably yes | Low |
| Health status | Completely between | Definitely not similar | Definitely yes (very large) | Probably no | Chance an unlikely explanation | Definitely yes | Definitely yes | Probably yes | Low |
| Exercise | Completely between | Definitely not similar | Definitely yes (very large) | Definitely yes | Chance a very likely explanation | Definitely yes | Definitely yes | Probably yes | Low |
| Calorie restriction | Completely between | Definitely not similar | Definitely yes (very large) | Definitely yes | Chance a very likely explanation | Definitely yes | Definitely yes | Probably yes | Low |
| Dietary report | Completely between | Definitely not similar | Definitely yes (very large) | Probably no | Chance a very likely explanation | Definitely yes | Definitely yes | Probably yes | Low |
| Control diet | Completely between | Definitely not similar | Definitely yes (very large) | Probably no | Chance a very likely explanation | Definitely yes | Definitely yes | Probably yes | Low |
| Protein intake | Completely between | Definitely not similar | Definitely yes (very large) | Definitely yes | Chance a very likely explanation | Definitely yes | Definitely yes | Probably yes | Low |
| Risk of bias | Completely between | Definitely not similar | Rather large | Probably no | Chance a very likely explanation | Definitely yes | Definitely yes | Probably yes | Low |
| **Abbreviations**: Q, question.  Q1, Is the analysis of effect modification based on comparison within rather than between trials? Q2, For within-trial comparisons, is the effect modification similar from trial to trial? Q3, For between-trial comparisons, is the number of trials large? Q4, Was the direction of the effect modification correctly hypothesized priori? Q5, Does a test for interaction suggest that chance is an unlikely explanation of the apparent effect modification? Q6, Did the authors test only a small number of effect modifiers? Q7, Did the authors use a random effects model? Q8, If the effect modifier is a continuous variable, were arbitrary cut points avoided? | | | | | | | | | |

| Table S9- Subgroup analysis of the effect of carbohydrate restriction (10% decrease) on body weight (kg) at 12-month follow-up. | | | | | | | |
| --- | --- | --- | --- | --- | --- | --- | --- |
| Study group | No. of studies | Meta-analysis | | Heterogeneity | | | |
|  |  | Mean difference  (95% CI) | P value | Q statistic | P within group | I^2^ (%) | P between group |
| **Overall** | 42 | -1.15 (-1.61, -0.69) | <0.001 | 644.62 | <0.001 | 93.2 |  |
| **Study design** |  |  |  |  |  |  | - |
| Parallel | 42 | -1.15 (-1.61, -0.69) | <0.001 | 644.62 | <0.001 | 93.2 |  |
| Cross-over | - | - | - | - | - | - |  |
| **Sex** |  |  |  |  |  |  | 0.24 |
| Male | 2 | -3.68 (-7.74, 0.38) | 0.07 | 5.34 | 0.02 | 81.3 |  |
| Female | 7 | -0.51 (-1.51, 0.49) | 0.32 | 49.37 | <0.001 | 87.8 |  |
| **Health status** |  |  |  |  |  |  | 0.99 |
| Healthy | 26 | -1.15 (-1.71, -0.58) | <0.001 | 579.20 | <0.001 | 95.2 |  |
| Unhealthy | 16 | -1.14 (-1.93, -0.36) | 0.004 | 63.50 | <0.001 | 76.4 |  |
| **Exercise** |  |  |  |  |  |  | 0.03 |
| Yes | 25 | -1.56 (-2.27, -0.85) | <0.001 | 466.83 | <0.001 | 94.4 |  |
| No | 17 | -0.63 (-1.09, -0.17) | 0.007 | 83.79 | <0.001 | 79.7 |  |
| **Calorie restriction** |  |  |  |  |  |  | 0.52 |
| Yes | 29 | -1.25 (-1.79, -0.71) | <0.001 | 507.85 | <0.001 | 94.3 |  |
| No | 13 | -0.86 (-1.93, 0.21) | 0.11 | 134.15 | <0.001 | 89.6 |  |
| **Dietary report** |  |  |  |  |  |  | 0.79 |
| Self-reported | 27 | -1.08 (-1.59, -0.58) | <0.001 | 225.07 | <0.001 | 87.6 |  |
| Prescribed | 15 | -1.23 (-2.21, -0.25) | 0.01 | 401.02 | <0.001 | 96.3 |  |
| **Control diet** |  |  |  |  |  |  | 0.66 |
| Low fat | 27 | -1.24 (-1.75, -0.73) | <0.001 | 265.70 | <0.001 | 89.5 |  |
| Usual diet | 15 | -0.98 (-1.99, 0.03) | 0.06 | 344.41 | <0.001 | 95.6 |  |
| **Protein (%)** |  |  |  |  |  |  | 0.09 |
| <20 | 8 | -0.27 (-1.10, 0.57) | 0.53 | 21.45 | 0.006 | 62.7 |  |
| 20-24.9 | 10 | -1.06 (-2.08, -0.04) | 0.04 | 260.29 | <0.001 | 96.2 |  |
| ≥ 25 | 24 | -1.47 (-2.16, -0.79) | <0.001 | 348.02 | <0.001 | 93.1 |  |
| **Risk of bias** |  |  |  |  |  |  | 0.81 |
| High | 24 | -0.99 (-1.42, -0.55) | <0.001 | 146.74 | <0.001 | 83.6 |  |
| Some concerns | 11 | -1.33 (-2.52, -0.14) | 0.03 | 365.34 | <0.001 | 96.7 |  |
| Low | 7 | -0.90 (-1.47, -0.32) | 0.002 | 6.57 | 0.36 | 8.70 |  |

| **Table S10**. Assessment of credibility of subgroup difference for the effect of carbohydrate restriction (10% decrease) on body weight (kg) at 12-month follow-up based on ICEMAN. | | | | | | | | | |
| --- | --- | --- | --- | --- | --- | --- | --- | --- | --- |
| Variable | Q1 | Q2 | Q3 | Q4 | Q5 | Q6 | Q7 | Q8 | Overall credibility |
| Study design | Completely between | Definitely not similar | NA | Probably no | NA | Definitely yes | Definitely yes | Probably yes | Low |
| Sex | Completely between | Definitely not similar | Very small | Probably no | Chance a very likely explanation | Definitely yes | Definitely yes | Probably yes | Low |
| Metabolic status | Completely between | Definitely not similar | Definitely yes (very large) | Probably no | Chance a very likely explanation | Definitely yes | Definitely yes | Probably yes | Low |
| Exercise | Completely between | Definitely not similar | Definitely yes (very large) | Definitely yes | Chance a likely explanation or unclear | Definitely yes | Definitely yes | Probably yes | Low |
| Calorie restriction | Completely between | Definitely not similar | Definitely yes (very large) | Definitely yes | Chance a very likely explanation | Definitely yes | Definitely yes | Probably yes | Low |
| Dietary report | Completely between | Definitely not similar | Definitely yes (very large) | Probably no | Chance a very likely explanation | Definitely yes | Definitely yes | Probably yes | Low |
| Control diet | Completely between | Definitely not similar | Definitely yes (very large) | Probably no | Chance a very likely explanation | Definitely yes | Definitely yes | Probably yes | Low |
| Protein (%) | Completely between | Definitely not similar | Rather large | Definitely yes | Chance a very likely explanation | Definitely yes | Definitely yes | Probably yes | Low |
| Risk of bias | Completely between | Definitely not similar | Rather large | Probably no | Chance a very likely explanation | Definitely yes | Definitely yes | Probably yes | Low |
| **Abbreviations**: Q, question.  Q1, Is the analysis of effect modification based on comparison within rather than between trials? Q2, For within-trial comparisons, is the effect modification similar from trial to trial? Q3, For between-trial comparisons, is the number of trials large? Q4, Was the direction of the effect modification correctly hypothesized priori? Q5, Does a test for interaction suggest that chance is an unlikely explanation of the apparent effect modification? Q6, Did the authors test only a small number of effect modifiers? Q7, Did the authors use a random effects model? Q8, If the effect modifier is a continuous variable, were arbitrary cut points avoided? | | | | | | | | | |

| Table S11- Subgroup analysis of the effect of carbohydrate restriction (10% decrease) on body weight (kg) for longer than 12 months follow-up | | | | | | | |
| --- | --- | --- | --- | --- | --- | --- | --- |
| Study group | No. of studies | Meta-analysis | | Heterogeneity | | | |
|  |  | Mean difference (95% CI) | P value | Q statistic | P within group | I^2^ (%) | P between group |
| **Overall** | 9 | -0.87 (-1.81, 0.08) | 0.07 | 100.60 | <0.001 | 92 |  |
| **Study design** |  |  |  |  |  |  | - |
| Parallel | 9 | -0.87 (-1.81, 0.08) | 0.07 | 100.60 | <0.001 | 92 |  |
| Cross-over | - | - | - | - | - | - |  |
| **Sex** |  |  |  |  |  |  | 0.61 |
| Male | - | - | - | - | - | - |  |
| Female | 2 | -0.60 (-1.58, 0.38) | 0.23 | 0.77 | 0.38 | 0 |  |
| **Health status** |  |  |  |  |  |  | 0.85 |
| Healthy | 6 | -0.88 (-2.39, 0.64) | 0.26 | 90.92 | <0.001 | 94.5 |  |
| Unhealthy | 3 | -0.72 (-1.45, 0.01) | 0.053 | 4.55 | 0.10 | 56 |  |
| **Exercise** |  |  |  |  |  |  | 0.05 |
| Yes | 4 | -0.04 (-0.53, 0.45) | 0.88 | 5.97 | 0.11 | 49.8 |  |
| No | 5 | -1.50 (-2.86, -0.13) | 0.03 | 33.50 | <0.001 | 88.1 |  |
| **Calorie restriction** |  |  |  |  |  |  | 0.68 |
| Yes | 4 | -1.15 (-2.87, 0.57) | 0.19 | 90.92 | <0.001 | 96.7 |  |
| No | 5 | -0.77 (-1.24, -0.30) | 0.001 | 2.01 | 0.73 | 0 |  |
| **Dietary report** |  |  |  |  |  |  | 0.67 |
| Self-reported | 4 | -0.74 (-1.30, -0.18) | 0.009 | 1.63 | 0.65 | 0 |  |
| Prescribed | 5 | -1.07 (-2.46, 0.32) | 0.13 | 94.36 | <0.001 | 95.8 |  |
| **Control diet** |  |  |  |  |  |  | 0.46 |
| Low fat | 6 | -0.60 (-0.97, -0.17) | 0.005 | 4.60 | 0.47 | 0 |  |
| Usual diet | 3 | -1.53 (-4.06, 1.01) | 0.24 | 90.92 | <0.001 | 97.8 |  |
| **Protein (%)** |  |  |  |  |  |  | 0.24 |
| 20-24.9 | 5 | -0.32 (-1.06, 0.43) | 0.40 | 12.05 | 0.02 | 66.8 |  |
| ≥ 25 | 4 | -1.42 (-3.10, 0.25) | 0.10 | 47.42 | <0.001 | 93.7 |  |
| **Risk of bias** |  |  |  |  |  |  | 0.42 |
| High | 4 | -0.41 (-1.23, 0.42) | 0.33 | 14.37 | 0.002 | 79.1 |  |
| Some concerns | 5 | -1.17 (-2.83, 0.48) | 0.16 | 48.88 | <0.001 | 91.8 |  |

| **Table S12**. Assessment of credibility of subgroup difference for the effect of carbohydrate restriction (10% decreased) on body weight (kg) for longer than 12 months follow-up based on ICEMAN. | | | | | | | | | |
| --- | --- | --- | --- | --- | --- | --- | --- | --- | --- |
| Variable | Q1 | Q2 | Q3 | Q4 | Q5 | Q6 | Q7 | Q8 | Overall credibility |
| Study design | Completely between | Definitely not similar | NA | Probably no | NA | Definitely yes | Definitely yes | Probably yes | Low |
| Sex | Completely between | Definitely not similar | Very small | Probably no | Chance a very likely explanation | Definitely yes | Definitely yes | Probably yes | Low |
| Metabolic status | Completely between | Definitely not similar | Rather small | Probably no | Chance a very likely explanation | Definitely yes | Definitely yes | Probably yes | Low |
| Exercise | Completely between | Definitely not similar | Rather small | Definitely yes | Chance a very likely explanation | Definitely yes | Definitely yes | Probably yes | Low |
| Calorie restriction | Completely between | Definitely not similar | Rather small | Definitely yes | Chance a very likely explanation | Definitely yes | Definitely yes | Probably yes | Low |
| Dietary report | Completely between | Definitely not similar | Rather small | Probably no | Chance a very likely explanation | Definitely yes | Definitely yes | Probably yes | Low |
| Control diet | Completely between | Definitely not similar | Rather small | Probably no | Chance a very likely explanation | Definitely yes | Definitely yes | Probably yes | Low |
| Protein (%) | Completely between | Definitely not similar | Rather small | Definitely yes | Chance a very likely explanation | Definitely yes | Definitely yes | Probably yes | Low |
| Risk of bias | Completely between | Definitely not similar | Rather small | Probably no | Chance a very likely explanation | Definitely yes | Definitely yes | Probably yes | Low |
| **Abbreviations**: Q, question.  Q1, Is the analysis of effect modification based on comparison within rather than between trials? Q2, For within-trial comparisons, is the effect modification similar from trial to trial? Q3, For between-trial comparisons, is the number of trials large? Q4, Was the direction of the effect modification correctly hypothesized priori? Q5, Does a test for interaction suggest that chance is an unlikely explanation of the apparent effect modification? Q6, Did the authors test only a small number of effect modifiers? Q7, Did the authors use a random effects model? Q8, If the effect modifier is a continuous variable, were arbitrary cut points avoided? | | | | | | | | | |

Table S13- GRADE evidence table for the effect of carbohydrate restriction on body weight at 6-month, 12-month, and longer than 12 months follow-ups.

| **Certainty assessment** | | | | | | **Effect** | **Certainty** | **Importance** |
| --- | --- | --- | --- | --- | --- | --- | --- | --- |
| **№ of studies (Num. participants)** | **Risk of bias** | **Inconsistency** | **Indirectness** | **Imprecision** | **Other considerations** | **Mean difference (95%CI) per 10% decrease in carbohydrate intake** |  |  |
| Body weight (up to 6 month) | | | | | | | | |
| 101 (4135) | not serious^a^ | serious^b^ | not serious | serious^c^ | dose response gradient | -0.64 (-0.79, -0.49) | Moderate | Critical |
| Body weight 6 to 12 month | | | | | | | | |
| 42 (2657) | not serious^d^ | very serious^e^ | not serious | not serious^f^ | dose response gradient | -1.15 (-1.61, -0.69) | Moderate | Critical |
| Body weight longer than 12 months | | | | | | | | |
| 9 (541) | very serious^g^ | very serious^h^ | not serious | serious^i^ | dose response gradient | -0.87 (-1.81, 0.08) | Very low | Critical |

#### Explanations

a. Most of studies (92 of 101) were rated to have some concerns and low risk of bias, however the direction and significance of effect sizes did not differ between overall analysis vs high quality trials (Effect size for good quality trails (MD= -0.83; 95% CI: -1.18, -0.48); n=9). Not downgraded. b. I^2^=80.5, P heterogeneity< 0.001. The predefined subgroups and sensitivity analysis did not explain the observed of heterogeneity. Downgraded. c. Effect estimate did not surpass MCID for body weight (4.4 kg), and 95%CI failed to exclude important benefit. Downgraded. d. Most of studies (35 of 42) were rated to have some concerns and low risk of bias, however the direction and significance of effect sizes did not differ between overall analysis vs high quality trials category (MD= -0.90; 95% CI: -1.47, -0.32); n=7). Not downgraded. e. I^2^=93.2, P heterogeneity< 0.001. The predefined subgroups and sensitivity analysis did not explain the observed of heterogeneity. Downgraded. f. Effect estimate (in the nonlinear dose-response meta-analysis) surpassed MCID threshold and 95%CI excluded important harm and benefit. Not downgraded. g. All of trials were rated to have some concerns and low risk of bias. Main trial limitations were lack of random sequence generation, allocation concealment, and personnel and blinding of outcome assessment. Downgraded h. I^2^=92, P heterogeneity< 0.001. The predefined subgroups and sensitivity analysis did not explain the source of heterogeneity. Downgraded. i. The sample size did not reach the optimal information size (N ≤ 800). Downgraded.

**Identification of studies via databases and registers**

Records removed *before screening*:

Duplicate records removed

(n =8288)

Records identified from*:

Databases (n = 20,803)

**Identification**

Records screened

(n =12,515)

Records excluded**

(n =11,989)

Reports sought for retrieval

(n =526)

Reports not retrieved

(n = 0)

**Screening**

Reports excluded (n =416):

Additional intervention (n=11)

Didn’t report the amount of carbohydrate (n=16)

Not relevant outcome (n=39)

Both groups received low CHO diet (n=14)

Case-report/ review/ commentary (n=17)

Conducted in children/ athlete/ pregnant (n=13)

Deviation from prescribe diet (n=2)

Intervention wasn't low CHO diet (n=44)

Mixed population (normal weight and obese) (n=131)

Not obese (n=16)

Observational study (n=9)

Short duration (n=12)

Sufficient data wasn't available (n=8)

Under Bypass surgery (n=4)

Without control group (n=29)

Repeated from same population (n=51)

Reports assessed for eligibility

(n =526)

Studies included in review

(n = 110)

**Included**

**Supplementary Figure 1-** Flow diagram of the study selection process.

**
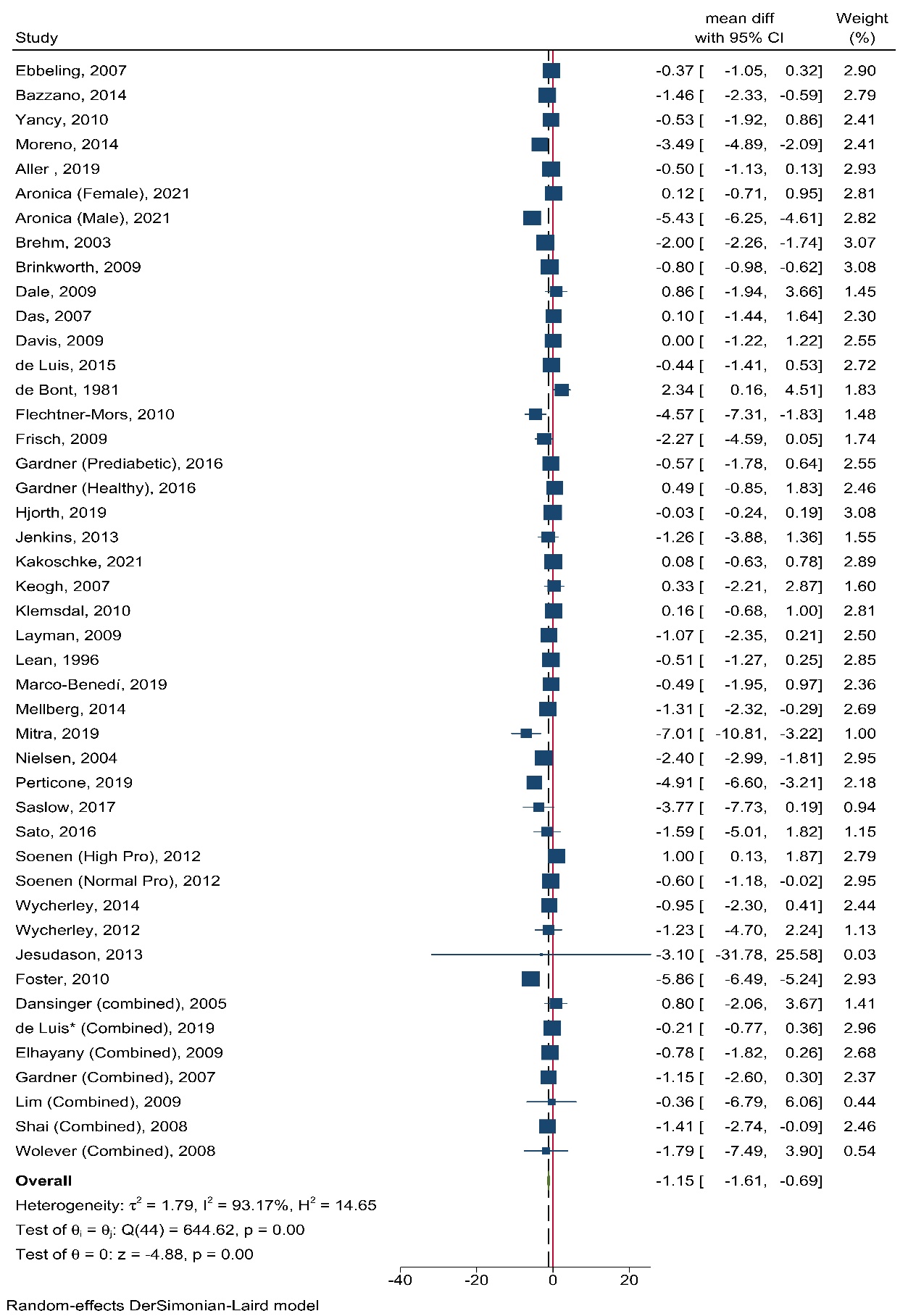
**

**Fig S2-** The effect of carbohydrate restriction (10% decrease) on body weight (kg) at 12-month follow-up.


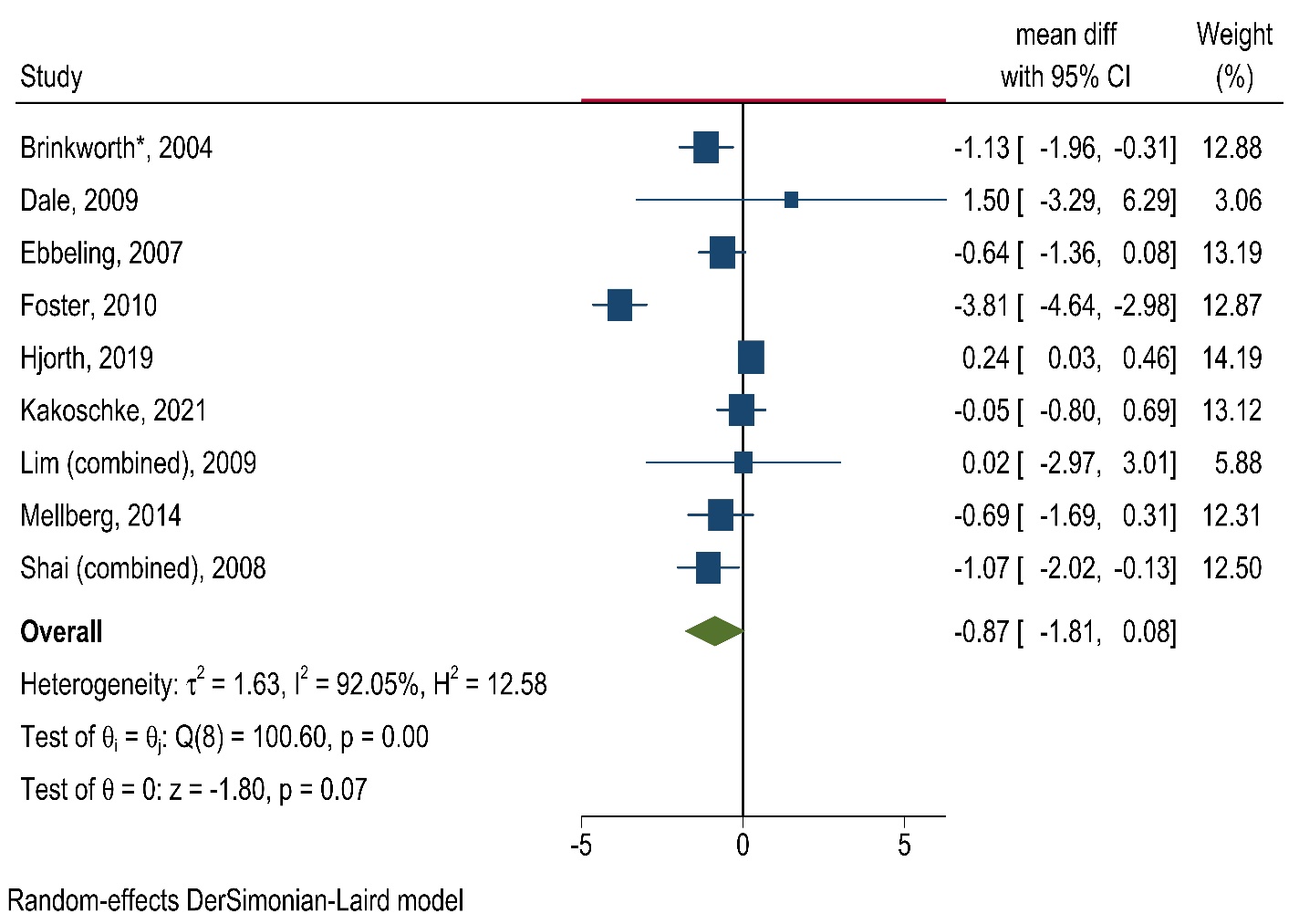
**Fig S3-** The effect of carbohydrate restriction (10% decrease) on body weight (kg) at follow-up longer than 12 months.


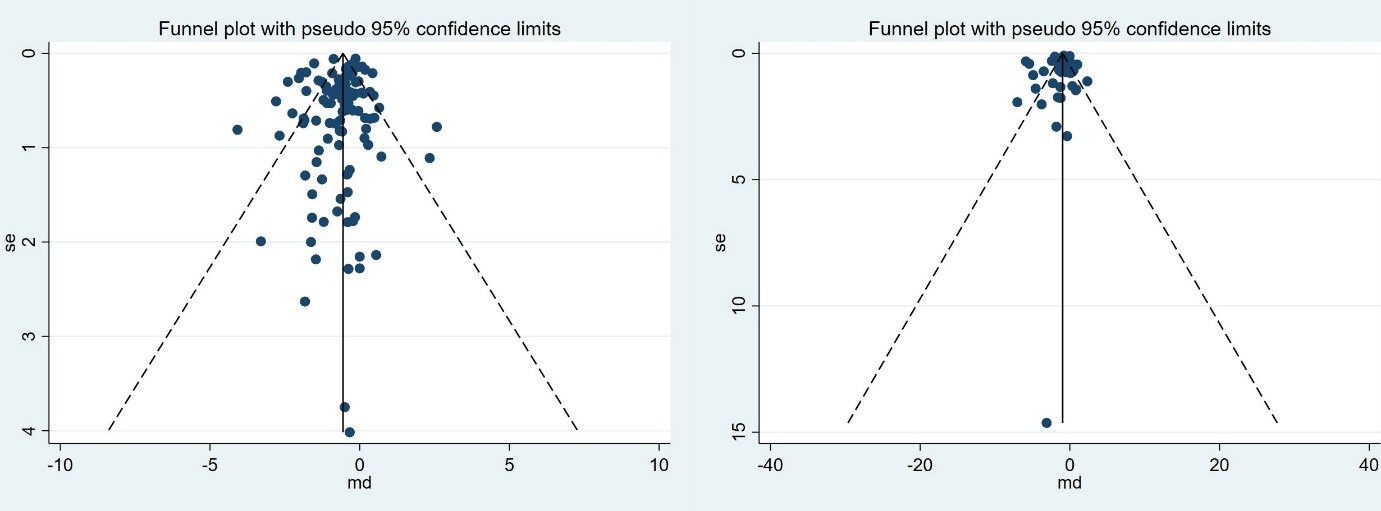


**Fig S4-** Funnel plot of the effect of carbohydrate restriction diet on body weight at 6- and 12-month follow-ups.

**References**:

1. Guyatt GH, Oxman AD, Vist GE, Kunz R, Falck-Ytter Y, Alonso-Coello P, et al. GRADE: an emerging consensus on rating quality of evidence and strength of recommendations. Bmj. 2008;336(7650):924-6.

2. Guyatt GH, Oxman AD, Vist G, Kunz R, Brozek J, Alonso-Coello P, et al. GRADE guidelines: 4. Rating the quality of evidence—study limitations (risk of bias). Journal of clinical epidemiology. 2011;64(4):407-15.

3. Guyatt GH, Oxman AD, Kunz R, Woodcock J, Brozek J, Helfand M, et al. GRADE guidelines: 7. Rating the quality of evidence—inconsistency. Journal of clinical epidemiology. 2011;64(12):1294-302.

4. Guyatt GH, Oxman AD, Kunz R, Woodcock J, Brozek J, Helfand M, et al. GRADE guidelines: 8. Rating the quality of evidence—indirectness. Journal of clinical epidemiology. 2011;64(12):1303-10.

5. Guyatt GH, Oxman AD, Kunz R, Brozek J, Alonso-Coello P, Rind D, et al. GRADE guidelines 6. Rating the quality of evidence—imprecision. Journal of clinical epidemiology. 2011;64(12):1283-93.

6. Guyatt GH, Oxman AD, Montori V, Vist G, Kunz R, Brozek J, et al. GRADE guidelines: 5. Rating the quality of evidence—publication bias. Journal of clinical epidemiology. 2011;64(12):1277-82.

7. de Luis D, Domingo JC, Izaola O, Casanueva FF, Bellido D, Sajoux I. Effect of DHA supplementation in a very low-calorie ketogenic diet in the treatment of obesity: a randomized clinical trial. Endocrine. 2016;54(1):111-22.

8. Dicker D, Beck A, Markel A, Marcovicu D, Mazzawi S, Sarid M, et al. Weight Loss, Dietary Preferences, and Reduction in the Sense of Smell with the Use of a Novel Nasal Device. Obesity facts. 2020;13(5):473-86.

9. Facchini FS, Saylor KL. A low-iron-available, polyphenol-enriched, carbohydrate-restricted diet to slow progression of diabetic nephropathy. Diabetes. 2003;52(5):1204-9.

10. Gutiérrez‐Repiso C, Hernández‐García C, García‐Almeida JM, Bellido D, Martín‐Núñez GM, Sánchez‐Alcoholado L, et al. Effect of Synbiotic Supplementation in a Very‐Low‐Calorie Ketogenic Diet on Weight Loss Achievement and Gut Microbiota: A Randomized Controlled Pilot Study. Mol Nutr Food Res. 2019;63(19):1900167.

11. Jenkins DJ, Kendall CW, Vuksan V, Faulkner D, Augustin LS, Mitchell S, et al. Effect of lowering the glycemic load with canola oil on glycemic control and cardiovascular risk factors: a randomized controlled trial. Diabetes Care. 2014;37(7):1806-14.

12. Kanikowska D, Kanikowska A, Swora-Cwynar E, Grzymisławski M, Sato M, Bręborowicz A, et al. Moderate caloric restriction partially improved oxidative stress markers in obese humans. Antioxidants. 2021;10(7):1018.

13. Marnani EH, Ghadiri-Anari A, Ramezani-Jolfaie N, Mohammadi M, Namayandeh SM, Mozaffari-Khosravi H, et al. Effect of fennel supplementation along with high-protein, low-carbohydrate weight-loss diet on insulin resistance and percentage of fat and muscle mass in overweight/obese women with polycystic ovary syndrome. J Funct Foods. 2020;67:103848.

14. Mastaloudis A, Sheth C, Hester SN, Wood SM, Prescot A, McGlade E, et al. Supplementation with a putative calorie restriction mimetic micronutrient blend increases glutathione concentrations and improves neuroenergetics in brain of healthy middle-aged men and women. Free Radic Biol Med. 2020;153:112-21.

15. Nadjarzadeh A, Ghadiri-Anari A, Ramezani-Jolfaie N, Mohammadi M, Salehi-Abargouei A, Namayande SM, et al. Effect of hypocaloric high-protein, low-carbohydrate diet supplemented with fennel on androgenic and anthropometric indices in overweight and obese women with polycystic ovary syndrome: A randomized placebo-controlled trial. Complement Ther Med. 2021;56:102633.

16. Ren M, Zhang H, Qi J, Hu A, Jiang Q, Hou Y, et al. An almond-based low carbohydrate diet improves depression and glycometabolism in patients with Type 2 Diabetes through modulating gut microbiota and GLP-1: A randomized controlled trial. Nutrients. 2020;12(10):3036.

17. Vaghef-Mehrabany E, Ranjbar F, Asghari-Jafarabadi M, Hosseinpour-Arjmand S, Ebrahimi-Mameghani M. Calorie restriction in combination with prebiotic supplementation in obese women with depression: Effects on metabolic and clinical response. Nutr Neurosci. 2021;24(5):339-53.

18. Ahmed SR, Bellamkonda S, Zilbermint M, Wang J, Kalyani RR. Effects of the low carbohydrate, high fat diet on glycemic control and body weight in patients with type 2 diabetes: experience from a community-based cohort. BMJ Open Diabetes Research and Care. 2020;8(1):e000980.

19. Beavers KM, Nesbit BA, Kiel JR, Sheedy JL, Arterburn LM, Collins AE, et al. Effect of an energy-restricted, nutritionally complete, higher protein meal plan on body composition and mobility in older adults with obesity: a randomized controlled trial. The Journals of Gerontology: Series A. 2019;74(6):929-35.

20. Perissiou M, Borkoles E, Kobayashi K, Polman R. The effect of an 8 week prescribed exercise and low-carbohydrate diet on cardiorespiratory fitness, body composition and cardiometabolic risk factors in obese individuals: A randomised controlled trial. Nutrients. 2020;12(2):482.

21. Röhling M, Martin K, Ellinger S, Schreiber M, Martin S, Kempf K. Weight Reduction by the Low-Insulin-Method—A Randomized Controlled Trial. Nutrients. 2020;12(10):3004.

22. Haufe S, Engeli S, Kast P, Böhnke J, Utz W, Haas V, et al. Randomized comparison of reduced fat and reduced carbohydrate hypocaloric diets on intrahepatic fat in overweight and obese human subjects. Hepatology. 2011;53(5):1504-14.

23. Hussain TA, Mathew TC, Dashti AA, Asfar S, Al-Zaid N, Dashti HM. Effect of low-calorie versus low-carbohydrate ketogenic diet in type 2 diabetes. Nutrition. 2012;28(10):1016-21.

24. Morris E, Aveyard P, Dyson P, Noreik M, Bailey C, Fox R, et al. A food‐based, low‐energy, low‐carbohydrate diet for people with type 2 diabetes in primary care: A randomized controlled feasibility trial. Diabetes, Obesity and Metabolism. 2020;22(4):512-20.

25. Phillips SA, Jurva JW, Syed AQ, Syed AQ, Kulinski JP, Pleuss J, et al. Benefit of low-fat over low-carbohydrate diet on endothelial health in obesity. Hypertension. 2008;51(2):376-82.

26. Röhling M, Kempf K, Banzer W, Berg A, Braumann K-M, Tan S, et al. Prediabetes Conversion to Normoglycemia Is Superior Adding a Low-Carbohydrate and Energy Deficit Formula Diet to Lifestyle Intervention—A 12-Month Subanalysis of the ACOORH Trial. Nutrients. 2020;12(7):2022.

27. Torbay N, Baba NH, Sawaya S, Bajjani R, Habbal Z, Azar S, et al. High protein vs high carbohydrate hypoenergetic diet in treatment of obese normoinsulinemic and hyperinsulinemic subjects. Nutr Res. 2002;22(5):587-98.

28. Tsai AG, Glick HA, Shera D, Stern L, Samaha FF. Cost‐effectiveness of a low‐carbohydrate diet and a standard diet in severe obesity. Obes Res. 2005;13(10):1834-40.

29. Vander Wal JS, Mcburney MI, Moellering N, Marth J, Dhurandhar NV. Moderate-carbohydrate low-fat versus low-carbohydrate high-fat meal replacements for weight loss. Int J Food Sci Nutr. 2007;58(4):321-9.

30. Voils CI, Pendergast J, Hale SL, Gierisch JM, Strawbridge EM, Levine E, et al. A randomized feasibility pilot trial of a financial incentives intervention for dietary self-monitoring and weight loss in adults with obesity. Transl Behav Med. 2021;11(4):954-69.

31. Due A, Toubro S, Skov A, Astrup A. Effect of normal-fat diets, either medium or high in protein, on body weight in overweight subjects: a randomised 1-year trial. Int J Obes. 2004;28(10):1283-90.

32. Esposito K, Maiorino MI, Ciotola M, Di Palo C, Scognamiglio P, Gicchino M, et al. Effects of a Mediterranean-style diet on the need for antihyperglycemic drug therapy in patients with newly diagnosed type 2 diabetes: a randomized trial. Ann Intern Med. 2009;151(5):306-14.

33. Pedersen LR, Olsen RH, Anholm C, Astrup A, Eugen-Olsen J, Fenger M, et al. Effects of 1 year of exercise training versus combined exercise training and weight loss on body composition, low-grade inflammation and lipids in overweight patients with coronary artery disease: a randomized trial. Cardiovasc Diabetol. 2019;18(1):1-13.

34. Ahmed HO, Ezzat RF. Quality of life of obese patients after treatment with the insertion of intra-gastric balloon versus Atkins diet in Sulaimani Governorate, Kurdistan Region, Iraq. Annals of Medicine and Surgery. 2019;37:42-6.

35. Anguah KO-B, Syed-Abdul MM, Hu Q, Jacome-Sosa M, Heimowitz C, Cox V, et al. Changes in food cravings and eating behavior after a dietary carbohydrate restriction intervention trial. Nutrients. 2020;12(1):52.

36. Athinarayanan SJ, Hallberg SJ, McKenzie AL, Lechner K, King S, McCarter JP, et al. Impact of a 2-year trial of nutritional ketosis on indices of cardiovascular disease risk in patients with type 2 diabetes. Cardiovasc Diabetol. 2020;19(1):1-13.

37. Batar N, Kermen S, Sevdin S, Ersin A, San S, Erdem MG, et al. Effect of Pilates on body composition and some biochemical parameters of women with type 2 diabetes on a low-carbohydrate or high-complex-carbohydrate diabetic diet. 2020.

38. Bhoite R, Chandrasekaran A, Pratti VL, Satyavrat V, Aacharya S, Mane A, et al. Effect of a High-Protein High-Fibre Nutritional Supplement on Lipid Profile in Overweight/Obese Adults with Type 2 Diabetes Mellitus: A 24-Week Randomized Controlled Trial. J Nutr Metab. 2021;2021.

39. Bladbjerg E-M, Larsen TM, Due A, Stender S, Astrup A, Jespersen J. Effects on markers of inflammation and endothelial cell function of three ad libitum diets differing in type and amount of fat and carbohydrate: a 6-month randomised study in obese individuals. Br J Nutr. 2011;106(1):123-9.

40. Breukelman GJ, Basson AK, Djarova TG, Du Preez CJ, Shaw I, Shaw BS. Combination low carbohydrate, high fat diet and physical activity intervention on lipoprotein-lipids in type 2 diabetics. Asian J Sports Med. 2019;10(4).

41. Chen J, Huang Y, Xie H, Bai H, Lin G, Dong Y, et al. Impact of a low-carbohydrate and high-fiber diet on nonalcoholic fatty liver disease. Asia Pac J Clin Nutr. 2020;29(3):483-90.

42. Crujeiras AB, Izquierdo AG, Primo D, Milagro FI, Sajoux I, Jácome A, et al. Epigenetic landscape in blood leukocytes following ketosis and weight loss induced by a very low calorie ketogenic diet (VLCKD) in patients with obesity. Clin Nutr. 2021.

43. Di Lorenzo C, Pinto A, Ienca R, Coppola G, Sirianni G, Di Lorenzo G, et al. A randomized double-blind, cross-over trial of very low-calorie diet in overweight migraine patients: a possible role for ketones? Nutrients. 2019;11(8):1742.

44. Dong TS, Luu K, Lagishetty V, Sedighian F, Woo S-L, Dreskin BW, et al. A high protein calorie restriction diet alters the gut microbiome in obesity. Nutrients. 2020;12(10):3221.

45. Drummen M, Tischmann L, Gatta-Cherifi B, Fogelholm M, Raben A, Adam TC, et al. High compared with moderate protein intake reduces adaptive thermogenesis and induces a negative energy balance during long-term weight-loss maintenance in participants with prediabetes in the postobese state: a PREVIEW study. The Journal of nutrition. 2020;150(3):458-63.

46. Ebbeling CB, Bielak L, Lakin PR, Klein GL, Wong JM, Luoto PK, et al. Energy requirement is higher during weight-loss maintenance in adults consuming a low-compared with high-carbohydrate diet. The Journal of nutrition. 2020;150(8):2009-15.

47. Feng R, Sun G, Zhang Y, Sun Q, Ju L, Sun C, et al. Short‐term high‐fat diet exacerbates insulin resistance and glycolipid metabolism disorders in young obese men with hyperlipidemia, as determined by metabolomics analysis using ultra‐HPLC–quadrupole time‐of‐flight mass spectrometry. J Diabetes. 2019;11(2):148-60.

48. Foraker RE, Pennell M, Sprangers P, Vitolins MZ, DeGraffinreid C, Paskett ED. Effect of a low-fat or low-carbohydrate weight-loss diet on markers of cardiovascular risk among premenopausal women: a randomized trial. J Womens Health. 2014;23(8):675-80.

49. Hall KD, Guo J, Chen KY, Leibel RL, Reitman ML, Rosenbaum M, et al. Methodologic considerations for measuring energy expenditure differences between diets varying in carbohydrate using the doubly labeled water method. The American journal of clinical nutrition. 2019;109(5):1328-34.

50. He Y, Schofield J, Mahling P, Mendonza AE, Hinder M. Investigation and management of stool frequency and consistency associated with SGLT1 inhibition by reducing dietary carbohydrate: a randomized trial. Clin Pharmacol Ther. 2020;108(5):995-1002.

51. Holsen LM, Hoge WS, Lennerz BS, Cerit H, Hye T, Moondra P, et al. Diets Varying in Carbohydrate Content Differentially Alter Brain Activity in Homeostatic and Reward Regions in Adults. The Journal of Nutrition. 2021.

52. Hoover SE, Il'yasova D, Fontaine KR, Spasojevic I, Gower BA, Goss AM. A Pilot Study of Associations Between Visceral Fat, IL-6, and Urinary F2-Isoprostanes in Older Adults Exposed to a Diet Intervention. Current Developments in Nutrition. 2021.

53. Hudson JL, Zhou J, Campbell WW. Adults who are overweight or obese and consuming an energy-restricted healthy US-style eating pattern at either the recommended or a higher protein quantity perceive a shift from “High” to “Good” sleep: A randomized controlled trial. The Journal of Nutrition. 2020;150(12):3216-23.

54. Hyde PN, Sapper TN, Crabtree CD, LaFountain RA, Bowling ML, Buga A, et al. Dietary carbohydrate restriction improves metabolic syndrome independent of weight loss. JCI insight. 2019;4(12).

55. Kitabchi AE, McDaniel KA, Wan JY, Tylavsky FA, Jacovino CA, Sands CW, et al. Effects of high-protein versus high-carbohydrate diets on markers of β-cell function, oxidative stress, lipid peroxidation, proinflammatory cytokines, and adipokines in obese, premenopausal women without diabetes: a randomized controlled trial. Diabetes Care. 2013;36(7):1919-25.

56. Koelman L, Markova M, Seebeck N, Hornemann S, Rosenthal A, Lange V, et al. Effects of High and Low Protein Diets on Inflammatory Profiles in People with Morbid Obesity: A 3-Week Intervention Study. Nutrients. 2020;12(12):3636.

57. Kong Z, Hu M, Liu Y, Shi Q, Zou L, Sun S, et al. Affective and enjoyment responses to short-term high-intensity interval training with low-carbohydrate diet in overweight young women. Nutrients. 2020;12(2):442.

58. Kotopoulea-Nikolaidi M, Watkins E, Giannopoulou I. Effects of High Carbohydrate vs. High Protein Pre-exercise Feedings on Psychophysiological Responses to High Intensity Interval Exercise in Overweight Perimenopausal Women. Frontiers in nutrition. 2019;5:141.

59. Li X, Sun D, Zhou T, Ma H, Heianza Y, Liang Z, et al. Changes of Branched-Chain Amino Acids and Ectopic Fat in Response to Weight-loss Diets: the POUNDS Lost Trial. The Journal of Clinical Endocrinology & Metabolism. 2020;105(10):e3747-e56.

60. Malik N, Tonstad S, Paalani M, Dos Santos H, Luiz do Prado W. Are long‐term FAD diets restricting micronutrient intake? A randomized controlled trial. Food Science & Nutrition. 2020;8(11):6047-60.

61. McKenna CF, Salvador AF, Hughes RL, Scaroni SE, Alamilla RA, Askow AT, et al. Higher protein intake during resistance training does not potentiate strength, but modulates gut microbiota, in middle-aged adults: a randomized control trial. American Journal of Physiology-Endocrinology and Metabolism. 2021;320(5):E900-E13.

62. Michalczyk MM, Klonek G, Maszczyk A, Zajac A. The effects of a low calorie ketogenic diet on glycaemic control variables in hyperinsulinemic overweight/obese females. Nutrients. 2020;12(6):1854.

63. Nuttall FQ, Almokayyad RM, Gannon MC. Circulating lipids in men with type 2 diabetes following 3 days on a carbohydrate‐free diet versus 3 days of fasting. Physiological Reports. 2020;8(19):e14569.

64. Saslow LR, Moskowitz JT, Mason AE, Daubenmier J, Liestenfeltz B, Missel AL, et al. Intervention Enhancement Strategies Among Adults With Type 2 Diabetes in a Very Low–Carbohydrate Web-Based Program: Evaluating the Impact With a Randomized Trial. JMIR diabetes. 2020;5(3):e15835.

65. Schroor MM, Plat J, Konings MC, Smeets ET, Mensink RP. Effect of dietary macronutrients on intestinal cholesterol absorption and endogenous cholesterol synthesis: a randomized crossover trial. Nutrition, Metabolism and Cardiovascular Diseases. 2021;31(5):1579-85.

66. Shimy KJ, Feldman HA, Klein GL, Bielak L, Ebbeling CB, Ludwig DS. Effects of dietary carbohydrate content on circulating metabolic fuel availability in the postprandial state. Journal of the Endocrine Society. 2020;4(7):bvaa062.

67. Sun D, Zhou T, Li X, Heianza Y, Liang Z, Bray GA, et al. Genetic susceptibility, dietary protein intake, and changes of blood pressure: the pounds lost trial. Hypertension. 2019;74(6):1460-7.

68. Tay J, Thompson CH, Luscombe-Marsh ND, Noakes M, Buckley JD, Wittert GA, et al. Nutritional adequacy of very low-and high-carbohydrate, low saturated fat diets in adults with type 2 diabetes: A secondary analysis of a 2-year randomised controlled trial. Diabetes Res Clin Pract. 2020;170:108501.

69. Thomsen MN, Skytte MJ, Astrup A, Deacon CF, Holst JJ, Madsbad S, et al. The clinical effects of a carbohydrate-reduced high-protein diet on glycaemic variability in metformin-treated patients with type 2 diabetes mellitus: A randomised controlled study. Clinical Nutrition ESPEN. 2020;39:46-52.

70. Tischmann L, Drummen M, Joris PJ, Gatta-Cherifi B, Raben A, Fogelholm M, et al. Effects of a High-Protein Diet on Cardiometabolic Health, Vascular Function, and Endocannabinoids—A PREVIEW Study. Nutrients. 2020;12(5):1512.

71. Zhou T, Heianza Y, Chen Y, Li X, Sun D, DiDonato JA, et al. Circulating gut microbiota metabolite trimethylamine N-oxide (TMAO) and changes in bone density in response to weight loss diets: The Pounds lost trial. Diabetes Care. 2019;42(8):1365-71.

72. Žlibinaitė L, Solianik R, Vizbaraitė D, Mickevičienė D, Skurvydas A. The effect of combined aerobic exercise and calorie restriction on mood, cognition, and motor behavior in overweight and obese women. Journal of Physical Activity and Health. 2020;17(2):204-10.

73. Alvarado-Reynoso B, Ambriz-Tututi M. Effects of repetitive transcranial magnetic stimulation in combination with a low-carbohydrate diet in overweight or obese patients. A randomized controlled trial. Obesity Medicine. 2019;14:100095.

74. Colica C, Merra G, Gasbarrini A, De Lorenzo A, Cioccoloni G, Gualtieri P, et al. Efficacy and safety of very-low-calorie ketogenic diet: a double blind randomized crossover study. Eur Rev Med Pharmacol Sci. 2017;21(9):2274-89.

75. Englert I, Bosy-Westphal A, Bischoff SC, Kohlenberg-Müller K. Impact of Protein Intake during Weight Loss on Preservation of Fat-Free Mass, Resting Energy Expenditure, and Physical Function in Overweight Postmenopausal Women: A Randomized Controlled Trial. Obesity Facts. 2021;14(3):259-70.

76. Hwang C-L, Ranieri C, Szczurek MR, Ellythy AM, Elokda A, Mahmoud AM, et al. The effect of low-carbohydrate diet on macrovascular and microvascular endothelial function is not affected by the provision of caloric restriction in women with obesity: a randomized study. Nutrients. 2020;12(6):1649.

77. Iqbal N, Vetter ML, Moore RH, Chittams JL, Dalton‐Bakes CV, Dowd M, et al. Effects of a low‐intensity intervention that prescribed a low‐carbohydrate vs. a low‐fat diet in obese, diabetic participants. Obesity. 2010;18(9):1733-8.

78. Johnston CS, Tjonn SL, Swan PD, White A, Hutchins H, Sears B. Ketogenic low-carbohydrate diets have no metabolic advantage over nonketogenic low-carbohydrate diets. The American journal of clinical nutrition. 2006;83(5):1055-61.

79. Johnstone AM, Horgan GW, Murison SD, Bremner DM, Lobley GE. Effects of a high-protein ketogenic diet on hunger, appetite, and weight loss in obese men feeding ad libitum. The American journal of clinical nutrition. 2008;87(1):44-55.

80. McAuley K, Hopkins C, Smith K, McLay R, Williams S, Taylor R, et al. Comparison of high-fat and high-protein diets with a high-carbohydrate diet in insulin-resistant obese women. Diabetologia. 2005;48(1):8-16.

81. McAuley K, Smith K, Taylor R, McLay R, Williams S, Mann J. Long-term effects of popular dietary approaches on weight loss and features of insulin resistance. Int J Obes. 2006;30(2):342-9.

82. Merra G, Miranda R, Barrucco S, Gualtieri P, Mazza M, Moriconi E, et al. Very-low-calorie ketogenic diet with aminoacid supplement versus very low restricted-calorie diet for preserving muscle mass during weight loss: a pilot double-blind study. Eur Rev Med Pharmacol Sci. 2016;20(12):2613-21.

83. Sajoux I, Lorenzo PM, Gomez-Arbelaez D, Zulet M, Abete I, Castro AI, et al. Effect of a very-low-calorie ketogenic diet on circulating myokine levels compared with the effect of bariatric surgery or a low-calorie diet in patients with obesity. Nutrients. 2019;11(10):2368.

84. Saslow LR, Mason AE, Kim S, Goldman V, Ploutz-Snyder R, Bayandorian H, et al. An online intervention comparing a very low-carbohydrate ketogenic diet and lifestyle recommendations versus a plate method diet in overweight individuals with type 2 diabetes: a randomized controlled trial. J Med Internet Res. 2017;19(2):e5806.

85. Sun S, Kong Z, Shi Q, Zhang H, Lei O-K, Nie J, editors. Carbohydrate Restriction with or without Exercise Training Improves Blood Pressure and Insulin Sensitivity in Overweight Women. Healthcare; 2021: Multidisciplinary Digital Publishing Institute.

86. Taylor PJ, Thompson CH, Luscombe-Marsh ND, Wycherley TP, Wittert G, Brinkworth GD. Efficacy of real-time continuous glucose monitoring to improve effects of a prescriptive lifestyle intervention in type 2 diabetes: a pilot study. Diabetes Ther. 2019;10(2):509-22.

87. Alexander J, Nagi D. Severe ketoacidosis in a non-diabetic lactating woman on a ketogenic diet. British Journal of Diabetes. 2020;20(2):145-6.

88. Paola L, Monaco L. ROLE OF VERY LOW-CALORIE KETOGENIC DIET (VLCKD) IN NAFLD ANALYSIS OF A CLINICAL CASE. 2020.

89. Seyfried TN, Shivane AG, Kalamian M, Maroon JC, Mukherjee P, Zuccoli G. Ketogenic Metabolic Therapy, Without Chemo or Radiation, for the Long-Term Management of IDH1-Mutant Glioblastoma: An 80-Month Follow-Up Case Report. Frontiers in nutrition. 2021;8:281.

90. Similä ME, Auranen M, Piirilä PL. Beneficial effects of ketogenic diet on phosphofructokinase deficiency (glycogen storage disease type VII). Front Neurol. 2020;11:57.

91. Steinmetz-Wood S, Gilbert M, Menson K. A Case of diabetic ketoacidosis in a patient on an SGLT2 inhibitor and a ketogenic diet: a critical trio not to be missed. Case Reports in Endocrinology. 2020;2020.

92. Abrahim M. The forgotten confounding variable of the very-low-caloric intervention in the ketogenic diet. Endocrine. 2021:1-.

93. Goldenberg JZ, Johnston BC. Low and very low carbohydrate diets for diabetes remission. BMJ. 2021;373.

94. Langlais CS, Chan JM. Opportunities and challenges for research on low-carbohydrate diets in prostate cancer. Nature Reviews Urology. 2020;17(8):437-8.

95. Pfeiffer AF, Pedersen E, Schwab U, Risérus U, Aas A-M, Uusitupa M, et al. The effects of different quantities and qualities of protein intake in people with diabetes mellitus. Nutrients. 2020;12(2):365.

96. Ahmed A, Mateo-Garcia M, Arewa A, Caratella K. Integrated Performance Optimization of Higher Education Buildings Using Low-Energy Renovation Process and User Engagement. Energies. 2021;14(5):1475.

97. Katz JB, Owusu K, Nussbaum I, Beekman R, DeFilippo NA, Gilmore EJ, et al. Pearls and Pitfalls of Introducing Ketogenic Diet in Adult Status Epilepticus: A Practical Guide for the Intensivist. Journal of Clinical Medicine. 2021;10(4):881.

98. Kaviani M, Izadi A, Heshmati J. Would creatine supplementation augment exercise performance during a low carbohydrate high fat diet? Med Hypotheses. 2021;146:110369.

99. Keith L, Seo C, Rowsemitt C, Pfeffer M, Wahi M, Staggs M, et al. Ketogenic diet as a potential intervention for lipedema. Med Hypotheses. 2021;146:110435.

100. Ludwig DS. The ketogenic diet: evidence for optimism but high-quality research needed. The Journal of Nutrition. 2020;150(6):1354-9.

101. Moon J, Koh G. Clinical Evidence and Mechanisms of High-Protein Diet-Induced Weight Loss. Journal of Obesity & Metabolic Syndrome. 2020;29(3):166.

102. Moscatelli F, Valenzano A, Polito R, Francesco S, Montana A, Salerno M, et al. Ketogenic diet and sport performance. Sport Mont. 2020;18(1):91-4.

103. Pierpaolo T, Castellana M, Diego B, Casanueva FF. Confusion in the nomenclature of ketogenic diets blurs evidence. Rev Endocr Metab Disord. 2020;21(1):1-3.

104. Pauley M, Mays C, Bailes Jr JR, Schwartzman ML, Castle M, McCoy M, et al. Carbohydrate-Restricted Diet: A Successful Strategy for Short-Term Management in Youth with Severe Obesity—An Observational Study. Metab Syndr Relat Disord. 2021;19(5):281-7.

105. Greene DA, Varley BJ, Hartwig TB, Chapman P, Rigney M. A low-carbohydrate ketogenic diet reduces body mass without compromising performance in powerlifting and olympic weightlifting athletes. The Journal of Strength & Conditioning Research. 2018;32(12):3373-82.

106. Gregory RM, Hamdan H, Torisky D, Akers J. A low-carbohydrate ketogenic diet combined with 6-weeks of crossfit training improves body composition and performance. Int J Sports Exerc Med. 2017;3:54.

107. Kephart WC, Pledge CD, Roberson PA, Mumford PW, Romero MA, Mobley CB, et al. The three-month effects of a ketogenic diet on body composition, blood parameters, and performance metrics in CrossFit trainees: a pilot study. Sports. 2018;6(1):1.

108. Mujika I. Case study: Long-term low-carbohydrate, high-fat diet impairs performance and subjective well-being in a world-class vegetarian long-distance triathlete. Int J Sport Nutr Exerc Metab. 2019;29(3):339-44.

109. Paoli A, Cenci L, Pompei P, Sahin N, Bianco A, Neri M, et al. Effects of two months of very low carbohydrate ketogenic diet on body composition, muscle strength, muscle area, and blood parameters in competitive natural body builders. Nutrients. 2021;13(2):374.

110. Paoli A, Grimaldi K, D’Agostino D, Cenci L, Moro T, Bianco A, et al. Ketogenic diet does not affect strength performance in elite artistic gymnasts. J Int Soc Sports Nutr. 2012;9(1):1-9.

111. Rhyu H-s, Cho S-Y. The effect of weight loss by ketogenic diet on the body composition, performance-related physical fitness factors and cytokines of Taekwondo athletes. Journal of exercise rehabilitation. 2014;10(5):326.

112. Sitko S, Sastre RC, Soler FC, Laval IL. Effects of a low-carbohydrate diet on body composition and performance in road cycling: A randomized, controlled trial. 2020.

113. Tinline-Goodfellow CT, West DW, Malowany JM, Gillen JB, Moore DR. An acute reduction in habitual protein intake attenuates post exercise anabolism and may bias oxidation-derived protein requirements in resistance trained men. Frontiers in nutrition. 2020;7:55.

114. Whitfield J, Burke LM, McKay AK, Heikura IA, Hall R, Fensham N, et al. Acute ketogenic diet and ketone ester supplementation impairs race walk performance. Med Sci Sports Exerc. 2021;53(4):776.

115. de Castro MBT, Cunha DB, Araujo MC, Bezerra IN, Adegboye ARA, Kac G, et al. High protein diet promotes body weight loss among Brazilian postpartum women. Matern Child Nutr. 2019;15(3):e12746.

116. Rasmussen L, Christensen ML, Poulsen CW, Rud C, Christensen AS, Andersen JR, et al. Effect of high versus low carbohydrate intake in the morning on glycemic variability and glycemic control measured by continuous blood glucose monitoring in women with gestational diabetes mellitus—a randomized crossover study. Nutrients. 2020;12(2):475.

117. Goldstein T, Kark JD, Berry EM, Adler B, Ziv E, Raz I. The effect of a low carbohydrate energy-unrestricted diet on weight loss in obese type 2 diabetes patients–a randomized controlled trial. E Spen Eur E J Clin Nutr Metab. 2011;6(4):e178-e86.

118. Krebs J, Elley C, Parry-Strong A, Lunt H, Drury P, Bell D, et al. The Diabetes Excess Weight Loss (DEWL) Trial: a randomised controlled trial of high-protein versus high-carbohydrate diets over 2 years in type 2 diabetes. Diabetologia. 2012;55(4):905-14.

119. Azadbakht L, Izadi V, Surkan PJ, Esmaillzadeh A. Effect of a high protein weight loss diet on weight, high-sensitivity C-reactive protein, and cardiovascular risk among overweight and obese women: a parallel clinical trial. Int J Endocrinol. 2013;2013.

120. Brehm BJ, Lattin BL, Summer SS, Boback JA, Gilchrist GM, Jandacek RJ, et al. One-year comparison of a high–monounsaturated fat diet with a high-carbohydrate diet in type 2 diabetes. Diabetes Care. 2009;32(2):215-20.

121. Brunerova L, Smejkalova V, Potockova J, Andel M. A comparison of the influence of a high‐fat diet enriched in monounsaturated fatty acids and conventional diet on weight loss and metabolic parameters in obese non‐diabetic and Type 2 diabetic patients. Diabet Med. 2007;24(5):533-40.

122. Campbell WW, Tang M. Protein intake, weight loss, and bone mineral density in postmenopausal women. Journals of Gerontology Series A: Biomedical Sciences and Medical Sciences. 2010;65(10):1115-22.

123. Collins CE, Morgan PJ, Jones P, Fletcher K, Martin J, Aguiar EJ, et al. A 12-week commercial web-based weight-loss program for overweight and obese adults: randomized controlled trial comparing basic versus enhanced features. J Med Internet Res. 2012;14(2):e57.

124. Due A, Larsen TM, Mu H, Hermansen K, Stender S, Astrup A. Comparison of 3 ad libitum diets for weight-loss maintenance, risk of cardiovascular disease, and diabetes: a 6-mo randomized, controlled trial. The American journal of clinical nutrition. 2008;88(5):1232-41.

125. González-Salazar LE, Pichardo-Ontiveros E, Palacios-González B, Vigil-Martínez A, Granados-Portillo O, Guizar-Heredia R, et al. Effect of the intake of dietary protein on insulin resistance in subjects with obesity: a randomized controlled clinical trial. Eur J Nutr. 2020:1-13.

126. Group DPPR. Reduction in the incidence of type 2 diabetes with lifestyle intervention or metformin. N Engl J Med. 2002;346(6):393-403.

127. Gumbiner B, Low CC, Reaven PD. Effects of a monounsaturated fatty acid–enriched hypocaloric diet on cardiovascular risk factors in obese patients with type 2 diabetes. Diabetes Care. 1998;21(1):9-15.

128. Haghighat N, Ashtary-Larky D, Bagheri R, Mahmoodi M, Rajaei M, Alipour M, et al. The effect of 12 weeks of euenergetic high-protein diet in regulating appetite and body composition of women with normal-weight obesity: a randomised controlled trial. Br J Nutr. 2020;124(10):1044-51.

129. Johnston CA, Rost S, Miller-Kovach K, Moreno JP, Foreyt JP. A randomized controlled trial of a community-based behavioral counseling program. The American journal of medicine. 2013;126(12):1143. e19-. e24.

130. Jönsson T, Granfeldt Y, Ahrén B, Branell U-C, Pålsson G, Hansson A, et al. Beneficial effects of a Paleolithic diet on cardiovascular risk factors in type 2 diabetes: a randomized cross-over pilot study. Cardiovasc Diabetol. 2009;8(1):1-14.

131. Kasim-Karakas SE, Almario RU, Cunningham W. Effects of protein versus simple sugar intake on weight loss in polycystic ovary syndrome (according to the National Institutes of Health criteria). Fertil Steril. 2009;92(1):262-70.

132. Leidy HJ, Carnell NS, Mattes RD, Campbell WW. Higher protein intake preserves lean mass and satiety with weight loss in pre‐obese and obese women. Obesity. 2007;15(2):421-9.

133. Li X, Zhou T, Ma H, Heianza Y, Champagne CM, Williamson DA, et al. Genetic variation in lean body mass, changes of appetite and weight loss in response to diet interventions: The POUNDS Lost trial. Diabetes, Obesity and Metabolism. 2020;22(12):2305-15.

134. Lindeberg S, Jönsson T, Granfeldt Y, Borgstrand E, Soffman J, Sjöström K, et al. A Palaeolithic diet improves glucose tolerance more than a Mediterranean-like diet in individuals with ischaemic heart disease. Diabetologia. 2007;50(9):1795-807.

135. Lovejoy J, Bray G, Lefevre M, Smith S, Most M, Denkins Y, et al. Consumption of a controlled low-fat diet containing olestra for 9 months improves health risk factors in conjunction with weight loss in obese men: the Ole'Study. Int J Obes. 2003;27(10):1242-9.

136. Luscombe N, Noakes M, Clifton P. Diets high and low in glycemic index versus high monounsaturated fat diets: effects on glucose and lipid metabolism in NIDDM. Eur J Clin Nutr. 1999;53(6):473-8.

137. Meroni A, Muirhead RP, Atkinson FS, Fogelholm M, Raben A, Brand-Miller JC. Is a higher protein-lower glycemic index diet more nutritious than a conventional diet? a PREVIEW sub-study. Frontiers in nutrition. 2020;7:299.

138. Nahas PC, Rossato LT, Martins FM, Souza AP, de Branco F, Carneiro MA, et al. Moderate increase in protein intake promotes a small additional improvement in functional capacity, but not in muscle strength and lean mass quality, in postmenopausal women following resistance exercise: a randomized clinical trial. Nutrients. 2019;11(6):1323.

139. Noakes M, Keogh JB, Foster PR, Clifton PM. Effect of an energy-restricted, high-protein, low-fat diet relative to a conventional high-carbohydrate, low-fat diet on weight loss, body composition, nutritional status, and markers of cardiovascular health in obese women. The American journal of clinical nutrition. 2005;81(6):1298-306.

140. Noer ER, Dewi L, Huriyati E, Djamiatun K, Susanto H, Tjahjono K, et al. The Impact of Weight Loss Program in Metabolic Profile and Body Fat Improvement among Indonesian Young Obese: A Randomized Clinical Trial. J Nutr Sci Vitaminol (Tokyo). 2020;66(Supplement):S308-S13.

141. Pasman W, Westerterp-Plantenga M, Saris W. The effectiveness of long-term supplementation of carbohydrate, chromium, fibre and caffeine on weight maintenance. Int J Obes. 1997;21(12):1143-51.

142. Pelkman CL, Fishell VK, Maddox DH, Pearson TA, Mauger DT, Kris-Etherton PM. Effects of moderate-fat (from monounsaturated fat) and low-fat weight-loss diets on the serum lipid profile in overweight and obese men and women. The American journal of clinical nutrition. 2004;79(2):204-12.

143. Raben A, Vestentoft PS, Brand‐Miller J, Jalo E, Drummen M, Simpson L, et al. The PREVIEW intervention study: Results from a 3‐year randomized 2 x 2 factorial multinational trial investigating the role of protein, glycaemic index and physical activity for prevention of type 2 diabetes. Diabetes, Obesity and Metabolism. 2021;23(2):324-37.

144. Rock CL, Flatt SW, Pakiz B, Taylor KS, Leone AF, Brelje K, et al. Weight loss, glycemic control, and cardiovascular disease risk factors in response to differential diet composition in a weight loss program in type 2 diabetes: a randomized controlled trial. Diabetes Care. 2014;37(6):1573-80.

145. Rodríguez-Hernández H, Cervantes-Huerta M, Rodríguez-Moran M, Guerrero-Romero F. Decrease of aminotransferase levels in obese women is related to body weight reduction, irrespective of type of diet. Ann Hepatol. 2016;10(4):486-92.

146. Rothberg AE, Herman WH, Wu C, IglayReger HB, Horowitz JF, Burant CF, et al. Weight Loss Improves β-Cell Function in People With Severe Obesity and Impaired Fasting Glucose: A Window of Opportunity. The Journal of Clinical Endocrinology & Metabolism. 2020;105(4):e1621-e30.

147. Saglam D, Saka M, Sayaca N. The effect of consumption of low glycemic index, high fat content bread on anthropometric measurement and cardiometabolic risk factors in women with type 2 diabetes mellitus. Int J Diabetes Dev Ctries. 2019;39(1):166-72.

148. Saris W, Astrup A, Prentice A, Zunft H, Formiguera X, Verboeket-Van De Venne W, et al. Randomized controlled trial of changes in dietary carbohydrate/fat ratio and simple vs complex carbohydrates on body weight and blood lipids: the CARMEN study. Int J Obes. 2000;24(10):1310-8.

149. Shirai K, Saiki A, Oikawa S, Teramoto T, Yamada N, Ishibashi S, et al. The effects of partial use of formula diet on weight reduction and metabolic variables in obese type 2 diabetic patients—multicenter trial. Obes Res Clin Pract. 2013;7(1):e43-e54.

150. Sowah SA, Hirche F, Milanese A, Johnson TS, Grafetstätter M, Schübel R, et al. Changes in plasma short-chain fatty acid levels after dietary weight loss among overweight and obese adults over 50 weeks. Nutrients. 2020;12(2):452.

151. Tang KY, Huang S-Y, Cheng T-M, Bai C-H, Chang J-S. Haptoglobin phenotype influences the effectiveness of diet-induced weight loss in middle-age abdominally obese women with metabolic abnormalities. Clin Nutr. 2020;39(1):225-33.

152. Tebbe A, Weiss W. Concurrent and carryover effects of feeding blends of protein and amino acids in high-protein diets with different concentrations of forage fiber to fresh cows. 1. Production and blood metabolites. J Dairy Sci. 2021;104(5):5583-600.

153. Tok Ö, Kişioğlu SV, Ersöz HÖ, Kahveci B, Göktaş Z. Effects of increased physical activity and/or weight loss diet on serum myokine and adipokine levels in overweight adults with impaired glucose metabolism. J Diabetes Complications. 2021;35(5):107892.

154. Vandenberghe C, Castellano C-A, Maltais M, Fortier M, St-Pierre V, Dionne IJ, et al. A short-term intervention combining aerobic exercise with medium-chain triglycerides (MCT) is more ketogenic than either MCT or aerobic exercise alone: a comparison of normoglycemic and prediabetic older women. Applied Physiology, Nutrition, and Metabolism. 2019;44(1):66-73.

155. Yılmaz SK, Eskici G, Mertoǧlu C, Ayaz A. Effect of different protein diets on weight loss, inflammatory markers, and cardiometabolic risk factors in obese women. Journal of Research in Medical Sciences: The Official Journal of Isfahan University of Medical Sciences. 2021;26.

156. Dellis D, Tsilingiris D, Eleftheriadou I, Tentolouris A, Sfikakis Jr PP, Dellis G, et al. Carbohydrate restriction in the morning increases weight loss effect of a hypocaloric Mediterranean type diet: a randomized, parallel group dietary intervention in overweight and obese subjects. Nutrition. 2020;71:110578.

157. Kempf K, Röhling M, Banzer W, Braumann KM, Halle M, McCarthy D, et al. High-protein, low-glycaemic meal replacement decreases fasting insulin and inflammation markers—a 12-month subanalysis of the ACOORH trial. Nutrients. 2021;13(5):1433.

158. Lu LW, Silvestre MP, Sequeira IR, Plank LD, Foster M, Middleditch N, et al. A higher-protein nut-based snack product suppresses glycaemia and decreases glycaemic response to co-ingested carbohydrate in an overweight prediabetic Asian Chinese cohort: the Tū Ora postprandial RCT. Journal of nutritional science. 2021;10.

159. Mitri J, Tomah S, Furtado J, Tasabehji MW, Hamdy O. Plasma Free Fatty Acids and Metabolic Effect in Type 2 Diabetes, an Ancillary Study from a Randomized Clinical Trial. Nutrients. 2021;13(4):1145.

160. Silva CM, Cunha NBD, Mota MC, Marot LP, Teixeira KRC, Cunha TM, et al. Effect of consuming a late-night high-protein/moderate-carbohydrate vs. low-protein/high-carbohydrate meal by night workers on their food perceptions later during the day: a randomized crossover study. Chronobiol Int. 2020;37(9-10):1392-9.

161. Treyzon L, Chen S, Hong K, Yan E, Carpenter CL, Thames G, et al. A controlled trial of protein enrichment of meal replacements for weight reduction with retention of lean body mass. Nutr J. 2008;7(1):1-6.

162. Abbie E, Francois ME, Chang CR, Barry JC, Little JP. A low-carbohydrate protein-rich bedtime snack to control fasting and nocturnal glucose in type 2 diabetes: A randomized trial. Clin Nutr. 2020;39(12):3601-6.

163. Al‐Sari N, Schmidt S, Suvitaival T, Kim M, Trošt K, Ranjan AG, et al. Changes in the lipidome in type 1 diabetes following low carbohydrate diet: Post‐hoc analysis of a randomized crossover trial. Endocrinology, diabetes & metabolism. 2021;4(2):e00213.

164. Ashton EL, Pomeroy S, Foster JE, Kaye RS, Nestel PJ, Ball M. Diet High in Monounsaturated Fat does not have a Different Effect on Arterial Elasticity than a Low Fat, High Carbohydrate Diet. J Am Diet Assoc. 2000;100(5):537-42.

165. Augustus E, Granderson I, Rocke KD. The Impact of a Ketogenic Dietary Intervention on the Quality of Life of Stage II and III Cancer Patients: A Randomized Controlled Trial in the Caribbean. Nutr Cancer. 2020:1-11.

166. Benlloch M, López-Rodríguez MM, Cuerda-Ballester M, Drehmer E, Carrera S, Ceron JJ, et al. Satiating effect of a ketogenic diet and its impact on muscle improvement and oxidation state in multiple sclerosis patients. Nutrients. 2019;11(5):1156.

167. BORKMAN M, CAMPBELL LV, CHISHOLM DJ, STORLIEN LH. Comparison of the effects on insulin sensitivity of high carbohydrate and high fat diets in normal subjects. The Journal of Clinical Endocrinology & Metabolism. 1991;72(2):432-7.

168. Brandt J, Buchholz A, Henry-Barron B, Vizthum D, Avramopoulos D, Cervenka MC. Preliminary report on the feasibility and efficacy of the modified Atkins diet for treatment of mild cognitive impairment and early Alzheimer’s disease. J Alzheimers Dis. 2019;68(3):969-81.

169. Breukelman GJ, Basson AK, Djarova TG, Du Preez CJ, Shaw I, Malan H, et al. Concurrent low-carbohydrate, high-fat diet with/without physical activity does not improve glycaemic control in type 2 diabetics. South Afr J Clin Nutr. 2021;34(1):18-21.

170. Budipramana VS, Arifin F. Very Low Carbohydrate Diet Effect on Modified Glasgow Prognostic Score: A Randomized Controlled Trial on Stage-IV Colorectal Adenocarcinoma Patients. Surgery, Gastroenterology and Oncology. 2020;25(5):280-5.

171. Burén J, Ericsson M, Damasceno NRT, Sjödin A. A ketogenic low-carbohydrate high-fat diet increases LDL cholesterol in healthy, young, normal-weight women: A randomized controlled feeding trial. Nutrients. 2021;13(3):814.

172. Burke LM, Sharma AP, Heikura IA, Forbes SF, Holloway M, McKay AK, et al. Crisis of confidence averted: Impairment of exercise economy and performance in elite race walkers by ketogenic low carbohydrate, high fat (LCHF) diet is reproducible. PLoS One. 2020;15(6):e0234027.

173. Chan Y-M, Aufreiter S, O’Keefe SJ, O’Connor DL. Switching to a fibre-rich and low-fat diet increases colonic folate contents among African Americans. Applied Physiology, Nutrition, and Metabolism. 2019;44(2):127-32.

174. Chang CR, Francois ME, Little JP. Restricting carbohydrates at breakfast is sufficient to reduce 24-hour exposure to postprandial hyperglycemia and improve glycemic variability. The American journal of clinical nutrition. 2019;109(5):1302-9.

175. Charidemou E, Ashmore T, Li X, McNally BD, West JA, Liggi S, et al. A randomized 3-way crossover study indicates that high-protein feeding induces de novo lipogenesis in healthy humans. JCI insight. 2019;4(12).

176. Chen C-Y, Huang W-S, Chen H-C, Chang C-H, Lee L-T, Chen H-S, et al. Effect of a 90 g/day low-carbohydrate diet on glycaemic control, small, dense low-density lipoprotein and carotid intima-media thickness in type 2 diabetic patients: An 18-month randomised controlled trial. PLoS One. 2020;15(10):e0240158.

177. Chen X, Su H, Kunii D, Kudou K, Zhang Y, Zhao Y, et al. The Effects of Mobile-App-Based Low-Carbohydrate Dietary Guidance on Postprandial Hyperglycemia in Adults with Prediabetes. Diabetes Ther. 2020;11(10):2341-55.

178. Cipryan L, Dostal T, Plews DJ, Hofmann P, Laursen PB. Adiponectin/leptin ratio increases after a 12-week very low-carbohydrate, high-fat diet, and exercise training in healthy individuals: A non-randomized, parallel design study. Nutr Res. 2021;87:22-30.

179. Cipryan L, Maffetone PB, Plews DJ, Laursen PB. Effects of a four-week very low-carbohydrate high-fat diet on biomarkers of inflammation: Non-randomised parallel-group study. Nutr Health. 2020;26(1):35-42.

180. Clifford T, Hayes EJ, Scragg JH, Taylor G, Smith K, Davies KAB, et al. The effects of a high-protein diet on markers of muscle damage following exercise in active older adults: a randomized, controlled trial. Int J Sport Nutr Exerc Metab. 2020;30(5):323-9.

181. Cohen CW, Fontaine KR, Arend RC, Gower BA. A ketogenic diet is acceptable in women with ovarian and endometrial cancer and has no adverse effects on blood lipids: a randomized, controlled trial. Nutr Cancer. 2020;72(4):584-94.

182. Costanzo A, Liu D, Nowson C, Duesing K, Archer N, Bowe S, et al. A low-fat diet up-regulates expression of fatty acid taste receptor gene FFAR4 in fungiform papillae in humans: A co-twin randomised controlled trial. Br J Nutr. 2019;122(11):1212-20.

183. Dahl WJ, Hung W-L, Ford AL, Suh JH, Auger J, Nagulesapillai V, et al. In older women, a high-protein diet including animal-sourced foods did not impact serum levels and urinary excretion of trimethylamine-N-oxide. Nutr Res. 2020;78:72-81.

184. de Souza Neves G, dos Santos Lunardi M, Gabiatti MP, Venske DKR, Ribeiro LC, Lin K, et al. Cardiometabolic risk and effectiveness of the modified Atkins ketogenic diet for adult patients with pharmacoresistant epilepsies in a middle-income country. Epilepsy Res. 2020;160:106280.

185. Di Lorenzo C, Coppola G, Bracaglia M, Di Lenola D, Sirianni G, Rossi P, et al. A ketogenic diet normalizes interictal cortical but not subcortical responsivity in migraineurs. BMC Neurol. 2019;19(1):1-9.

186. Dimosthenopoulos C, Liatis S, Kourpas E, Athanasopoulou E, Driva S, Makrilakis K, et al. The beneficial short‐term effects of a high‐protein/low‐carbohydrate diet on glycaemic control assessed by continuous glucose monitoring in patients with type 1 diabetes. Diabetes, Obesity and Metabolism. 2021.

187. Dirks ML, Wall BT, Otten B, Cruz AM, Dunlop MV, Barker AR, et al. High-fat overfeeding does not exacerbate rapid changes in forearm glucose and fatty acid balance during immobilization. The Journal of Clinical Endocrinology & Metabolism. 2020;105(1):276-89.

188. Dorling JL, Das SK, Racette SB, Apolzan JW, Zhang D, Pieper CF, et al. Changes in body weight, adherence, and appetite during 2 years of calorie restriction: the CALERIE 2 randomized clinical trial. Eur J Clin Nutr. 2020;74(8):1210-20.

189. Dorling JL, Ravussin E, Redman LM, Bhapkar M, Huffman KM, Racette SB, et al. Effect of 2 years of calorie restriction on liver biomarkers: results from the CALERIE phase 2 randomized controlled trial. Eur J Nutr. 2021;60(3):1633-43.

190. Dostal T, Plews DJ, Hofmann P, Laursen PB, Cipryan L. Effects of a 12-week very-low carbohydrate high-fat diet on maximal aerobic capacity, high-intensity intermittent exercise, and cardiac autonomic regulation: non-randomized parallel-group study. Front Physiol. 2019;10:912.

191. Durainayagam B, Mitchell CJ, Milan AM, Zeng N, Sharma P, Mitchell SM, et al. Impact of a high protein intake on the plasma metabolome in elderly males: 10 week randomized dietary intervention. Frontiers in nutrition. 2019;6:180.

192. Estruch R, Martínez-González MA, Corella D, Salas-Salvadó J, Fitó M, Chiva-Blanch G, et al. Effect of a high-fat Mediterranean diet on bodyweight and waist circumference: a prespecified secondary outcomes analysis of the PREDIMED randomised controlled trial. The Lancet Diabetes & Endocrinology. 2019;7(5):e6-e17.

193. Evans M, Smart C, Paramalingam N, Smith G, Jones T, King B, et al. Dietary protein affects both the dose and pattern of insulin delivery required to achieve postprandial euglycaemia in type 1 diabetes: a randomized trial. Diabet Med. 2019;36(4):499-504.

194. Falco-Walter JJ, Roehl K, Ouyang B, Balabanov A. Do certain subpopulations of adults with drug-resistant epilepsy respond better to modified ketogenic diet treatments? Evaluation based on prior resective surgery, type of epilepsy, imaging abnormalities, and vagal nerve stimulation. Epilepsy Behav. 2019;93:119-24.

195. Ferrera HK, Jones TE, Schudrowitz NJ, Collins JE, Lichstein PM, Shaner JL, et al. Perioperative dietary restriction of carbohydrates in the management of blood glucose levels in patients undergoing total knee replacement. The Journal of arthroplasty. 2019;34(6):1105-9.

196. Foppiani A, De Amicis R, Lessa C, Leone A, Ravella S, Ciusani E, et al. Isocaloric ketogenic diet in adults with high-grade gliomas: a prospective metabolic study. Nutr Cancer. 2021;73(6):1004-14.

197. Fortier M, Castellano C-A, Croteau E, Langlois F, Bocti C, St-Pierre V, et al. A ketogenic drink improves brain energy and some measures of cognition in mild cognitive impairment. Alzheimer's & Dementia. 2019;15(5):625-34.

198. Francis BA, Fillenworth J, Gorelick P, Karanec K, Tanner A. The feasibility, safety and effectiveness of a ketogenic diet for refractory status epilepticus in adults in the intensive care unit. Neurocrit Care. 2019;30(3):652-7.

199. Gibson MJ, Dawson JA, Wijayatunga NN, Ironuma B, Chatindiara I, Ovalle F, et al. A randomized cross-over trial to determine the effect of a protein vs. carbohydrate preload on energy balance in ad libitum settings. Nutr J. 2019;18(1):1-13.

200. Gillingham MB, Elizondo G, Behrend A, Matern D, Schoeller DA, Harding CO, et al. Higher dietary protein intake preserves lean body mass, lowers liver lipid deposition, and maintains metabolic control in participants with long‐chain fatty acid oxidation disorders. J Inherit Metab Dis. 2019;42(5):857-69.

201. Guo J, Robinson JL, Gardner CD, Hall KD. Objective versus Self‐Reported Energy Intake Changes During Low‐Carbohydrate and Low‐Fat Diets. Obesity. 2019;27(3):420-6.

202. Gyorkos A, Baker MH, Miutz LN, Lown DA, Jones MA, Houghton-Rahrig LD. Carbohydrate-restricted diet and exercise increase brain-derived neurotrophic factor and cognitive function: a randomized crossover trial. Cureus. 2019;11(9).

203. Hadizadeh M, Gan WY, Mohafez H, Sugajima Y. Impact of Ketogenic Diet on Body Composition during Resistance Training among Untrained Individuals. The Open Sports Sciences Journal. 2020;13(1).

204. Harvey CJdC, Schofield GM, Zinn C, Thornley SJ, Crofts C, Merien FL. Low-carbohydrate diets differing in carbohydrate restriction improve cardiometabolic and anthropometric markers in healthy adults: A randomised clinical trial. PeerJ. 2019;7:e6273.

205. Heikura IA, Burke LM, Hawley JA, Ross ML, Garvican-Lewis L, Sharma AP, et al. A short-term ketogenic diet impairs markers of bone health in response to exercise. Front Endocrinol (Lausanne). 2020;10:880.

206. Hevia-Larraín V, Gualano B, Longobardi I, Gil S, Fernandes AL, Costa LA, et al. High-protein plant-based diet versus a protein-matched omnivorous diet to support resistance training adaptations: a comparison between habitual vegans and omnivores. Sports Med. 2021;51(6):1317-30.

207. Hockaday T, Hockaday J, Mann J, Turner R. Prospective comparison of modified-fat–high-carbohydrate with standard low-carbohydrate dietary advice in the treatment of diabetes: one year follow-up study. Br J Nutr. 1978;39(2):357-62.

208. Højfeldt G, Bülow J, Agergaard J, Asmar A, Schjerling P, Simonsen L, et al. Impact of habituated dietary protein intake on fasting and postprandial whole-body protein turnover and splanchnic amino acid metabolism in elderly men: a randomized, controlled, crossover trial. The American journal of clinical nutrition. 2020;112(6):1468-84.

209. Højfeldt G, Bülow J, Agergaard J, Simonsen LR, Bülow J, Schjerling P, et al. Postprandial muscle protein synthesis rate is unaffected by 20-day habituation to a high protein intake: a randomized controlled, crossover trial. Eur J Nutr. 2021:1-13.

210. Holloway CJ, Cochlin LE, Emmanuel Y, Murray A, Codreanu I, Edwards LM, et al. A high-fat diet impairs cardiac high-energy phosphate metabolism and cognitive function in healthy human subjects. The American journal of clinical nutrition. 2011;93(4):748-55.

211. Hu J-R, Wu Y, Sacks FM, Appel LJ, Miller III ER, Young JH, et al. Effects of carbohydrate quality and amount on plasma lactate: results from the OmniCarb trial. BMJ Open Diabetes Research and Care. 2020;8(1):e001457.

212. Hyde PN, Sapper TN, LaFountain RA, Kackley ML, Buga A, Fell B, et al. Effects of Palm Stearin versus Butter in the Context of Low-Carbohydrate/High-Fat and High-Carbohydrate/Low-Fat Diets on Circulating Lipids in a Controlled Feeding Study in Healthy Humans. Nutrients. 2021;13(6):1944.

213. Iyikesici MS. Long-term survival outcomes of metabolically supported chemotherapy with gemcitabine-based or FOLFIRINOX regimen combined with ketogenic diet, hyperthermia, and hyperbaric oxygen therapy in metastatic pancreatic cancer. Complementary medicine research. 2020;27(1):31-9.

214. Jacobs B, De Angelis-Schierbaum G, Egert S, Assmann G, Kratz M. Individual serum triglyceride responses to high-fat and low-fat diets differ in men with modest and severe hypertriglyceridemia. The Journal of nutrition. 2004;134(6):1400-5.

215. Johari MI, Yusoff K, Haron J, Nadarajan C, Ibrahim KN, Wong MS, et al. A randomised controlled trial on the effectiveness and adherence of modified alternate-day calorie restriction in improving activity of non-alcoholic fatty liver disease. Sci Rep. 2019;9(1):1-9.

216. Johnston CS, Tjonn SL, Swan PD. High-protein, low-fat diets are effective for weight loss and favorably alter biomarkers in healthy adults. The Journal of nutrition. 2004;134(3):586-91.

217. Kämmerer U, Klement RJ, Joos FT, Sütterlin M, Reuss-Borst M. Low Carb and Ketogenic Diets Increase Quality of Life, Physical Performance, Body Composition, and Metabolic Health of Women with Breast Cancer. Nutrients. 2021;13(3):1029.

218. Kang CM, Yun B, Kim M, Song M, Kim Y-h, Lee SH, et al. Postoperative serum metabolites of patients on a low carbohydrate ketogenic diet after pancreatectomy for pancreatobiliary cancer: A nontargeted metabolomics pilot study. Sci Rep. 2019;9(1):1-11.

219. Kasim SE, Martino S, Kim P-N, Khilnani S, Boomer A, Depper J, et al. Dietary and anthropometric determinants of plasma lipoproteins during a long-term low-fat diet in healthy women. The American journal of clinical nutrition. 1993;57(2):146-53.

220. Kennedy R, Chokkalingam K, Farshchi H. Nutrition in patients with Type 2 diabetes: are low‐carbohydrate diets effective, safe or desirable? Diabet Med. 2005;22(7):821-32.

221. Khodabakhshi A, Akbari ME, Mirzaei HR, Mehrad-Majd H, Kalamian M, Davoodi SH. Feasibility, safety, and beneficial effects of MCT-based ketogenic diet for breast cancer treatment: a randomized controlled trial study. Nutr Cancer. 2020;72(4):627-34.

222. Khodabakhshi A, Akbari ME, Mirzaei HR, Seyfried TN, Kalamian M, Davoodi SH. Effects of Ketogenic metabolic therapy on patients with breast cancer: A randomized controlled clinical trial. Clin Nutr. 2021;40(3):751-8.

223. Khodabakhshi A, Seyfried TN, Kalamian M, Beheshti M, Davoodi SH. Does a ketogenic diet have beneficial effects on quality of life, physical activity or biomarkers in patients with breast cancer: a randomized controlled clinical trial. Nutr J. 2020;19(1):1-10.

224. Khong TK, Kimpton J. Moderate calorie restriction improves cardiometabolic risk factors in healthy individuals. Drug Ther Bull. 2020;58(9):135-6.

225. Kip P, Trocha KM, Tao M, O’leary JJ, Ruske J, Giulietti JM, et al. Insights From a Short-Term Protein–Calorie Restriction Exploratory Trial in Elective Carotid Endarterectomy Patients. Vasc Endovascular Surg. 2019;53(6):470-6.

226. Klein P, Tyrlikova I, Zuccoli G, Tyrlik A, Maroon JC. Treatment of glioblastoma multiforme with “classic” 4: 1 ketogenic diet total meal replacement. Cancer & Metabolism. 2020;8(1):1-11.

227. Klement RJ, Champ CE, Kämmerer U, Koebrunner PS, Krage K, Schäfer G, et al. Impact of a ketogenic diet intervention during radiotherapy on body composition: III—final results of the KETOCOMP study for breast cancer patients. Breast Cancer Res. 2020;22(1):1-14.

228. Kraus WE, Bhapkar M, Huffman KM, Pieper CF, Das SK, Redman LM, et al. 2 years of calorie restriction and cardiometabolic risk (CALERIE): exploratory outcomes of a multicentre, phase 2, randomised controlled trial. The lancet Diabetes & endocrinology. 2019;7(9):673-83.

229. Krebs JD, Strong AP, Cresswell P, Reynolds AN, Hanna A, Haeusler S. A randomised trial of the feasibility of a low carbohydrate diet vs standard carbohydrate counting in adults with type 1 diabetes taking body weight into account. Asia Pac J Clin Nutr. 2016;25(1):78-84.

230. Kumar N, Warren GL, Snow TK, Millard-Stafford M. Caffeine ingestion with or without low-dose carbohydrate improves exercise tolerance in sedentary adults. Frontiers in nutrition. 2019;6:9.

231. Kverneland M, Taubøll E, Molteberg E, Veierød MB, Selmer KK, Nakken KO, et al. Pharmacokinetic interaction between modified Atkins diet and antiepileptic drugs in adults with drug‐resistant epilepsy. Epilepsia. 2019;60(11):2235-44.

232. Kysel P, Haluzíková D, Doležalová RP, Laňková I, Lacinová Z, Kasperová BJ, et al. The influence of cyclical ketogenic reduction diet vs. Nutritionally balanced reduction diet on body composition, strength, and endurance performance in healthy young males: A randomized controlled trial. Nutrients. 2020;12(9):2832.

233. LaFountain RA, Miller VJ, Barnhart EC, Hyde PN, Crabtree CD, McSwiney FT, et al. Extended ketogenic diet and physical training intervention in military personnel. Mil Med. 2019;184(9-10):e538-e47.

234. Larsen MS, Holm L, Svart MV, Hjelholt AJ, Bengtsen MB, Dollerup OL, et al. Effects of protein intake prior to carbohydrate-restricted endurance exercise: a randomized crossover trial. J Int Soc Sports Nutr. 2020;17(1):1-13.

235. Lee DC, Vali K, Baldwin SR, Divino JN, Feliciano JL, Fequiere JR, et al. Dietary supplementation with the ketogenic diet metabolite beta-hydroxybutyrate ameliorates post-TBI aggression in young-adult male Drosophila. Front Neurosci. 2019;13:1140.

236. Lee JE, Titcomb TJ, Bisht B, Rubenstein LM, Louison R, Wahls TL. A modified MCT-based ketogenic diet increases plasma β-hydroxybutyrate but has less effect on fatigue and quality of life in people with multiple sclerosis compared to a modified paleolithic diet: a waitlist-controlled, randomized pilot study. J Am Coll Nutr. 2021;40(1):13-25.

237. Liu K, Wang B, Zhou R, Lang H-D, Ran L, Wang J, et al. Effect of combined use of a low-carbohydrate, high-protein diet with omega-3 polyunsaturated fatty acid supplementation on glycemic control in newly diagnosed type 2 diabetes: a randomized, double-blind, parallel-controlled trial. The American journal of clinical nutrition. 2018;108(2):256-65.

238. Martin-McGill KJ, Marson AG, Smith CT, Young B, Mills SJ, Cherry MG, et al. Ketogenic diets as an adjuvant therapy for glioblastoma (KEATING): a randomized, mixed methods, feasibility study. J Neurooncol. 2020:1-15.

239. McNairn M, Brito A, Dillard K, Heath H, Pantaleon M, Fanter R, et al. Postprandial Dried Blood Spot–Based Nutritional Metabolomic Analysis Discriminates a High-Fat, High-Protein Meat-Based Diet from a High Carbohydrate Vegan Diet: A Randomized Controlled Crossover Trial. J Acad Nutr Diet. 2021;121(5):931-41. e2.

240. Michalczyk MM, Maszczyk A, Stastny P. The effects of low-energy moderate-carbohydrate (MCD) and mixed (MixD) diets on serum lipid profiles and body composition in middle-aged men: a randomized controlled parallel-group clinical trial. Int J Environ Res Public Health. 2020;17(4):1332.

241. Miller VJ, LaFountain RA, Barnhart E, Sapper TS, Short J, Arnold WD, et al. A ketogenic diet combined with exercise alters mitochondrial function in human skeletal muscle while improving metabolic health. American Journal of Physiology-Endocrinology and Metabolism. 2020;319(6):E995-E1007.

242. Mitchell S, McKenzie E, Mitchell C, Milan A, Zeng N, D'Souza R, et al. A period of 10 weeks of increased protein consumption does not alter faecal microbiota or volatile metabolites in healthy older men: A randomised controlled trial. Journal of nutritional science. 2020;9.

243. Monteyne AJ, Dunlop MV, Machin DJ, Coelho MO, Pavis GF, Porter C, et al. A mycoprotein-based high-protein vegan diet supports equivalent daily myofibrillar protein synthesis rates compared with an isonitrogenous omnivorous diet in older adults: a randomised controlled trial. Br J Nutr. 2021;126(5):674-84.

244. Morrison SA, Fazeli PL, Gower B, Willig AL, Younger J, Sneed NM, et al. Cognitive effects of a ketogenic diet on neurocognitive impairment in adults aging with HIV: a pilot study. The Journal of the Association of Nurses in AIDS Care: JANAC. 2020;31(3):312.

245. Murphy C, Bilek LD, Koehler K. Low Energy Availability with and without a High-Protein Diet Suppresses Bone Formation and Increases Bone Resorption in Men: A Randomized Controlled Pilot Study. Nutrients. 2021;13(3):802.

246. Nabuco HC, Tomeleri CM, Fernandes RR, Junior PS, Cavalcante EF, Venturini D, et al. Effects of protein intake beyond habitual intakes associated with resistance training on metabolic syndrome-related parameters, isokinetic strength, and body composition in older women. J Aging Phys Act. 2019;27(4):545-52.

247. Nagpal R, Neth BJ, Wang S, Craft S, Yadav H. Modified Mediterranean-ketogenic diet modulates gut microbiome and short-chain fatty acids in association with Alzheimer's disease markers in subjects with mild cognitive impairment. EBioMedicine. 2019;47:529-42.

248. Nakagata T, Tamura Y, Kaga H, Sato M, Yamasaki N, Someya Y, et al. Ingestion of an exogenous ketone monoester improves the glycemic response during oral glucose tolerance test in individuals with impaired glucose tolerance: A cross‐over randomized trial. Journal of Diabetes Investigation. 2021;12(5):756-62.

249. Neth BJ, Mintz A, Whitlow C, Jung Y, Sai KS, Register TC, et al. Modified ketogenic diet is associated with improved cerebrospinal fluid biomarker profile, cerebral perfusion, and cerebral ketone body uptake in older adults at risk for Alzheimer’s disease: A pilot study. Neurobiol Aging. 2020;86:54-63.

250. Nuttall FQ, Gannon MC. Effect of a LoBAG 30 diet on protein metabolism in men with type 2 diabetes. A Randomized Controlled Trial. Nutr Metab (Lond). 2012;9(1):1-12.

251. Oliveira CL, Boulé NG, Sharma AM, Elliott SA, Siervo M, Ghosh S, et al. A high-protein total diet replacement increases energy expenditure and leads to negative fat balance in healthy, normal-weight adults. The American journal of clinical nutrition. 2021;113(2):476-87.

252. Ornish D, Brown SE, Billings J, Scherwitz L, Armstrong WT, Ports TA, et al. Can lifestyle changes reverse coronary heart disease?: The Lifestyle Heart Trial. The Lancet. 1990;336(8708):129-33.

253. Oshakbayev K, Bimbetov B, Manekenova K, Bedelbayeva G, Mustafin K, Dukenbayeva B. Severe nonalcoholic steatohepatitis and type 2 diabetes: liver histology after weight loss therapy in a randomized clinical trial. Curr Med Res Opin. 2019;35(1):157-65.

254. Ota M, Matsuo J, Ishida I, Takano H, Yokoi Y, Hori H, et al. Effects of a medium-chain triglyceride-based ketogenic formula on cognitive function in patients with mild-to-moderate Alzheimer’s disease. Neurosci Lett. 2019;690:232-6.

255. Park J, Miller M, Rhyne J, Wang Z, Hazen S. Differential effect of short-term popular diets on TMAO and other cardio-metabolic risk markers. Nutrition, Metabolism and Cardiovascular Diseases. 2019;29(5):513-7.

256. Phillips MC, Deprez LM, Mortimer GM, Murtagh DK, McCoy S, Mylchreest R, et al. Randomized crossover trial of a modified ketogenic diet in Alzheimer’s disease. Alzheimers Res Ther. 2021;13(1):1-12.

257. Ramos C, Cheng AJ, Kamandulis S, Subocius A, Brazaitis M, Venckunas T, et al. Carbohydrate restriction following strenuous glycogen-depleting exercise does not potentiate the acute molecular response associated with mitochondrial biogenesis in human skeletal muscle. Eur J Appl Physiol. 2021;121(4):1219-32.

258. Ramzan F, Mitchell CJ, Milan AM, Schierding W, Zeng N, Sharma P, et al. Comprehensive profiling of the circulatory miRNAome response to a high protein diet in elderly men: a potential role in inflammatory response modulation. Mol Nutr Food Res. 2019;63(8):1800811.

259. Ranjan A, Schmidt S, Damm‐Frydenberg C, Holst JJ, Madsbad S, Nørgaard K. Short‐term effects of a low carbohydrate diet on glycaemic variables and cardiovascular risk markers in patients with type 1 diabetes: A randomized open‐label crossover trial. Diabetes, Obesity and Metabolism. 2017;19(10):1479-84.

260. Ravnskov U. Is High Cholesterol Deleterious? An Alternative Point of View. Comment on Burén et al. A Ketogenic Low-Carbohydrate High-Fat Diet Increases LDL Cholesterol in Healthy, Young, Normal-Weight Women: A Randomized Controlled Feeding Trial. Nutrients 2021, 13, 814. Nutrients. 2021;13(6):2119.

261. Rippee M, Chen J, Taylor M. The Ketogenic Diet in the Treatment of Post-concussion Syndrome—A Feasibility Study. Frontiers in nutrition. 2020;7:160.

262. Roberts J, Zinchenko A, Mahbubani KT, Johnstone J, Smith L, Merzbach V, et al. Satiating Effect of High Protein Diets on Resistance-Trained Individuals in Energy Deficit. Nutrients. 2019;11(1):56.

263. Rodriguez‐Villar C, Perez‐Heras A, Mercade I, Casals E, Ros E. Comparison of a high‐carbohydrate and a high‐monounsaturated fat, olive oil‐rich diet on the susceptibility of LDL to oxidative modification in subjects with Type 2 diabetes mellitus. Diabet Med. 2004;21(2):142-9.

264. Saidi K, Nilholm C, Roth B, Ohlsson B. A carbohydrate-restricted diet for patients with irritable bowel syndrome lowers serum C-peptide, insulin, and leptin without any correlation with symptom reduction. Nutr Res. 2021;86:23-36.

265. Silva TR, Lago SC, Yavorivski A, Ferreira LL, Fighera TM, Spritzer PM. Effects of high protein, low-glycemic index diet on lean body mass, strength, and physical performance in late postmenopausal women: a randomized controlled trial. Menopause. 2021;28(3):307-17.

266. Sjödin A, Hellström F, Sehlstedt E, Svensson M, Burén J. Effects of a ketogenic diet on muscle fatigue in healthy, young, normal-weight women: a randomized controlled feeding trial. Nutrients. 2020;12(4):955.

267. Skytte MJ, Samkani A, Astrup A, Frystyk J, Rehfeld JF, Holst JJ, et al. Effects of carbohydrate restriction on postprandial glucose metabolism, β-cell function, gut hormone secretion, and satiety in patients with Type 2 diabetes. American Journal of Physiology-Endocrinology and Metabolism. 2021;320(1):E7-E18.

268. Stoernell CK, Tangney CC, Rockway SW. Short-term changes in lipoprotein subclasses and C-reactive protein levels of hypertriglyceridemic adults on low-carbohydrate and low-fat diets. Nutr Res. 2008;28(7):443-9.

269. Straznicky NE, O’Callaghan CJ, Barrington VE, Louis WJ. Hypotensive effect of low-fat, high-carbohydrate diet can be independent of changes in plasma insulin concentrations. Hypertension. 1999;34(4):580-5.

270. Strychar I, Cohn JS, Renier G, Rivard M, Aris-Jilwan N, Beauregard H, et al. Effects of a diet higher in carbohydrate/lower in fat versus lower in carbohydrate/higher in monounsaturated fat on postmeal triglyceride concentrations and other cardiovascular risk factors in type 1 diabetes. Diabetes Care. 2009;32(9):1597-9.

271. Taylor MK, Swerdlow RH, Burns JM, Sullivan DK. An experimental ketogenic diet for Alzheimer disease was nutritionally dense and rich in vegetables and avocado. Current developments in nutrition. 2019;3(4):nzz003.

272. Thackray AE, Willis SA, Clayton DJ, Broom DR, Finlayson G, Goltz FR, et al. Influence of short-term hyperenergetic, high-fat feeding on appetite, appetite-related hormones, and food reward in healthy men. Nutrients. 2020;12(9):2635.

273. Vahabzadeh D, Hasanloei MAV, Shariatpanahi ZV. Effect of high-fat, low-carbohydrate enteral formula versus standard enteral formula in hyperglycemic critically ill patients: a randomized clinical trial. Int J Diabetes Dev Ctries. 2019;39(1):173-80.

274. Valsdottir TD, Henriksen C, Odden N, Nellemann B, Jeppesen PB, Hisdal J, et al. Effect of a low-carbohydrate high-fat diet and a single bout of exercise on glucose tolerance, lipid profile and endothelial function in normal weight young healthy females. Front Physiol. 2019;10:1499.

275. Vargas S, Romance R, Petro JL, Bonilla DA, Galancho I, Espinar S, et al. Efficacy of ketogenic diet on body composition during resistance training in trained men: a randomized controlled trial. J Int Soc Sports Nutr. 2018;15(1):1-9.

276. Vargas-Molina S, Carbone L, Romance R, Petro JL, Schoenfeld BJ, Kreider RB, et al. Effects of a low-carbohydrate ketogenic diet on health parameters in resistance-trained women. Eur J Appl Physiol. 2021:1-11.

277. Volek JS, Sharman MJ, Gomez AL, Scheett TP, Kraemer WJ. An isoenergetic very low carbohydrate diet improves serum HDL cholesterol and triacylglycerol concentrations, the total cholesterol to HDL cholesterol ratio and postprandial lipemic responses compared with a low fat diet in normal weight, normolipidemic women. The Journal of nutrition. 2003;133(9):2756-61.

278. Voss M, Wagner M, von Mettenheim N, Harter PN, Wenger KJ, Franz K, et al. ERGO2: A Prospective, Randomized Trial of Calorie-Restricted Ketogenic Diet and Fasting in Addition to Reirradiation for Malignant Glioma. International Journal of Radiation Oncology* Biology* Physics. 2020;108(4):987-95.

279. Waldman HS, Smith JW, Lamberth J, Fountain BJ, McAllister MJ. A 28-day carbohydrate-restricted diet improves markers of cardiometabolic health and performance in professional firefighters. The Journal of Strength & Conditioning Research. 2019;33(12):3284-94.

280. Wan Y, Wang F, Yuan J, Li J, Jiang D, Zhang J, et al. Effects of dietary fat on gut microbiota and faecal metabolites, and their relationship with cardiometabolic risk factors: a 6-month randomised controlled-feeding trial. Gut. 2019;68(8):1417-29.

281. Wan Y, Yuan J, Li J, Li H, Zhang J, Tang J, et al. Unconjugated and secondary bile acid profiles in response to higher-fat, lower-carbohydrate diet and associated with related gut microbiota: A 6-month randomized controlled-feeding trial. Clin Nutr. 2020;39(2):395-404.

282. Wei Y, Zhang H, Zhang S, Li H. The influence of diet upon liver function indices of healthy volunteers resident in a Phase I clinical trail. American journal of translational research. 2019;11(5):3187.

283. Wenger KJ, Wagner M, Harter PN, Franz K, Bojunga J, Fokas E, et al. Maintenance of Energy Homeostasis during Calorically Restricted Ketogenic Diet and Fasting-MR-Spectroscopic Insights from the ERGO2 Trial. Cancers (Basel). 2020;12(12):3549.

284. Wilson JM, Lowery RP, Roberts MD, Sharp MH, Joy JM, Shields KA, et al. Effects of ketogenic dieting on body composition, strength, power, and hormonal profiles in resistance training men. The Journal of Strength & Conditioning Research. 2020;34(12):3463-74.

285. Wolfe BM, Piche LA. Replacement of carbohydrate by protein in conventional-fat diet reduces cholesterol and triglyceride concentrations in healthy normolipidemic subjects. Clinical and investigative medicine. 1999;22(4):140.

286. Yamada Y, Uchida J, Izumi H, Tsukamoto Y, Inoue G, Watanabe Y, et al. A non-calorie-restricted low-carbohydrate diet is effective as an alternative therapy for patients with type 2 diabetes. Intern Med. 2014;53(1):13-9.

287. Zeng N, Prodhan U, d’Souza R, Ramzan F, Mitchell S, Sharma P, et al. Regulation of amino acid transporters and sensors in response to a high protein diet: A randomized controlled trial in elderly men. J Nutr Health Aging. 2019;23(4):354-63.

288. 事例報告, 伊藤翔, 唐沢康暉, 星野太佑, 藤井雅史, 衛藤樹, et al. Effects of low-carbohydrate diet and resistance exercise training on physical characteristics and concentrations of plasma metabolites and hormones. Jpn J Phys Fitness Sports Med. 2019;68(3):223-7.

289. Araújo MLD, Barreto CdCL, Lima COdMF, Marcelino JV, da Silva Diniz A, Cabral PC, et al. Estudo randomizado de intervenção com dieta hiperproteica vs dieta de alto teor de carboidrato em idosas com excesso de peso submetidas a treino de força. Nutrición clínica y dietética hospitalaria. 2020;40(1):149-53.

290. de Luis DA, Izaola O, Primo D, Aller R. Efecto del polimorfismo rs10830963 MTNR1B y la composición de grasa de la dieta en la resistencia a la insulina tras la pérdida de peso durante 3 meses. Endocrinología, Diabetes y Nutrición. 2020;67(1):43-52.

291. Resch K-L. Dietary Intervention Randomized Controlled Trial (DIRECT) group: weight loss with a low-carbohydrate, Mediterranean, or low-fat diet. Forschende Komplementarmedizin (2006). 2008;15(6):351-2.

292. Alzahrani AH, Skytte MJ, Samkani A, Thomsen MN, Astrup A, Ritz C, et al. Body weight and metabolic risk factors in patients with type 2 diabetes on a self-selected high-protein low-carbohydrate diet. Eur J Nutr. 2021:1-10.

293. Alzahrani AH, Skytte MJ, Samkani A, Thomsen MN, Astrup A, Ritz C, et al. Effects of a Self-Prepared Carbohydrate-Reduced High-Protein Diet on Cardiovascular Disease Risk Markers in Patients with Type 2 Diabetes. Nutrients. 2021;13(5):1694.

294. Belopolsky Y, Khan MQ, Sonnenberg A, Davidson DJ, Fimmel CJ. Ketogenic, hypocaloric diet improves nonalcoholic steatohepatitis. Journal of Translational Internal Medicine. 2020;8(1):26.

295. de la Iglesia R, Lopez-Legarrea P, Abete I, Bondia-Pons I, Navas-Carretero S, Forga L, et al. A new dietary strategy for long-term treatment of the metabolic syndrome is compared with the American Heart Association (AHA) guidelines: the MEtabolic Syndrome REduction in NAvarra (RESMENA) project. Br J Nutr. 2014;111(4):643-52.

296. Freedland SJ, Allen J, Jarman A, Oyekunle T, Armstrong AJ, Moul JW, et al. A randomized controlled trial of a 6-month low-carbohydrate intervention on disease progression in men with recurrent prostate cancer: carbohydrate and prostate study 2 (CAPS2). Clin Cancer Res. 2020;26(12):3035-43.

297. Freedland SJ, Howard L, Allen J, Smith J, Stout J, Aronson W, et al. A lifestyle intervention of weight loss via a low-carbohydrate diet plus walking to reduce metabolic disturbances caused by androgen deprivation therapy among prostate cancer patients: carbohydrate and prostate study 1 (CAPS1) randomized controlled trial. Prostate Cancer Prostatic Dis. 2019;22(3):428-37.

298. Goldenshluger A, Constantini K, Goldstein N, Shelef I, Schwarzfuchs D, Zelicha H, et al. Effect of Dietary Strategies on Respiratory Quotient and Its Association with Clinical Parameters and Organ Fat Loss: A Randomized Controlled Trial. Nutrients. 2021;13(7):2230.

299. Guldbrand H, Dizdar B, Bunjaku B, Lindström T, Bachrach-Lindström M, Fredrikson M, et al. In type 2 diabetes, randomisation to advice to follow a low-carbohydrate diet transiently improves glycaemic control compared with advice to follow a low-fat diet producing a similar weight loss. Diabetologia. 2012;55(8):2118-27.

300. Guldbrand H, Lindström T, Dizdar B, Bunjaku B, Östgren CJ, Nystrom F, et al. Randomization to a low-carbohydrate diet advice improves health related quality of life compared with a low-fat diet at similar weight-loss in Type 2 diabetes mellitus. Diabetes Res Clin Pract. 2014;106(2):221-7.

301. Jonasson L, Guldbrand H, Lundberg AK, Nystrom FH. Advice to follow a low-carbohydrate diet has a favourable impact on low-grade inflammation in type 2 diabetes compared with advice to follow a low-fat diet. Ann Med. 2014;46(3):182-7.

302. Meir AY, Keller M, Bernhart SH, Rinott E, Tsaban G, Zelicha H, et al. Lifestyle weight-loss intervention may attenuate methylation aging: the CENTRAL MRI randomized controlled trial. Clin Epigenetics. 2021;13(1):1-10.

303. Milne RM, Mann JI, Chisholm AW, Williams SM. Long-term comparison of three dietary prescriptions in the treatment of NIDDM. Diabetes Care. 1994;17(1):74-80.

304. Skytte MJ, Samkani A, Petersen AD, Thomsen MN, Astrup A, Chabanova E, et al. A carbohydrate-reduced high-protein diet improves HbA 1c and liver fat content in weight stable participants with type 2 diabetes: A randomised controlled trial. Diabetologia. 2019;62(11):2066-78.

305. Strath LJ, Jones CD, Philip George A, Lukens SL, Morrison SA, Soleymani T, et al. The effect of low-carbohydrate and low-fat diets on pain in individuals with knee osteoarthritis. Pain Med. 2020;21(1):150-60.

306. Walker KZ, O'Dea K, Nicholson GC, Muir JG. Dietary composition, body weight, and NIDDM: comparison of high-fiber, high-carbohydrate, and modified-fat diets. Diabetes Care. 1995;18(3):401-3.

307. Wood RJ, Gregory SM, Sawyer J, Milch CM, Matthews TD, Headley SA. Preservation of fat-free mass after two distinct weight loss diets with and without progressive resistance exercise. Metab Syndr Relat Disord. 2012;10(3):167-74.

308. Akhavan NS, Pourafshar S, Johnson SA, Foley EM, George KS, Munoz J, et al. The Relationship between Protein Intake and Source on Factors Associated with Glycemic Control in Individuals with Prediabetes and Type 2 Diabetes. Nutrients. 2020;12(7):2031.

309. Gutiérrez-Repiso C, Molina-Vega M, Bernal-López MR, Garrido-Sánchez L, García-Almeida JM, Sajoux I, et al. Different Weight Loss Intervention Approaches Reveal a Lack of a Common Pattern of Gut Microbiota Changes. Journal of personalized medicine. 2021;11(2):109.

310. Hannon BA, Edwards CG, Thompson SV, Burke SK, Burd NA, Holscher HD, et al. Genetic Variants in Lipid Metabolism Pathways Interact with Diet to Influence Blood Lipid Concentrations in Adults with Overweight and Obesity. Lifestyle Genomics. 2020;13(6):155-63.

311. Haufe S, Haas V, Utz W, Birkenfeld AL, Jeran S, Böhnke J, et al. Long-lasting improvements in liver fat and metabolism despite body weight regain after dietary weight loss. Diabetes Care. 2013;36(11):3786-92.

312. Pan K, Luo J, Aragaki AK, Chlebowski RT. Weight loss, diet composition and breast cancer incidence and outcome in postmenopausal women. Oncotarget. 2019;10(33):3088.

313. Shih CW, Hauser ME, Aronica L, Rigdon J, Gardner CD. Changes in blood lipid concentrations associated with changes in intake of dietary saturated fat in the context of a healthy low-carbohydrate weight-loss diet: a secondary analysis of the Diet Intervention Examining The Factors Interacting with Treatment Success (DIETFITS) trial. The American journal of clinical nutrition. 2019;109(2):433-41.

314. Unwin DJ, Tobin SD, Murray SW, Delon C, Brady AJ. Substantial and sustained improvements in blood pressure, weight and lipid profiles from a carbohydrate restricted diet: an observational study of insulin resistant patients in primary care. Int J Environ Res Public Health. 2019;16(15):2680.

315. Vergara M, Hauser ME, Aronica L, Rigdon J, Fielding-Singh P, Shih CW, et al. Associations of Changes in Blood Lipid Concentrations with Changes in Dietary Cholesterol Intake in the Context of a Healthy Low-Carbohydrate Weight Loss Diet: A Secondary Analysis of the DIETFITS Trial. Nutrients. 2021;13(6):1935.

316. Vranceanu M, Pickering C, Filip L, Pralea IE, Sundaram S, Al-Saleh A, et al. A comparison of a ketogenic diet with a LowGI/nutrigenetic diet over 6 months for weight loss and 18-month follow-up. BMC nutrition. 2020;6(1):1-12.

317. Aude YW, Agatston AS, Lopez-Jimenez F, Lieberman EH, Almon M, Almon M, et al. The national cholesterol education program diet vs a diet lower in carbohydrates and higher in protein and monounsaturated fat: a randomized trial. Arch Intern Med. 2004;164(19):2141-6.

318. Brynes AE, Edwards CM, Ghatei MA, Dornhorst A, Morgan LM, Bloom SR, et al. A randomised four-intervention crossover study investigating the effect of carbohydrates on daytime profiles of insulin, glucose, non-esterified fatty acids and triacylglycerols in middle-aged men. Br J Nutr. 2003;89(2):207-18.

319. Akter S, Dawson JA, Kahathuduwa CN, Chin SH, Binks M. Psychological and weight history variables as predictors of short‐term weight and body fat mass loss. Obesity science & practice. 2020;6(2):152-61.

320. Albanese A, Prevedello L, Markovich M, Busetto L, Vettor R, Foletto M. Pre-operative very low calorie ketogenic diet (VLCKD) vs. very low calorie diet (VLCD): surgical impact. Obes Surg. 2019;29(1):292-6.

321. Davenport L, Johari Y, Klejn A, Laurie C, Smith A, Ooi GJ, et al. Improving Compliance with Very Low Energy Diets (VLEDs) Prior to Bariatric Surgery—a Randomised Controlled Trial of Two Formulations. Obes Surg. 2019;29(9):2750-7.

322. Leonetti F, Campanile FC, Coccia F, Capoccia D, Alessandroni L, Puzziello A, et al. Very low-carbohydrate ketogenic diet before bariatric surgery: prospective evaluation of a sequential diet. Obes Surg. 2015;25(1):64-71.

323. Lodi A, Zarantonello L, Bisiacchi PS, Cenci L, Paoli A. Ketonemia and Glycemia Affect Appetite Levels and Executive Functions in Overweight Females During Two Ketogenic Diets. Obesity. 2020;28(10):1868-77.

324. Ministrini S, Calzini L, Nulli Migliola E, Ricci MA, Roscini AR, Siepi D, et al. Lysosomal acid lipase as a molecular target of the very low carbohydrate ketogenic diet in morbidly obese patients: the potential effects on liver steatosis and cardiovascular risk factors. Journal of clinical medicine. 2019;8(5):621.

325. Myette-Côté É, Durrer C, Neudorf H, Bammert TD, Botezelli JD, Johnson JD, et al. The effect of a short-term low-carbohydrate, high-fat diet with or without postmeal walks on glycemic control and inflammation in type 2 diabetes: a randomized trial. American Journal of Physiology-Regulatory, Integrative and Comparative Physiology. 2018;315(6):R1210-R9.

326. Smeets ET, Mensink RP, Joris PJ. Dietary macronutrients do not differently affect postprandial vascular endothelial function in apparently healthy overweight and slightly obese men. Eur J Nutr. 2021;60(3):1443-51.

327. Tsintzas K, Jones R, Pabla P, Mallinson J, Barrett DA, Kim D-H, et al. Effect of acute and short-term dietary fat ingestion on postprandial skeletal muscle protein synthesis rates in middle-aged, overweight, and obese men. American Journal of Physiology-Endocrinology and Metabolism. 2020;318(3):E417-E29.

328. White AM, Johnston CS, Swan PD, Tjonn SL, Sears B. Blood ketones are directly related to fatigue and perceived effort during exercise in overweight adults adhering to low-carbohydrate diets for weight loss: a pilot study. J Am Diet Assoc. 2007;107(10):1792-6.

329. Assmann TS, Riezu‐Boj JI, Milagro FI, Martínez JA. Circulating adiposity‐related microRNAs as predictors of the response to a low‐fat diet in subjects with obesity. J Cell Mol Med. 2020;24(5):2956-67.

330. Davis NJ, Crandall JP, Gajavelli S, Berman JW, Tomuta N, Wylie-Rosett J, et al. Differential effects of low-carbohydrate and low-fat diets on inflammation and endothelial function in diabetes. J Diabetes Complications. 2011;25(6):371-6.

331. Dyson P, Beatty S, Matthews D. A low‐carbohydrate diet is more effective in reducing body weight than healthy eating in both diabetic and non‐diabetic subjects. Diabet Med. 2007;24(12):1430-5.

332. Foster GD, Wyatt HR, Hill JO, McGuckin BG, Brill C, Mohammed BS, et al. A randomized trial of a low-carbohydrate diet for obesity. N Engl J Med. 2003;348(21):2082-90.

333. Landry MJ, Crimarco A, Perelman D, Durand LR, Petlura C, Aronica L, et al. Adherence to Ketogenic and Mediterranean Study Diets in a Crossover Trial: The Keto–Med Randomized Trial. Nutrients. 2021;13(3):967.

334. Larsen RN, Mann NJ, Maclean E, Shaw J. The effect of high-protein, low-carbohydrate diets in the treatment of type 2 diabetes: a 12 month randomised controlled trial. Diabetologia. 2011;54(4):731-40.

335. Ruth MR, Port AM, Shah M, Bourland AC, Istfan NW, Nelson KP, et al. Consuming a hypocaloric high fat low carbohydrate diet for 12 weeks lowers C-reactive protein, and raises serum adiponectin and high density lipoprotein-cholesterol in obese subjects. Metabolism. 2013;62(12):1779-87.

336. Stentz FB, Mikhael A, Kineish O, Christman J, Sands C. High protein diet leads to prediabetes remission and positive changes in incretins and cardiovascular risk factors. Nutrition, Metabolism and Cardiovascular Diseases. 2021;31(4):1227-37.

337. Maekawa S, Niizawa M, Harada M. A comparison of the weight loss effect between a low-carbohydrate diet and a calorie-restricted diet in combination with intragastric balloon therapy. Intern Med. 2020;59(9):1133-9.

338. Schiavo L, De Stefano G, Persico F, Gargiulo S, Di Spirito F, Griguolo G, et al. A Randomized, Controlled Trial Comparing the Impact of a Low-Calorie Ketogenic vs a Standard Low-Calorie Diet on Fat-Free Mass in Patients Receiving an Elipse™ Intragastric Balloon Treatment. Obes Surg. 2021;31(4):1514-23.

339. Swenson BR, Schulman AS, Edwards MJ, Gross MP, Hedrick TL, Weltman AL, et al. The effect of a low-carbohydrate, high-protein diet on post laparoscopic gastric bypass weight loss: a prospective randomized trial. J Surg Res. 2007;142(2):308-13.

340. Xu C, Markova M, Seebeck N, Loft A, Hornemann S, Gantert T, et al. High‐protein diet more effectively reduces hepatic fat than low‐protein diet despite lower autophagy and FGF21 levels. Liver International. 2020;40(12):2982-97.

341. Basciani S, Costantini D, Contini S, Persichetti A, Watanabe M, Mariani S, et al. Safety and efficacy of a multiphase dietetic protocol with meal replacements including a step with very low calorie diet. Endocrine. 2015;48(3):863-70.

342. Bruci A, Tuccinardi D, Tozzi R, Balena A, Santucci S, Frontani R, et al. Very low-calorie ketogenic diet: a safe and effective tool for weight loss in patients with obesity and mild kidney failure. Nutrients. 2020;12(2):333.

343. Castaldo G, Monaco L, Castaldo L, Galdo G, Cereda E. An observational study of sequential protein-sparing, very low-calorie ketogenic diet (Oloproteic diet) and hypocaloric Mediterranean-like diet for the treatment of obesity. Int J Food Sci Nutr. 2016;67(6):696-706.

344. Castaldo G, Rastrelli L, Galdo G, Molettieri P, Aufiero FR, Cereda E. Aggressive weight-loss program with a ketogenic induction phase for the treatment of chronic plaque psoriasis: A proof-of-concept, single-arm, open-label clinical trial. Nutrition. 2020;74:110757.

345. Dashti HM, Mathew TC, Khadada M, Al-Mousawi M, Talib H, Asfar SK, et al. Beneficial effects of ketogenic diet in obese diabetic subjects. Mol Cell Biochem. 2007;302(1):249-56.

346. Gomez-Arbelaez D, Bellido D, Castro AI, Ordoñez-Mayan L, Carreira J, Galban C, et al. Body composition changes after very-low-calorie ketogenic diet in obesity evaluated by 3 standardized methods. The Journal of Clinical Endocrinology & Metabolism. 2017;102(2):488-98.

347. Griauzde DH, Saslow L, Patterson K, Ansari T, Liestenfeltz B, Tisack A, et al. Mixed methods pilot study of a low-carbohydrate diabetes prevention programme among adults with pre-diabetes in the USA. BMJ open. 2020;10(1):e033397.

348. Jaagura M, Viiard E, Karu‐Lavits K, Adamberg K. Low‐carbohydrate high‐fat weight reduction diet induces changes in human gut microbiota. MicrobiologyOpen. 2021;10(3):e1194.

349. Kalam F, Gabel K, Cienfuegos S, Ezpeleta M, Wiseman E, Varady KA. Alternate Day Fasting Combined with a Low Carbohydrate Diet: Effect on Sleep Quality, Duration, Insomnia Severity and Risk of Obstructive Sleep Apnea in Adults with Obesity. Nutrients. 2021;13(1):211.

350. Kalam F, Gabel K, Cienfuegos S, Wiseman E, Ezpeleta M, Pavlou V, et al. Changes in subjective measures of appetite during 6 months of alternate day fasting with a low carbohydrate diet. Clinical nutrition ESPEN. 2021;41:417-22.

351. Kalam F, Gabel K, Cienfuegos S, Wiseman E, Ezpeleta M, Steward M, et al. Alternate day fasting combined with a low‐carbohydrate diet for weight loss, weight maintenance, and metabolic disease risk reduction. Obesity science & practice. 2019;5(6):531-9.

352. Kenig S, Petelin A, Vatovec TP, Mohorko N, Jenko-Pražnikar Z. Assessment of micronutrients in a 12-wk ketogenic diet in obese adults. Nutrition. 2019;67:110522.

353. Kong Z, Sun S, Shi Q, Zhang H, Tong TK, Nie J. Short-Term Ketogenic Diet Improves Abdominal Obesity in Overweight/Obese Chinese Young Females. Front Physiol. 2020;11:856.

354. Lyngstad A, Nymo S, Coutinho SR, Rehfeld JF, Truby H, Kulseng B, et al. Investigating the effect of sex and ketosis on weight-loss-induced changes in appetite. The American journal of clinical nutrition. 2019;109(6):1511-8.

355. McKenzie AL, Athinarayanan SJ, McCue JJ, Adams RN, Keyes M, McCarter JP, et al. Type 2 diabetes prevention focused on normalization of glycemia: a Two-Year Pilot Study. Nutrients. 2021;13(3):749.

356. McKenzie AL, Hallberg SJ, Creighton BC, Volk BM, Link TM, Abner MK, et al. A novel intervention including individualized nutritional recommendations reduces hemoglobin A1c level, medication use, and weight in type 2 diabetes. JMIR diabetes. 2017;2(1):e6981.

357. Mohorko N, Černelič-Bizjak M, Poklar-Vatovec T, Grom G, Kenig S, Petelin A, et al. Weight loss, improved physical performance, cognitive function, eating behavior, and metabolic profile in a 12-week ketogenic diet in obese adults. Nutr Res. 2019;62:64-77.

358. Monda V, Polito R, Lovino A, Finaldi A, Valenzano A, Nigro E, et al. Short-term physiological effects of a very low-calorie ketogenic diet: effects on adiponectin levels and inflammatory states. Int J Mol Sci. 2020;21(9):3228.

359. Paoli A, Mancin L, Giacona MC, Bianco A, Caprio M. Effects of a ketogenic diet in overweight women with polycystic ovary syndrome. J Transl Med. 2020;18(1):1-11.

360. Pilone V, Tramontano S, Renzulli M, Romano M, Cobellis L, Berselli T, et al. Metabolic effects, safety, and acceptability of very low-calorie ketogenic dietetic scheme on candidates for bariatric surgery. Surg Obes Relat Dis. 2018;14(7):1013-9.

361. Saboo B, Phatak S, Jethwani P, Patel R, Hasnani D, Panchal D, et al. Intervention of a personalized low-carbohydrate diet to reduce HbA1c level and weight in patients with Type 2 diabetes using seed-based flour as replacement for high-carbohydrate flour and foods. Journal of Diabetology. 2021;12(2):196.

362. Syed-Abdul MM, Hu Q, Jacome-Sosa M, Padilla J, Manrique-Acevedo C, Heimowitz C, et al. Effect of carbohydrate restriction-induced weight loss on aortic pulse wave velocity in overweight men and women. Applied Physiology, Nutrition, and Metabolism. 2018;43(12):1247-56.

363. Thomas ND, Shamanna SB. Impact of calorie restriction on glycemic control in overweight patients with type 2 diabetes mellitus. Int J Diabetes Dev Ctries. 2019;39(3):547-50.

364. Tragni E, Vigna L, Ruscica M, Macchi C, Casula M, Santelia A, et al. Reduction of cardio-metabolic risk and body weight through a multiphasic very-low calorie ketogenic diet program in women with overweight/obesity: A study in a real-world setting. Nutrients. 2021;13(6):1804.

365. Valenzano A, Polito R, Trimigno V, Di Palma A, Moscatelli F, Corso G, et al. Effects of very low calorie ketogenic diet on the orexinergic system, visceral adipose tissue, and ROS production. Antioxidants. 2019;8(12):643.

366. Walton CM, Perry K, Hart RH, Berry SL, Bikman BT. Improvement in glycemic and lipid profiles in type 2 diabetics with a 90-day ketogenic diet. Journal of diabetes research. 2019;2019.

367. Yamada S, Inoue G, Ooyane H, Nishikawa H. Changes in Body Weight, Dysglycemia, and Dyslipidemia After Moderately Low-Carbohydrate Diet Education (LOCABO Challenge Program) Among Workers in Japan. Diabetes, Metabolic Syndrome and Obesity: Targets and Therapy. 2021;14:2863.

368. Yost O, DeJonckheere M, Stonebraker S, Ling G, Buis L, Pop-Busui R, et al. Continuous Glucose Monitoring With Low-Carbohydrate Diet Coaching in Adults With Prediabetes: Mixed Methods Pilot Study. JMIR diabetes. 2020;5(4):e21551.

369. Cai L, Yin J, Ma X, Mo Y, Li C, Lu W, et al. Low-carbohydrate diets lead to greater weight loss and better glucose homeostasis than exercise: a randomized clinical trial. Front Med. 2021;15(3):460-71.

370. Brinkworth G, Luscombe‐Marsh N, Thompson C, Noakes M, Buckley J, Wittert G, et al. Long‐term effects of very low‐carbohydrate and high‐carbohydrate weight‐loss diets on psychological health in obese adults with type 2 diabetes: randomized controlled trial. J Intern Med. 2016;280(4):388-97.

371. Brinkworth G, Noakes M, Keogh J, Luscombe N, Wittert G, Clifton P. Long-term effects of a high-protein, low-carbohydrate diet on weight control and cardiovascular risk markers in obese hyperinsulinemic subjects. Int J Obes. 2004;28(5):661-70.

372. Brinkworth GD, Buckley JD, Noakes M, Clifton PM, Wilson CJ. Long-term effects of a very low-carbohydrate diet and a low-fat diet on mood and cognitive function. Arch Intern Med. 2009;169(20):1873-80.

373. Brinkworth GD, Noakes M, Clifton PM, Buckley JD. Effects of a low carbohydrate weight loss diet on exercise capacity and tolerance in obese subjects. Obesity. 2009;17(10):1916-23.

374. Chen Y, Zhou T, Sun D, Li X, Ma H, Liang Z, et al. Distinct genetic subtypes of adiposity and glycemic changes in response to weight-loss diet intervention: the POUNDS Lost trial. Eur J Nutr. 2021;60(1).

375. Crabtree CD, Kackley ML, Buga A, Fell B, LaFountain RA, Hyde PN, et al. Comparison of Ketogenic Diets with and without Ketone Salts versus a Low-Fat Diet: Liver Fat Responses in Overweight Adults. Nutrients. 2021;13(3):966.

376. Cunha G, de Mello LLC, Hasenstab K, Spina L, Bussade I, Mesiano JMP, et al. MRI estimated changes in visceral adipose tissue and liver fat fraction in patients with obesity during a very low-calorie-ketogenic diet compared to a standard low-calorie diet. Clin Radiol. 2020;75(7):526-32.

377. De Luis D, Aller R, Izaola O, Sagrado MG, Conde R. Modulation of insulin concentrations and metabolic parameters in obese patients by− 55CT polymorphism of the UCP3 gene secondary to two hypocaloric diets. Horm Metab Res. 2009;41(01):62-6.

378. de Luis DA, Izaola O, Primo D, Aller R. polymorphism rs16147 of the neuropeptide Y gene modifies the response of cardiovascular risk biomarkers and adipokines to two hypocaloric diets. Lifestyle Genomics. 2017;10(1-2):63-72.

379. Deluis DA, Sagrado MG, Aller R, Izaola O, Conde R. Effects of C358A missense polymorphism of the degrading enzyme fatty acid amide hydrolase on weight loss, adipocytokines, and insulin resistance after 2 hypocaloric diets. Metabolism. 2010;59(9):1387-92.

380. Ditschuneit HH, Flechtner-Mors M, Johnson TD, Adler G. Metabolic and weight-loss effects of a long-term dietary intervention in obese patients. The American journal of clinical nutrition. 1999;69(2):198-204.

381. Flechtner‐Mors M, Ditschuneit HH, Johnson TD, Suchard MA, Adler G. Metabolic and weight loss effects of long‐term dietary intervention in obese patients: four‐year results. Obes Res. 2000;8(5):399-402.

382. Fogelholm M, Larsen TM, Westerterp-Plantenga M, Macdonald I, Martinez JA, Boyadjieva N, et al. PREVIEW: prevention of diabetes through lifestyle intervention and population studies in Europe and around the world. Design, methods, and baseline participant description of an adult cohort enrolled into a three-year randomised clinical trial. Nutrients. 2017;9(6):632.

383. Friedman AN, Ogden LG, Foster GD, Klein S, Stein R, Miller B, et al. Comparative effects of low-carbohydrate high-protein versus low-fat diets on the kidney. Clin J Am Soc Nephrol. 2012;7(7):1103-11.

384. Galletly C, Moran L, Noakes M, Clifton P, Tomlinson L, Norman R. Psychological benefits of a high-protein, low-carbohydrate diet in obese women with polycystic ovary syndrome—a pilot study. Appetite. 2007;49(3):590-3.

385. Gardner CD, Trepanowski JF, Del Gobbo LC, Hauser ME, Rigdon J, Ioannidis JP, et al. Effect of low-fat vs low-carbohydrate diet on 12-month weight loss in overweight adults and the association with genotype pattern or insulin secretion: the DIETFITS randomized clinical trial. JAMA. 2018;319(7):667-79.

386. Goni L, Riezu-Boj JI, Milagro FI, Corrales FJ, Ortiz L, Cuervo M, et al. Interaction between an ADCY3 genetic variant and two weight-lowering diets affecting body fatness and body composition outcomes depending on macronutrient distribution: a randomized trial. Nutrients. 2018;10(6):789.

387. Greenberg I, Stampfer MJ, Schwarzfuchs D, Shai I, group D. Adherence and success in long-term weight loss diets: the dietary intervention randomized controlled trial (DIRECT). J Am Coll Nutr. 2009;28(2):159-68.

388. Halyburton AK, Brinkworth GD, Wilson CJ, Noakes M, Buckley JD, Keogh JB, et al. Low-and high-carbohydrate weight-loss diets have similar effects on mood but not cognitive performance. The American journal of clinical nutrition. 2007;86(3):580-7.

389. Hjorth MF, Bray GA, Zohar Y, Urban L, Miketinas DC, Williamson DA, et al. Pretreatment fasting glucose and insulin as determinants of weight loss on diets varying in macronutrients and dietary fibers—the POUNDS LOST study. Nutrients. 2019;11(3):586.

390. Hu T, Yao L, Reynolds K, Whelton PK, Niu T, Li S, et al. The effects of a low-carbohydrate diet vs. a low-fat diet on novel cardiovascular risk factors: a randomized controlled trial. Nutrients. 2015;7(9):7978-94.

391. Jenkins DJ, Wong JM, Kendall CW, Esfahani A, Ng VW, Leong TC, et al. The effect of a plant-based low-carbohydrate (“Eco-Atkins”) diet on body weight and blood lipid concentrations in hyperlipidemic subjects. Arch Intern Med. 2009;169(11):1046-54.

392. Layman DK, Shiue H, Sather C, Erickson DJ, Baum J. Increased dietary protein modifies glucose and insulin homeostasis in adult women during weight loss. The Journal of nutrition. 2003;133(2):405-10.

393. Mayer SB, Jeffreys AS, Olsen MK, McDuffie JR, Feinglos MN, Yancy Jr WS. Two diets with different haemoglobin A1c and antiglycaemic medication effects despite similar weight loss in type 2 diabetes. Diabetes, Obesity and Metabolism. 2014;16(1):90-3.

394. Moore SD, King AC, Kiernan M, Gardner CD. Outcome expectations and realizations as predictors of weight regain among dieters. Eating behaviors. 2011;12(1):60-3.

395. Moreno B, Crujeiras AB, Bellido D, Sajoux I, Casanueva FF. Obesity treatment by very low-calorie-ketogenic diet at two years: reduction in visceral fat and on the burden of disease. Endocrine. 2016;54(3):681-90.

396. Morgan LM, Griffin B, Millward D, DeLooy A, Fox K, Baic S, et al. Comparison of the effects of four commercially available weight-loss programmes on lipid-based cardiovascular risk factors. Public Health Nutr. 2009;12(6):799-807.

397. Motie M, Evangelista LS, Horwich T, Hamilton M, Lombardo D, Cooper DM, et al. Pro-HEART—a randomized clinical trial to test the effectiveness of a high protein diet targeting obese individuals with heart failure: rationale, design and baseline characteristics. Contemp Clin Trials. 2013;36(2):371-81.

398. Parker B, Noakes M, Luscombe N, Clifton P. Effect of a high-protein, high–monounsaturated fat weight loss diet on glycemic control and lipid levels in type 2 diabetes. Diabetes Care. 2002;25(3):425-30.

399. Pedersen E, Jesudason D, Clifton P. High protein weight loss diets in obese subjects with type 2 diabetes mellitus. Nutrition, Metabolism and Cardiovascular Diseases. 2014;24(5):554-62.

400. Pittas AG, Roberts SB, Das SK, Gilhooly CH, Saltzman E, Golden J, et al. The effects of the dietary glycemic load on type 2 diabetes risk factors during weight loss. Obesity. 2006;14(12):2200-9.

401. Porter Starr KN, Orenduff M, McDonald SR, Mulder H, Sloane R, Pieper CF, et al. Influence of weight reduction and enhanced protein intake on biomarkers of inflammation in older adults with obesity. J Nutr Gerontol Geriatr. 2019;38(1):33-49.

402. Porter Starr KN, Pieper CF, Orenduff MC, McDonald SR, McClure LB, Zhou R, et al. Improved function with enhanced protein intake per meal: a pilot study of weight reduction in frail, obese older adults. Journals of Gerontology Series A: Biomedical Sciences and Medical Sciences. 2016;71(10):1369-75.

403. Sacks FM, Bray GA, Carey VJ, Smith SR, Ryan DH, Anton SD, et al. Comparison of weight-loss diets with different compositions of fat, protein, and carbohydrates. N Engl J Med. 2009;360(9):859-73.

404. Samaha FF, Iqbal N, Seshadri P, Chicano KL, Daily DA, McGrory J, et al. A low-carbohydrate as compared with a low-fat diet in severe obesity. N Engl J Med. 2003;348(21):2074-81.

405. Saslow LR, Kim S, Daubenmier JJ, Moskowitz JT, Phinney SD, Goldman V, et al. A randomized pilot trial of a moderate carbohydrate diet compared to a very low carbohydrate diet in overweight or obese individuals with type 2 diabetes mellitus or prediabetes. PloS one. 2014;9(4):e91027.

406. Seshadri P, Iqbal N, Stern L, Williams M, Chicano KL, Daily DA, et al. A randomized study comparing the effects of a low-carbohydrate diet and a conventional diet on lipoprotein subfractions and C-reactive protein levels in patients with severe obesity. The American journal of medicine. 2004;117(6):398-405.

407. Stern L, Iqbal N, Seshadri P, Chicano KL, Daily DA, McGrory J, et al. The effects of low-carbohydrate versus conventional weight loss diets in severely obese adults: one-year follow-up of a randomized trial. Ann Intern Med. 2004;140(10):778-85.

408. Struik NA, Brinkworth GD, Thompson CH, Buckley JD, Wittert G, Luscombe-Marsh ND. Very low and higher carbohydrate diets promote differential appetite responses in adults with type 2 diabetes: a randomized trial. The Journal of nutrition. 2020;150(4):800-5.

409. Tay J, Brinkworth GD, Noakes M, Keogh J, Clifton PM. Metabolic effects of weight loss on a very-low-carbohydrate diet compared with an isocaloric high-carbohydrate diet in abdominally obese subjects. J Am Coll Cardiol. 2008;51(1):59-67.

410. Tay J, Luscombe-Marsh ND, Thompson CH, Noakes M, Buckley JD, Wittert GA, et al. Comparison of low-and high-carbohydrate diets for type 2 diabetes management: a randomized trial. The American journal of clinical nutrition. 2015;102(4):780-90.

411. Tay J, Luscombe-Marsh ND, Thompson CH, Noakes M, Buckley JD, Wittert GA, et al. A very low-carbohydrate, low–saturated fat diet for type 2 diabetes management: a randomized trial. Diabetes Care. 2014;37(11):2909-18.

412. Tay J, Thompson CH, Luscombe‐Marsh ND, Wycherley TP, Noakes M, Buckley JD, et al. Effects of an energy‐restricted low‐carbohydrate, high unsaturated fat/low saturated fat diet versus a high‐carbohydrate, low‐fat diet in type 2 diabetes: A 2‐year randomized clinical trial. Diabetes, Obesity and Metabolism. 2018;20(4):858-71.

413. Tay J, Zajac IT, Thompson CH, Luscombe-Marsh ND, Danthiir V, Noakes M, et al. A randomised-controlled trial of the effects of very low-carbohydrate and high-carbohydrate diets on cognitive performance in patients with type 2 diabetes. Br J Nutr. 2016;116(10):1745-53.

414. Tirosh A, Golan R, Harman-Boehm I, Henkin Y, Schwarzfuchs D, Rudich A, et al. Renal function following three distinct weight loss dietary strategies during 2 years of a randomized controlled trial. Diabetes Care. 2013;36(8):2225-32.

415. Volek JS, Ballard KD, Silvestre R, Judelson DA, Quann EE, Forsythe CE, et al. Effects of dietary carbohydrate restriction versus low-fat diet on flow-mediated dilation. Metabolism. 2009;58(12):1769-77.

416. Watson NA, Dyer KA, Buckley JD, Brinkworth GD, Coates AM, Parfitt G, et al. Comparison of two low-fat diets, differing in protein and carbohydrate, on psychological wellbeing in adults with obesity and type 2 diabetes: a randomised clinical trial. Nutr J. 2018;17(1):1-12.

417. Weaver AA, Houston DK, Shapses SA, Lyles MF, Henderson RM, Beavers DP, et al. Effect of a hypocaloric, nutritionally complete, higher-protein meal plan on bone density and quality in older adults with obesity: a randomized trial. The American journal of clinical nutrition. 2019;109(2):478-86.

418. Westman EC, Yancy Jr WS, Olsen MK, Dudley T, Guyton JR. Effect of a low-carbohydrate, ketogenic diet program compared to a low-fat diet on fasting lipoprotein subclasses. Int J Cardiol. 2006;110(2):212-6.

419. Yancy WS, Almirall D, Maciejewski ML, Kolotkin RL, McDuffie JR, Westman EC. Effects of two weight-loss diets on health-related quality of life. Qual Life Res. 2009;18(3):281-9.

420. Yokose C, McCormick N, Rai SK, Lu N, Curhan G, Schwarzfuchs D, et al. Effects of low-fat, Mediterranean, or low-carbohydrate weight loss diets on serum urate and cardiometabolic risk factors: a secondary analysis of the dietary intervention randomized controlled trial (direct). Diabetes Care. 2020;43(11):2812-20.
